# Comprehensive computational automated search of barrierless reactions leading to the formation of benzene and other C_6_-membered rings

**DOI:** 10.1126/sciadv.adq4077

**Published:** 2024-09-11

**Authors:** Marta Castiñeira Reis, Emilio Martínez Núñez, Antonio Fernández Ramos

**Affiliations:** ^1^ Centro Singular de Investigación en Química Biolóxica e Materiais Moleculares (CIQUS), Campus Vida, 15782, Universidade de Santiago de Compostela, C/Jenaro de la Fuente s/n, Santiago de Compostela, Spain.; ^2^ Departamento de Química Física, Facultade de Química, Campus Vida, 15782, Universidade de Santiago de Compostela, Avda. das Ciencias s/n, Santiago de Compostela, Spain.

## Abstract

We present the systematic exploration of various potential energy surfaces for systems with C_6_H_6–*x*_ (*x* = 0, 1, 2, or 3) empirical formula using an automatic search approach. The primary objective of this study is to identify reaction pathways that lead to the creation of benzene, *o*-benzyne, and other rings. These pathways initiate with a barrierless recombination reaction and involve subsequent isomerization reactions with submerged transition states until the final product is reached. The reported reaction profiles are consistent with the existing conditions in the interstellar medium if the hot complex formed can cool down through radiative relaxation. Recent studies on the deactivation of polyaromatic hydrocarbons (PAHs) support the possibility of these reactions taking place. The C_6_-membered rings are considered precursors of PAHs, and our focus is on identifying pathways originating from the barrierless recombination of reactive molecules known to exist in the interstellar medium, with potential implications in other environments.

## Teaser

The reaction pathways for benzene and aromatic rings are identified through barrierless recombination.

## INTRODUCTION

Small hydrocarbon molecules are widely present in combustion chemistry, the atmosphere, and the interstellar medium (ISM). They are thought to play a crucial role in the creation of more complex hydrocarbon molecules, such as benzene (C_6_H_6_) (*1*), *o*-benzyne (*2*), higher-order polyaromatic hydrocarbons (PAHs) such as indene (c-C_9_H_8_) (*3*, *4*), or derivatives such as 2-cyanoindene (2-C_9_H_7_CN) (*5*). It is suggested that around 10 to 25% of interstellar carbon exists in the form of PAHs, indicating their substantial presence in space (*6*, *7*).

The most accepted hypothesis is that PAHs are formed in the ISM from benzene or the phenyl radical through reactions involving acetylene or other carbonaceous scaffolds (*8*–*10*). For instance, the phenyl radical may be key in the formation of PAHs (*10*–*12*) and cyanonaphthalenes (*9*), and the 1,2,3-tridihydrobenzene and the 1-hexene-3,5-diynyl-2 radical (l-H_2_H_6_H) have been obtained in cross-beam experiments of C_2_ with vinylacetylene (*13*). In addition, 1,2,3-tridihydrobenzene (or 1,2,3-cyclohexatriene) due to the ring strain may be key in the synthesis of complex organic molecules (*14*).

One of the pioneering computational works that explore the formation of benzene was carried out by Miller and Klippenstein (*15*). These authors found that benzene could form by recombination of two propargyl (CH_2_CCH) radicals after several isomerization reactions at temperatures between 300 and 2200 K and different pressures. The recent discovery of the propargyl radical in the TMC-1 cold dark cloud (*16*) has strengthened the hypothesis that this radical may be a key species in the formation of benzene. Other species that have a direct relation to dehydrogenated forms of benzene, such as *o*-benzyne, are vinylacetylene(but-1-en-3-yne) (*17*) and other C_4_H_4_ isomers (*18*), which can be formed by reaction with singlet or triplet spin states of the C_2_ molecule (*13*).

Because of the low densities of reactants and ultralow temperatures in the ISM, the recombination reaction between two species must be barrierless, forming a hot intermediate that may dissociate back to reactants or rearrange to produce two different fragments. This reaction of the type A + B → C + D may occur in a one-step process where the products are more stable than the reactants or in a multistep process with submerged (below reactants) transition states (*19*). In principle, the reaction of two propargyl radicals to form benzene seems highly improbable because a hot complex is formed that will dissociate back to reactants or two different products. However, there is also the possibility that the hot complex formed by the collision of A and B loses part of its energy excess leading to only one product. Traditionally, it has been believed that because of the low density of the reacting species, the complex cannot dissipate its energy excess and dissociates back to reactants. However, recent research by Stockett *et al.* (*20*) suggests that radiative relaxation is more effective than previously assumed. This process allows the hot complex to cool down efficiently, ultimately forming a cold product. These findings have notable implications, as they explain the resilience of small PAHs, such as cyanonaphthalene and naphthalene (*21*), from dissociating back to reactants. Furthermore, this evidence opens up the possibility of similar reactions occurring for other cyclic species such as benzene and its derivatives. An additional possibility for this type of reaction to occur is on a grain surface because the latter can dissipate the energy excess of the complex.

The aim of this study is to use automated computational methods to explore the potential energy surface of benzene, *o*-benzyne, and other C_6_-membered rings. The objective is to identify a diverse variety of barrierless bimolecular reactions of the form A + B → C, which exhibit submerged transition states and could potentially lead to one of the aforementioned species. Our focus will be on reactants that have been observed in the ISM or that have been theorized to exist, even if they have not yet been detected. In this work, we identify all these potential paths, but the kinetic mechanism responsible for stabilizing the final product falls outside the scope of this research.

For the generation of the potential energy surfaces, we rely on software for the automatic search of stationary points. The benefit of using an automated approach lies in its immunity to human bias, thereby allowing for a more uninhibited exploration of the potential energy surface. In this context, several programs have been designed to automatically find multistep reactions (*22*–*30*) and generate intricate reaction networks.

Here, we have used AutoMeKin (*22*, *28*, *31*), which is a computational code designed to study unimolecular decompositions. The program has demonstrated its proficiency in analyzing systems of comparable size to benzene, as evidenced by its application in studying the decomposition of indole (*32*). The automated algorithm identifies transition state structures (saddle points) for both isomerization and decomposition reactions. The equilibrium structures of these reactions are obtained by following the minimum energy path (MEP) from the transition state in the backward direction (toward reactants) and in the forward direction (toward products). This allows building a reaction network about the target species that includes the interconversion reactions between different isomers and the products of their decomposition.

The search for barrierless decompositions is more involved and requires an additional step, as these reactions do not present a transition state. We use the nudged elastic band algorithm (*33*) to investigate fragmentation pathways originating from different isomeric forms of the target molecule. This approach makes it possible to identify previously unobserved transition states and processes without energy barriers. By convenience, we are approaching the problem from a different perspective, i.e., the fragments resulting from barrierless reactions will act as the initial reactants that combine to form the desired product. Notice that the reported potential energy surfaces may also serve as a future database for the study of the interconversion reactions between isomers and their decomposition into different products. All the information about the different reaction networks is available at Zenodo (*34*).

## RESULTS AND DISCUSSION

We follow a general scheme in which each of the C_6_H_6–*x*_ (*x* = 0, 1, 2, or 3) rings are analyzed separately, beginning with benzene and ending with the C_6_H_3_ radicals. For the latter, some six-membered rings and some linear carbon chains have similar energies, so it is at this stage when the rings start to form (*13*). The successive additions of hydrogen atoms are barrierless processes that systematically increase the stability of the ring, starting with the addition to the C_6_H_3_ to form benzyne, continuing with the formation of the phenyl radical, and ending with the formation of benzene. The hydrogen additions to the ring provide a common ground for a systematic study of these carbonaceous rings. In the following sections, first, we describe the formation of benzene; second, the formation of the phenyl radical and of benzyne; and last, we deal with the formation of several isomers with C_6_H_3_ empirical formula.

### Benzene

Shock-tube experiments carried out by Wu and Kern (*35*) found that two propargyl radicals (**PR29**) produce benzene as one of the major products. Miller and Melius (*36*) argued that benzene and the phenyl radical plus a hydrogen atom are the first rings formed after the recombination of the two propargyl radicals. A subsequent series of gas phase (*37*) and combustion studies (*15*, *38*, *39*) confirmed the formation of benzene, as well as that of fulvene and the phenyl radical. In addition, the work of Wilson *et al.* (*40*) that simulated the conditions of the atmosphere of Titan pointed out that the reaction of two propargyl radicals is the main route to benzene formation.

**Fig. 1.**
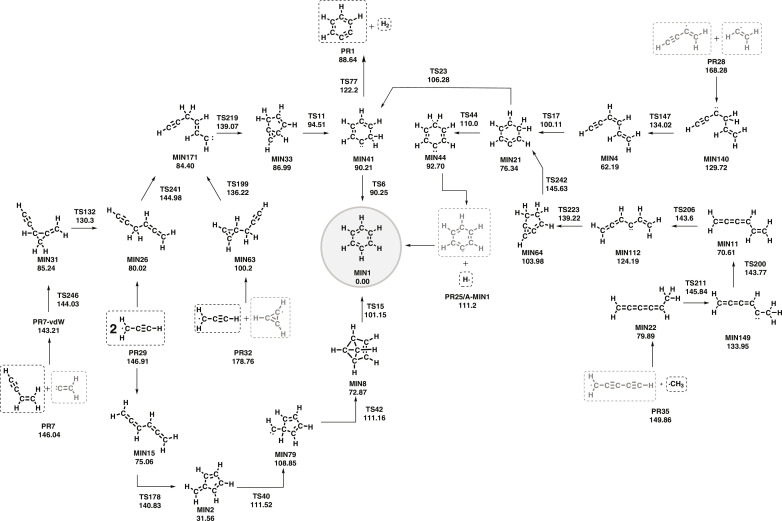
Multistep barrierless reactions encountered by AutoMeKin leading to benzene. In gray are the reactive species that have not been yet detected in the ISM. All F12-CCSD(T) relative energies include the density functional theory (DFT) zero-point energies.

AutoMeKin found 191 minima, 278 transition states, and 35 products of benzene decomposition at the M08HX/6-31G(d,p) level. The reaction network generated from these structures predicts that the recombination of two propargyl radicals is a viable path to benzene formation. Figure 1 shows two of these paths, but more details are given in the Supplementary Materials. One of them leads to the 1,2,4,5-hexatetraene (**MIN15**) and then to fulvene (**MIN2**). The latter is a key species in the recombination of the two propargyl radicals, although it is possible to find a path that avoids fulvene and that passes through the 1,2-hexadiene-5-yne (**MIN26**). Labels of stationary points of the initial benzene network start with MIN or TS for equilibrium or transition state structures, respectively. To distinguish the stationary points that originate from different reaction networks, the prefixes A, B, C, etc., are added to the labels with a given empirical formula using the following convention: A (C_6_H_5_), B (C_6_H_4_), C (C_6_H_3_), D (C_6_H_2_), E (C_4_H_4_), F (C_5_H_3_), G (C_5_H_2_), H (C_5_H), and I (C_4_H_4_).

Benzene can also be formed from the barrierless reaction of the phenyl radical with atomic hydrogen. Vinylacetylene and acetylene can also react to form benzene, but the initial reaction has a barrier of 31.88 kcal mol^−1^, so this path was discarded. When vinylidene (CCH_2_) substitutes acetylene the reaction is barrierless. Unfortunately, this carbene remains elusive to detection in the ISM, although the existence of this reactive intermediate has been hypothesized many years ago (*41*).

We also included C_5_H_3_ species because the recombination reaction of this radical with the methyl group can be obtained from the photodissociation of benzene (*42*); in particular, the excitation of benzene at 193 and 248 nm produces 4% of C_5_H_3_ + CH_3_. All reactions between these two fragments involve the formation of the hexa-1,2,3,4-tetraene (**MIN22**) that evolves by an H-shift to a carbene and undergoes another H-shift to form the hexa-1,2,3,5-tetraene (**MIN11**). This intermediate evolves to a carbene that reacts to form a bicyclic species (**MIN64**) and the subsequent C_6_-membered ring species (**MIN21**), which can evolve to **MIN44** to form the phenyl radical plus atomic hydrogen or to **MIN41** to yield *o*-benzyne plus H_2_ or benzene. However, the high barrier heights involved (near the dissociation energy of the two fragments) and the large number of elementary steps indicate that this path will marginally contribute to the formation of C_6_-membered rings, a result that is in agreement with experimental findings.

The reactions of different C_4_H_4_ isomers (vinyl ethylene, butatriene, and methylenecyclopropene) with acetylene all have initial barriers. The reaction between the vinyl and butatrienyl radicals is barrierless, but none of the two species has been detected in the ISM. A similar situation occurs with the reaction between the propargyl and cyclopropenyl (c-C_3_H_3_) radicals because the latter has not been encountered in the ISM.

### The phenyl radical

**Fig. 2.**
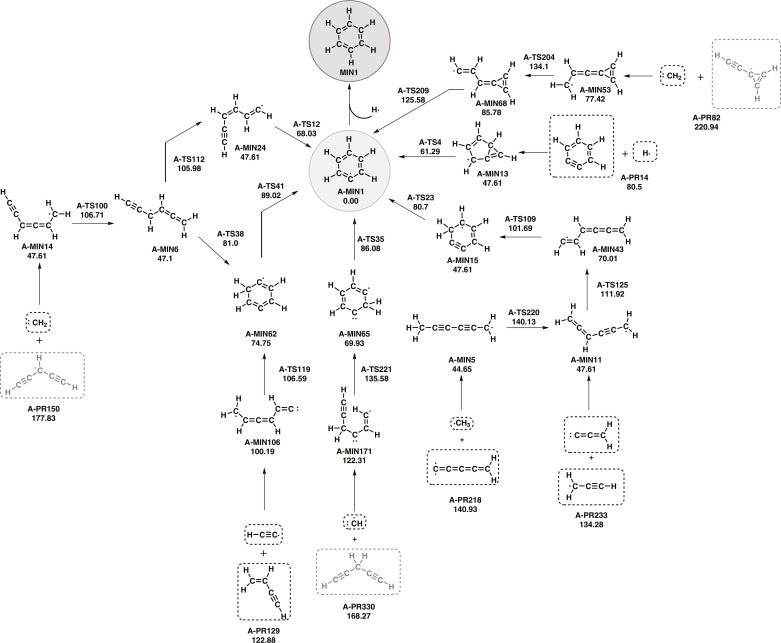
Same as Fig. 1 but for the formation of the phenyl radical.

The number of minima, transition states, and products optimized at the density functional theory (DFT) method are 181, 268, and 356, respectively. The phenyl radical (c-C_6_H_5_) can be formed from the reaction of the hydrogen atom with *o*-benzyne (see Fig. 2), but the reaction occurs in two steps. The initial barrierless reaction leads to an intermediate (**A-MIN13**), with an energy of 32.89 kcal mol^−1^ below reactants, that easily evolves to the final product. However, *o*-benzyne is not the only species with C_6_H_4_ empirical formula that can lead to the phenyl radical. A full list of the currently undetected reactants compatible with the ISM conditions is given in the Supplementary Materials. Alternative paths to the *o*-benzyne route involve additions to a triple bond, such as the addition of the methylidyne radical, methylene (*43*), methyl radical (*44*), ethynyl radical (*45*), and propadienylidene (l-H_2_C_3_) (*46*) to C_5_H_4_, C_5_H_3_, C_5_H_2_, C_4_H_4_, and C_3_H_3_ species, respectively.

The reaction of the methylidyne radical (CH) with 1,4-pentadiyne [HCCCH_2_CCH—an isomer of allenyl acetylene, whose existence in the ISM could not be established (*47*)], allows forming the phenyl radical in just three steps: (i) addition, (ii) cyclization, and (iii) hydrogen shift. The reaction path involves two low-barrier transition states because, in this case, there are no hydrogen shift reactions associated with the acyclic species.

AutoMeKin predicts that two C_5_H_3_ isomers, the 1,4-pentadiynyl-3 and the ethynyl cyclopropenylidene radicals, can react with methylene by an addition to the triple bond. The first C_5_H_3_ isomer forms the **A-MIN14** intermediate species, which undergoes two consecutive hydrogen shift reactions and a further cyclization to produce the final product. The second C_5_H_3_ isomer leads to the phenyl radical in three steps: addition (to form **A-MIN53**), hydrogen shift, and cyclization. More information about the most stable C_5_H_3_ isomers is given in the Supplementary Materials.

The reaction of the ethynyl radical with vinylacetylene offers more possibilities because both molecules present triple bonds. Figure 2 shows one of the paths in which it is possible to obtain the phenyl radical in just three steps, i.e., addition to the triple bond, cyclization, and hydrogen transfer.

The reaction of the propargyl radical with propadienylidene forms an allenyl propargyl radical (**A-MIN11**), which, by hydrogen shift, leads to the intermediate **A-MIN43** that forms a cycle (**A-MIN15**) and undergoes a new hydrogen shift to yield (**A-MIN1**). Another possibility is the addition of the methyl radical to pentatetraenylidene that produces the phenyl radical in five steps, of which the last three are common to the reaction starting with the two propargyl radicals.

### *o*-Benzyne

**Fig. 3.**
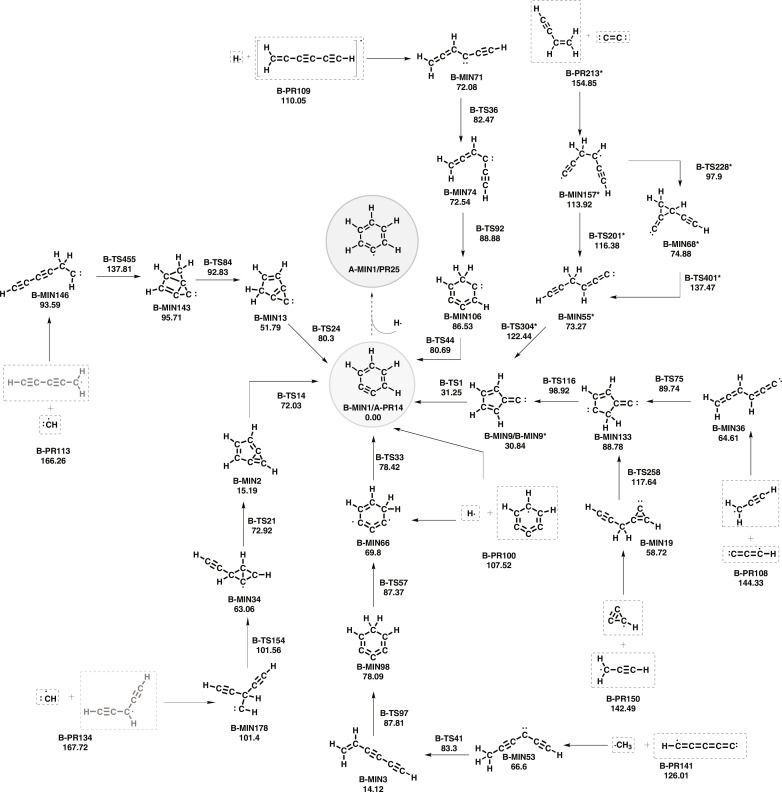
Same as Fig. 1 but for the formation of *o*-benzyne.

Many pathways may lead to *o*-benzyne, and the reaction network is formed by 278 minima, 552 transition states, and 201 products. As shown in Fig. 3, the most straightforward path is through 1,2,3-tridehydrobenzene because it is a barrierless one-step reactions. However, this species has not yet been detected in the ISM.

Another possibility is the reaction of the propargyl radical with l-C_3_H (**B-PR108**) or with c-C_3_H (**B-PR150**). In both cases, there is a first association step with a large stabilization and a cyclization to a common species that suffers an initial hydrogen transfer to form fulvenylidene (**B-MIN9**) and a final (nearly barrierless) ring expansion to produce *o*-benzyne.

The mechanism proposed by Kaiser *et al.* (*13*) that starts with adding C_2_ (singlet) to the terminal carbon of the double bond of vinylacetylene was also found. The recombination reaction leads to **B-MIN157** [**si3**—nomenclature from (*13*)], which, by an internal C─C rotation, leads to **B-MIN55**, and this species undergoes cyclization to form fulvenylidene, which easily evolves to *o*-benzyne (see Fig. 3).

Figure 3 shows that vinyldiacetylene can be formed from the recombination reaction of the methyl radical and pentadiynylidene (l-C_5_H) (*48*) with an energy of 126.01 kcal mol^−1^ above *o*-benzyne. AutoMeKin indicates that l-H_2_C_6_H can be formed from hexapentaenylidene (l-H_2_C_6_) (*49*) plus the hydrogen atom or from butatrienylidene (l-H_2_C_4_) (*50*) plus the ethynyl radical (not displayed in Fig. 3) The formation of *o*-benzyne from 1-hexene-3,5-diynyl-2 (l-H_2_C_6_H) plus atomic hydrogen leads to several possibilities that are explained in more detailed in the Supplementary Materials.

In addition, we encountered that two compounds with C_5_H_3_ empirical formula can recombine with methylidene and lead to *o*-benzyne after a few steps. The first is the 2,4-pentadiynyl-1 radical, already mentioned above, and the other is the 1,4-pentadiynyl-3 radical (HCCCHCCH). The later can be formed from the recombination of the ethynyl radical plus cyclopropenylidene (c-C_3_H_2_) (*51*), followed by a ring opening and H-shift. The 1,4-pentadiynyl-3 radical may also lead to *m*-benzyne (**B-MIN2**) because it is the previous step to the *o*-benzyne formation.

### C_6_H_3_ structures

**Fig. 4.**
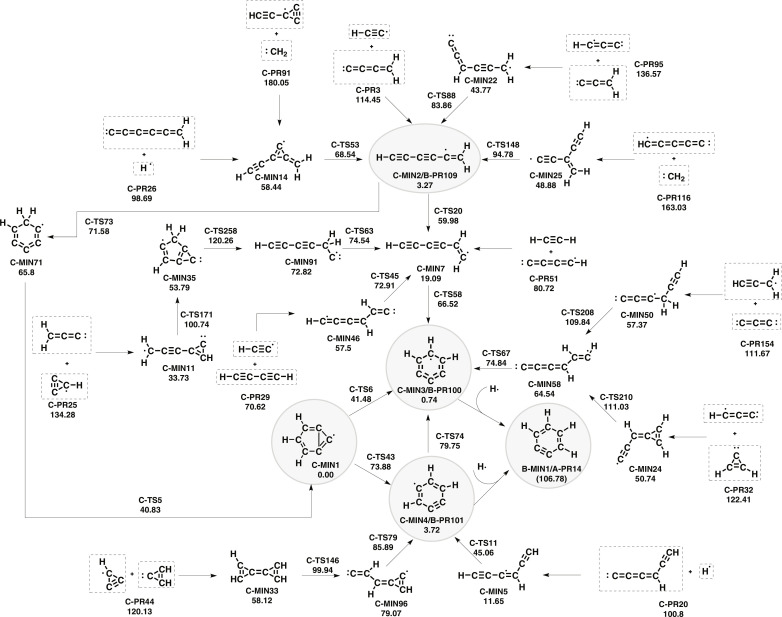
Same as Fig. 1 but for the formation of C_6_H_3_ structures.

The reaction path connections between reactive ISM molecules and the most stable C_6_H_3_ structures are given in Fig. 4. Kaiser *et al.* (*13*) considered the C_6_H_3_ structures “as the crucial link between energetically favored aromatic and resonantly stabilized radicals for hydrogen-rich and poor C_6_H*_x_* molecules, respectively.” Certainly, this is the case for the 6-bicyclo[3.1.0]hexa-1,3,5-trienyl radical (**C-MIN1**), 1,2,3-tridehydrobenzene (**C-MIN3**), and 1,2,4-tridehydrobenzene (**C-MIN4**) that are very close energy to l-H_2_C_6_H (**C-MIN2**). Therefore, molecular structures with C_6_H_3_ empirical formula are the ones with the least number of hydrogen atoms of the type C_6_H*_x_* (where *x* = 1, …, 6) for which the closed ring is very close in energy to the open carbon chain (see Fig. 4). The ring formation is unfavorable from an entropic point of view at high temperatures but competes with the other mechanisms at ultralow temperatures because, in this case, the entropic contribution is negligible.

The linear l-H_2_C_6_H radical can lead to *o*-benzene by a further H-atom addition (vide supra). Crossed molecular beam experiments carried out by Guo *et al.* (*52*) found that this molecule can be formed from C_3_ (*53*) and allene (H_2_CCCH_2_). We found that the 1-hexene-3,5-diynyl-2 radical (Fig. 4) can also be formed by the recombination of (i) hexapentaenylidene plus atomic hydrogen (**C-PR26**), (ii) pentadiynylidene plus methylene (**C-PR116**), (iii) ethynylcyclopropynylidyne (c-C_3_C_2_H) (*54*) plus methylene (**C-PR191**), (iv) butatrienylidene plus the ethynyl radical (**C-PR29**), (v) propadienylidene plus propynylidyne (C_3_H) (*55*) (**C-PR95**), (vi) reactions that involve C_3_-membered rings (**C-PR25**, **C-PR32**, and **C-PR44**), and (vi) other reactants that go through the **C-MIN7** radical. In particular, this radical is a key intermediate between the open and closed forms of C_6_H_3_ structures. It can be reached by recombining the reactants mentioned above and by other three paths. The most direct path is the recombination of acetylene and the butadiynyl radical. Another possibility for the formation of cyclic structures from l-H_2_C_6_H is by direct cyclization through **C-MIN71** and concerted H-shift and C─C bond formation leading to the **C-MIN1** bicyclic structure.

The addition of atomic hydrogen to 1,2,3-tridehydrobenzene and 1,2,4-tridehydrobenzene may also be a source of formation of *o*-benzyne but not the bicyclo[3.1.0]hexa-1,3,5-trienyl radical. Adding atomic or molecular hydrogen to the latter presents a barrier height. A new path starts with the recombination of the propargyl radical and c-C_3_, but c-C_3_ has not been detected in the ISM (not plotted in Fig. 4). Other possibilities involve passing through other stable cyclic structures (**C-MIN3** or **C-MIN4**), but once they are formed, their evolution to **C-MIN1** is highly improbable due to the high barriers involved.

The 1,2,3-tridehydrobenzene radical, apart from the paths that connect with the **C-MIN7** intermediate, can be formed from C_3_ plus the propargyl radical or from propynylidyne (HC_3_) plus cyclopropenylidene (c-C_3_H_2_). Both paths form the cyclic radical in three steps and have in common the intermediate **C-MIN58** in the last step.

The 1,2,4-tridehydrobenzene can be formed from the recombination of cyclopropenylidene and the cyclopropanediylidenyl radical (c-C_3_H) (*56*, *57*) after a ring opening of one of the rings, followed by a ring opening of the carbene and a further cyclization. It can also be formed from ethynylallene (HCCCHCCC) (*58*) plus the hydrogen atom in only two steps.

**Fig. 5.**
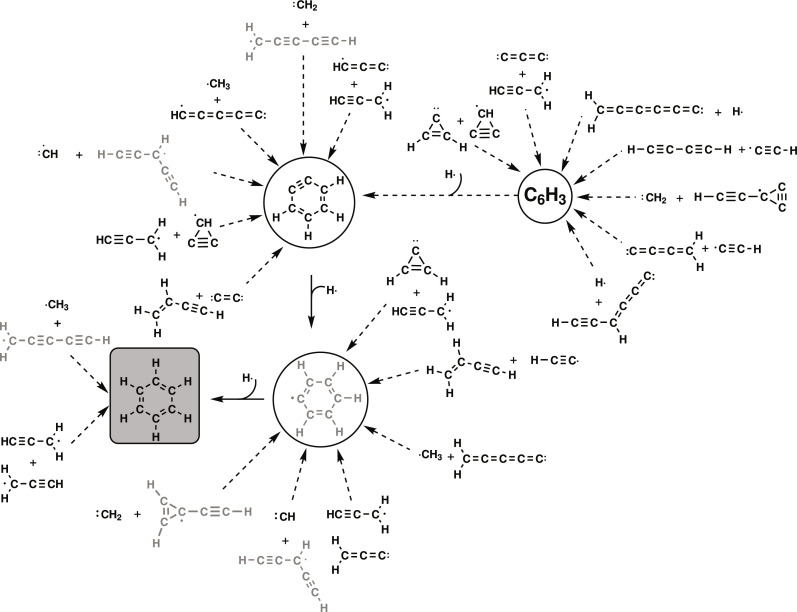
Summary scheme of different ISM-detected (in black) and undetected (in gray) species encountered by AutoMeKin as possible reactants that lead to benzene and other C_6_-membered rings. Dashed arrows indicate paths that involve several elementary steps.

We present an extensive analysis of both one-step and multistep reaction pathways for the A + B → C type of reactions, derived from a reaction network generated using automated methods. The reaction profiles initiate with the barrierless recombination of two reactive molecules, which, in the case of multistep reactions, pass through submerged transition states before culminating in the formation of a C_6_-membered ring hydrocarbon. These findings are summarized in Fig. 5.

With this prescription, we confirmed that benzene can be formed by associating two propargyl radicals. In addition, benzene can be reached by the reaction of H_2_C_5_H with the methyl radical or by the reaction of vinylidene (undetected) with vinylacetylene. The most favorable path for benzene formation is the barrierless reaction between the phenyl radical and atomic hydrogen.

The analysis of the reaction networks for dehydrogenated forms of benzene such as the phenyl radical, *o*-benzyne, or different cyclic C_6_H_3_ structures reveals that the formation of these rings is very involved and comprises a plethora of reactive species. Two encircled species in Fig. 5, which are the phenyl and the C_6_H_3_ radicals, have not yet been encountered in the ISM, but their existence has been hypothesized.

We found that several reactions start with different combinations of radical reactants such as propargyl, cylopropynil, methyl, or acetyl radicals. Other paths initiate with the combination of linear cumulenic carbon scaffolds (usually carbenes) plus a radical or a neutral molecule, depending on the multiplicity of the final product. Some paths consist of simple hydrogen atom additions to a ring; this provides a common ground for the formation of different C_6_ species. A molecule that is very abundant in the TMC-1 cloud is vinylacetylene, which, in our scheme, plays a crucial role in the formation of *o*-benzyne by the addition of C_2_ [in agreement with the calculations of Kaiser *et al.* (*13*)] and in the formation of the phenyl radical by the addition of the ethynyl radical (C_2_H). Another molecule that is ubiquitous in C_6_ rings formation is the propargyl radical, which appears in the formation of benzene, the phenyl radical, *o*-benzyne, and 1,2,3-tridehydrobenzene by reaction with another propargyl molecule, propadienylidene, C_3_H (cyclic and linear forms), and C_3_, respectively. This completes the scheme depicted in Fig. 5. Last, some pathways involve the reaction of up-to-date undetected species with C_6_H_3_ and C_5_H_3_ empirical formulas plus a small radical or carbene.

The recent discovery that some PAHs undergo rapid radiative relaxation adds significance to the recombination paths identified in this study. This finding may serve as a foundation for developing kinetic models that incorporate these types of reactions. The abundance of viable pathways identified by AutoMeKin demonstrates the effectiveness of automated methods in constructing reaction networks and uncovering new reaction pathways that may have relevance in the ISM and other environments.

## MATERIALS AND METHODS

The search for the stationary points (minima and transition states) was carried out with AutoMeKin (*28*). This program runs high-energy trajectories combined with an algorithm able to identify bond-breaking/forming events. Snapshots along the trajectories provide guess transition state structures that are further optimized at a low electronic structure level. For the low-level calculations, we used the PM7 semiempirical method (*59*) implemented in MOPAC (*60*). For each successfully obtained transition state, we follow the MEP, so the minima (reactants and products) associated with that particular transition state are also identified. In the event that the current transition state search algorithm fails to locate the appropriate saddle points, the nudged elastic band method (*33*) is used to validate the absence of saddle points for the identified barrierless pathways. This tool was implemented in AutoMeKin in 2022 (revision 1123). The pipeline starts with a search for bonds whose bond order is less than 1.5. The search is performed for every single minimum in the reaction network. It then runs a constrained Langevin dynamics simulation with external forces to break the selected bonds within each minimum energy structure. An elementary step is considered barrierless if it meets the following criteria: (i) It results in two fragments, (ii) the energy of the resulting fragments is below a specified threshold, and (iii) no alternative pathway with a barrier, connecting the same reactants and products, has been found.

For the reactions with a barrier, the low-level transition state structures are further optimized at a high level, and new MEP calculations are performed to generate a more accurate reaction network. All the stationary points were optimized at the M08HX functional (*61*) with the 6-31+G(d,p) basis set except the C_2_ plus vinylacetylene channel that used the B3LYP functional (*62*) and the 6-311G(d,p) basis set.

All the low-level and high-level optimization calculations were performed with MOPAC and Gaussian 16 (*63*), respectively. For each DFT optimized geometry, we have confirmed that the wavefunction was stable. High-level Hessian calculations were performed to check the nature of the stationary points. Accurate electronic energies were obtained by single-point calculations carried out at the F12-CCSD(T)/cc-pVTZ level over the DFT optimizations. The F12-CCSD(T) calculations were performed with MOLPRO (*64*). All reported relative energies include the DFT-calculated zero-point energy contribution to the F12-CCSD(T) electronic energy. A scaling factor of 0.972 was applied to the zero-point energies (*65*).

We have chosen M08HX because it has provided a good representation of the very challenging barrierless association of CF_2_ and the dissociation of C_2_F_4_ (*66*). In addition, it has been used by some of us in the study of the association reactions of OH with methanol (*67*) and methylamine (*68*), followed by the hydrogen abstraction reactions. In all cases, the combination F12-CCSD(T)//M08HX produced thermal rate constants in good agreement with experiment. An interesting alternative to these electronic structure calculations could be the junChS and junChS-F12 composite methods of Puzzarini and coworkers (*69*). The location of reaction paths inside the reaction network generated by AutoMeKin was performed with amk_tools (*32*, *70*).

The reaction networks for the different potential energy surfaces were built incorporating some restrictions. During the barrierless fragmentation of the molecule, AutoMeKin only examines the cleavage of a single bond with a bond order smaller than 1.5 (MOPAC calculates the bond order). Barrierless fragmentations that involve the cleavage of multiple bonds, as may be the case of a carbon atom or a C_2_ molecule dissociating from a three-membered ring, are not considered. Moreover, we included a spin restriction during the dissociation into fragments. In particular, the fragments with an even number of electrons have singlet spin states, so intersystem crossing to different electronic states (for instance, triplet states) is forbidden.

## Supplementary Materials

### This PDF file includes:

Supplementary Text Figs. S1 to S4 Tables S1 to S4 References
