# Supplementary material for: Comprehensive computational automated search of barrierless reactions leading to the formation of benzene and other C6-membered rings
Source: Sci Adv. 2024 Sep 11;10(37):eadq4077. doi: 10.1126/sciadv.adq4077 (PMC11389753; doi:10.1126/sciadv.adq4077)
Supplement: Supplementary file 1 — Supplementary Text Figs. S1 to S4 Tables S1 to S4 References [file sciadv.adq4077_sm.pdf]

Science Advances
AAAS

Supplementary Materials for

Comprehensive computational automated search of barrierless reactions leading to the formation of benzene and other C_6-membered rings

Marta Castiñeira Reis *et al.*

Corresponding author: Marta Castiñeira Reis, marta.castineira.reis@usc.es;
Antonio Fernández Ramos, qf.ramos@usc.es

Sci. Adv. **10**, eadq4077 (2024)
DOI: 10.1126/sciadv.adq4077

This PDF file includes:

Supplementary Text
Figs. S1 to S4
Tables S1 to S4
References

S1 General observations

The presented frequencies and zero-point vibrational energies are reported as obtained. However, the relative energies reported in the main text include the scaling of frequencies. The scaling factor used is 0.972 (*65*).

S2 C_5H_3: Abbreviated reaction network of the most stable isomers

The reaction network (see Fig. S1) shows that several paths lead to the three most stable C_5H_3 conformers. They are the 2,4-pentadiynyl-1 (**F-MIN1**), the 1,4-pentadiynyl-3 (**F-MIN2**), and the ethynyl cyclopropenylidene (**F-MIN3**) radicals.

The 2,4-pentadiynyl radical can be formed in just one step by a barrierless reaction of the ethynyl radical with propadienylidene, or of methylene with the butadiynyl radical. An additional step is required when formed from C_2 plus the propargyl radical or from H_2 plus pentadiynylidene (tentatively detected (*71*) and confirmed (*72*) by Cernicharo *et al.*) Other paths are also possible but they involve more reaction steps. The ethynyl cyclopropenylidene radical can be formed by reaction of atomic hydrogen with ethynyl cyclopropenylidene (**F-PR2**) or by an isomer of this species (**F-PR15**). The first of the two C_5H_2 isomers has been detected in the ISM (*3*) but not the latter. Additionally, it should be noticed these two reactive channels may also lead to the ethynyl cyclopropenylidene plus atomic hydrogen (**F-PR2**), because these two products are more stable than the initial reactants by 27.33 and 19.25 kcal mol^{-1}, respectively. In fact, they may be a possible source of the recently detected ethynyl cyclopropenylidene (*3*), as indicated in the electronic structure calculations carried out by Fortenberry (*73*) and by Fortenberry and coworkers (*74*). Finally, the 1,4-pentadiynyl-3 radical may be formed as a biproduct

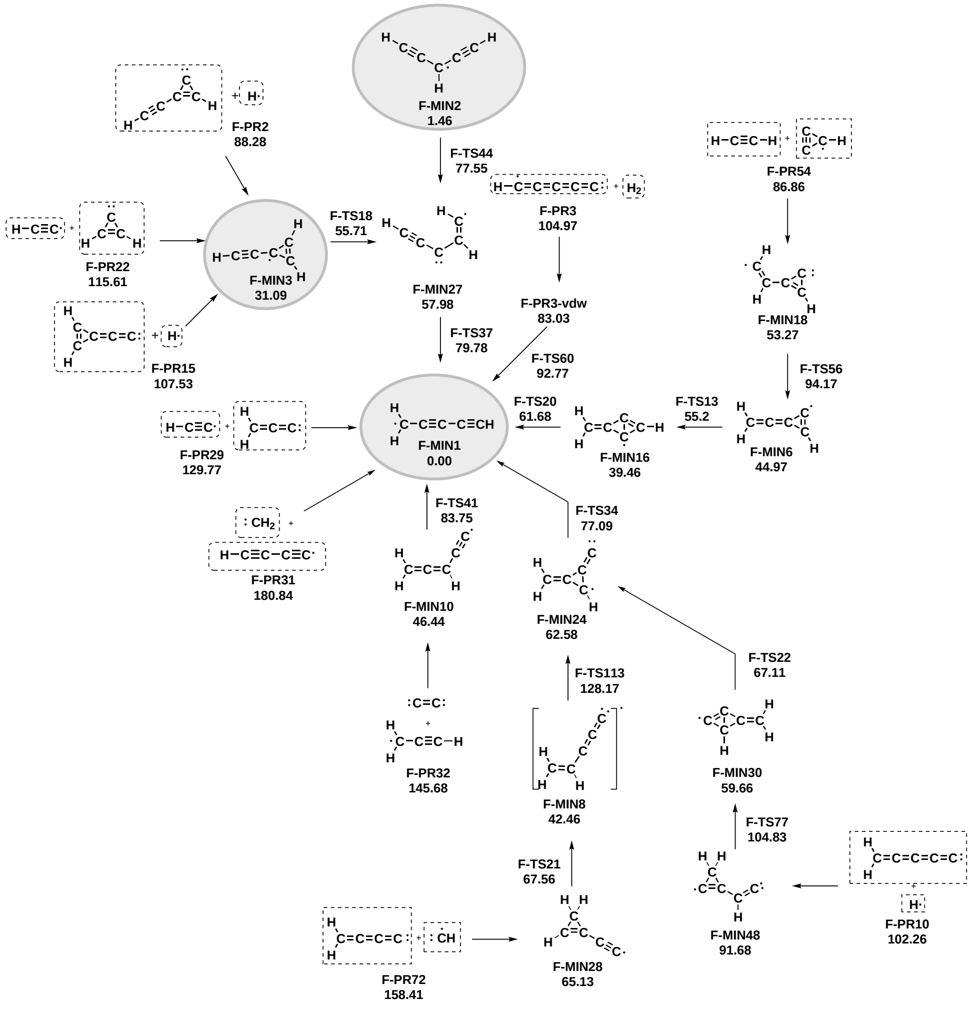

Figure S1: **Some of the paths that lead to the three most stable isomers of C_5H_3.**

of some of the previous reactions but the most favorable one is the recombination of hydrogen atom with HC_5H (not shown in Figure S1), although the latter has not been yet detected in the ISM.

S3 Propargyl radicals recombination

The reaction profiles depicted in Figure S2 show all the possibilities obtained by the automatic algorithm to form benzene in four or five elementary steps. For the former case, there is only

one reaction path (magenta profile in Figure S2) but for the latter, there are six alternative paths. With six elementary steps the number of possible paths increases to 36 and with eight steps is 455.

Different initial C_6H_6 aliphatic species are formed depending on whether the reaction involves the head (-CH_2) or the tail (-CCH) of each of the propargyl radicals. Thus, the bond-forming reaction through two tails leads to 1,2,4,5-hexatetraene (**MIN15**); it leads to 1,2-hexadiene-5-yne (**MIN26**) by assembling a tail and a head, and to 1,5-hexadiyne (**MIN30**) by reaction through the two heads. The intermediates **MIN15** and **MIN30** can isomerize through **TS69**, as it was also described by Miller and Klippenstein (*15*).

Another interesting intermediate is 1,3-hexadiene-5-yne (**MIN4**), which is formed in 2 steps either from **MIN15** or from **MIN26**. AutoMeKin finds a total of six reaction paths that lead to benzene from the propargyl radicals passing through **MIN4**, all of them in 6 elementary steps (the paths involving **MIN4** are not displayed in Figure S2). This intermediate can lead to benzene after three steps as described in the main text and also by Mebel *et al.* (*75*), although the routes from the two sources differ.

Fulvene (**MIN2**) is in most paths a transient species that can be formed from the propargyl radicals in just two steps by passing through **MIN26** or through **MIN30**. The shortest path to benzene (**MIN1**) is through fulvene that leads first to bicyclic-[3.1.0]-benzene (**MIN12**) and then to benzene through (**TS194**) (violet and brown profiles in Figure S2). However, this last elementary reaction is very high in energy, and the indirect rearrangement with a transition state that proceeds toward the metastable (**MIN41**) minima, which very easily yields benzene (orange profile in Figure S2) is more favorable. This last step is shared with the reaction that leads to **MIN84** and then to fulvene through **TS76**. It is clear that although fulvene has not yet been detected in the ISM, it shares many of the first elementary steps with benzene.

However, it is also possible to reach benzene without passing through fulvene, by forming

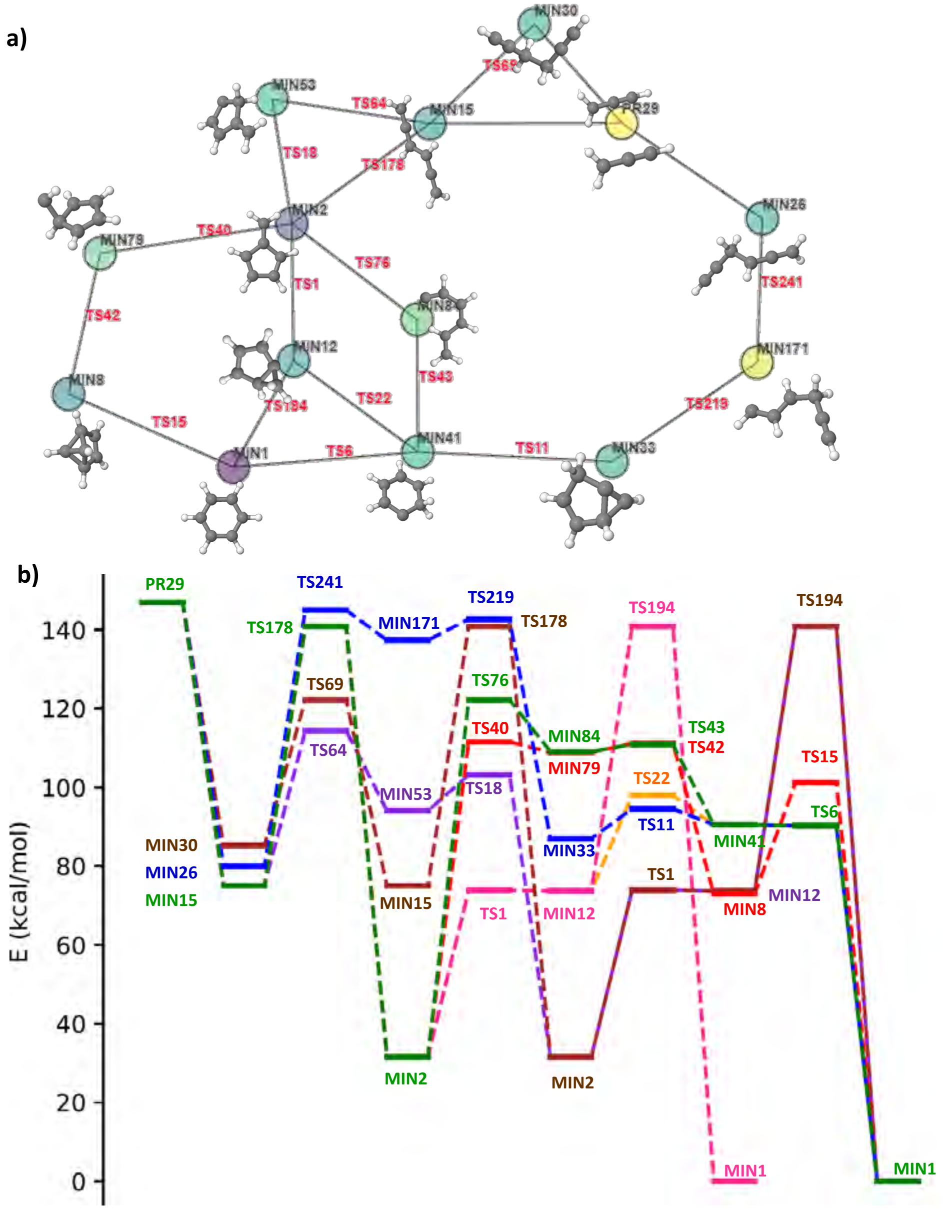

Figure S2: **Paths between benzene and the propargyl radicals.** a) Reaction network showing the link between benzene **MIN1** and the propargyl radicals **PR29** with up to 5 elementary steps. b) Reaction profile for benzene formation based on the above reaction network. Relative energies are also displayed.

MIN26, **MIN171**, and the bicyclic structures **MIN76**, and **MIN41** (green profile in Figure S2).

From **PR29** it is also possible to reach benzvalene **MIN8** (red profile in Figure S2), a high-

energy tricyclic isotope of benzene that was synthesized by (*76*).

S4 Formation of *o*-benzyne from H_2C_6H + H

Kaiser *et al.* (*13*) performed crossed molecular beam experiments and found that C_2 plus vinylacetylene can lead l-H_2C_6H plus the hydrogen atom at a collision energy of 31 kJ mol^{-1}. A reaction that is also possible by decomposition of ethynylbutatriene (**B-MIN7**, **si4**) or vinyldiacetylene (**B-MIN3**, **si5**) (see Figure S3). In particular, we have found up to 7 different paths considering l-H_2C_6H plus the hydrogen atom as the reactants (**B-PR109**).

Our calculations show that both the **B-MIN3** and **B-MIN7** intermediates can form from a barrierless recombination reaction. These reaction paths were also reported by (*13*) although these authors did not report any connectivity between **B-MIN3** and *o*-benzyne. AutoMeKin provides 5 paths that may lead to *o*-benzyne and that pass through the **B-MIN3** intermediate; one of them is a direct recombination of l-H_2C_6H and atomic hydrogen and two of them go through **B-MIN7**. The intermediate structures **B-MIN3** and **B-MIN7** can interconvert by hydrogen migrations and pass through a highly energetic carbene (**B-MIN171**). Notice that **B-MIN171** can also be obtained without going through these two minima. It involves a hydrogen atom addition with a subsequent rotation about the central C-C single bond to prepare the structure to form a six-member ring (**B-MIN106**).

Ethynylbutatriene can evolve to **B-MIN135** or **B-MIN11**. The barrier height to form **B-MIN4** from the two minima is very high and less than 3 kcal mol^{-1} below the reactants, so these paths are unlikely to reach *o*-benzyne. The same occurs with the **B-MIN17**→**B-MIN1**, which has a very high final barrier. However, there is still another path that goes through **B-MIN11**→**B-MIN74**→**B-MIN106** that presents lower reaction barriers. In the case of vinyldiacetylene, the reaction may occur by a six-membered ring cyclization forming **B-MIN98**, which by two successive hydrogen shift reactions forms *o*-benzyne, or by a five-membered ring

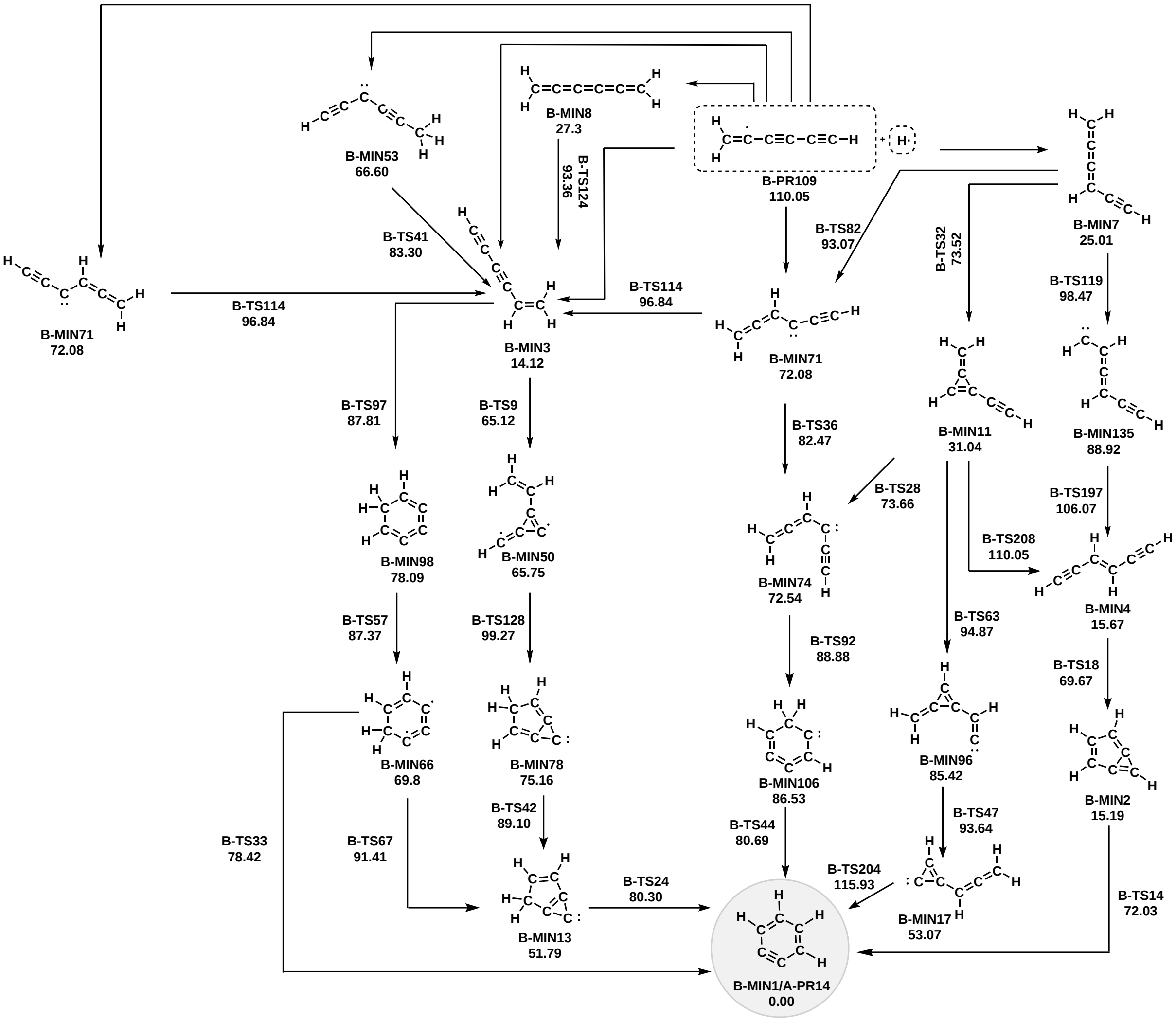

Figure S3: **Reactions involving l-H$_2$C$_6$H + H and that may lead to *o*-benzyne.**

cyclization (**B-MIN78**) that undergoes in the next step a H-shift reaction **B-MIN13** and a final C-C cleavage to form the six-membered ring.

S5 Potential reactants for the phenyl radical.

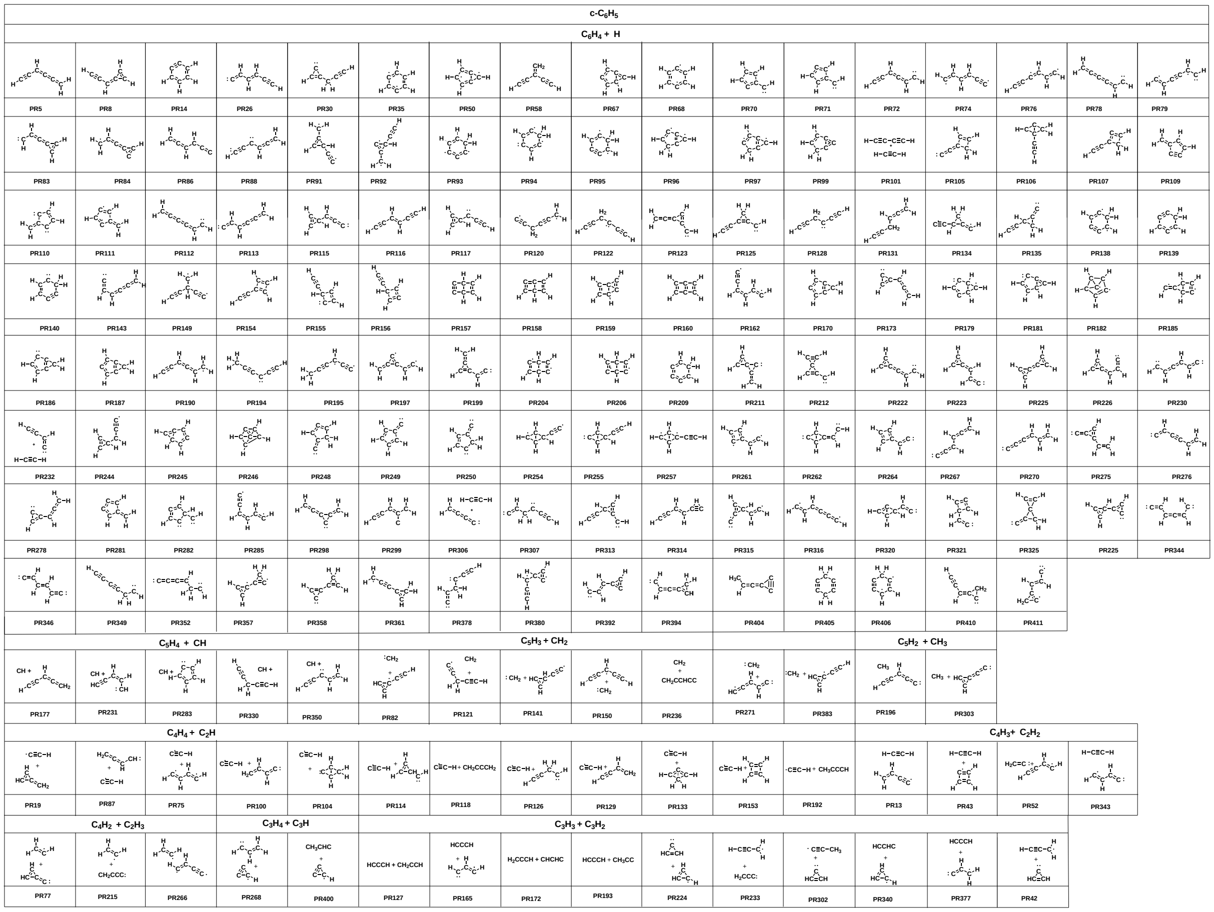

Figure S4: **List of all reactants found compatible with the formation of the phenyl radical in the ISM.**

S6 The most stable isomers found for the C6-derivatives

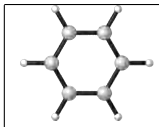	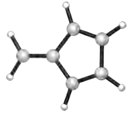	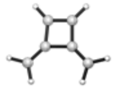	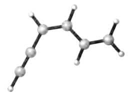	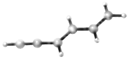
**MIN1**	**MIN2**	**MIN3**	**MIN4**	**MIN5**
0.0	34.09	63.59	66.45	66.5
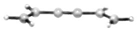	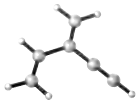	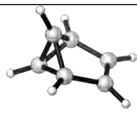	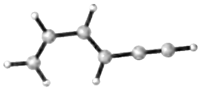	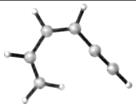
**MIN6**	**MIN7**	**MIN8**	**MIN9**	**MIN10**
66.56	68.15	68.55	69.3	69.77
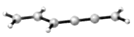	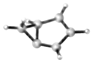	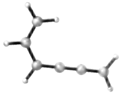	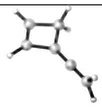	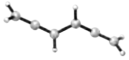
**MIN11**	**MIN12**	**MIN13**	**MIN14**	**MIN15**
72.5	73.26	74.42	75.19	75.33
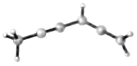	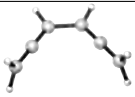	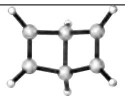	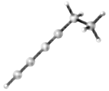	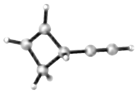
**MIN16**	**MIN17**	**MIN18**	**MIN19**	**MIN20**
75.48	77.54	77.77	78.1	79.73

Table S1: The 20 most stable isomers with C_6H_6 empirical formula.

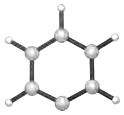	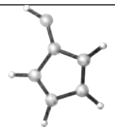	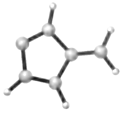	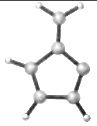	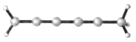
**A-MIN1**	**A-MIN2**	**A-MIN3**	**A-MIN4**	**A-MIN5**
0.0	33.07	37.78	39.32	45.68
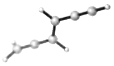	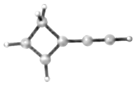	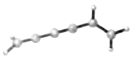	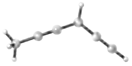	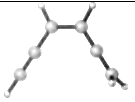
**A-MIN6**	**A-MIN7**	**A-MIN8**	**A-MIN9**	**A-MIN10**
48.78	48.86	48.98	49.22	49.57
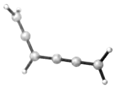	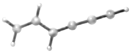	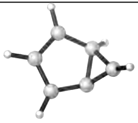	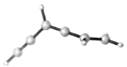	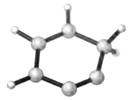
**A-MIN11**	**A-MIN12**	**A-MIN13**	**A-MIN14**	**A-MIN15**
50.57	55.34	56.03	56.96	57.94
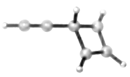	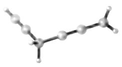	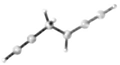	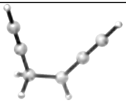	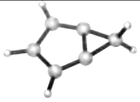
**A-MIN16**	**A-MIN17**	**A-MIN18**	**A-MIN19**	**A-MIN20**
58.86	60.64	61.37	62.13	62.26

Table S2: The 20 most stable isomers with C_6H_5 empirical formula.

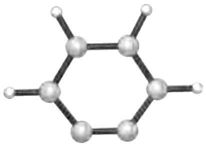	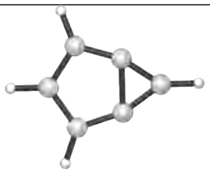	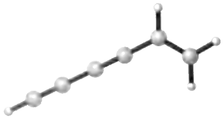	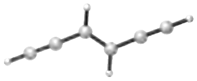	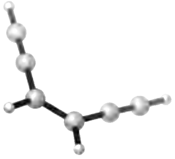
**B-MIN1**	**B-MIN2**	**B-MIN3**	**B-MIN4**	**B-MIN5**
0.0	6.75	13.48	15.73	16.24
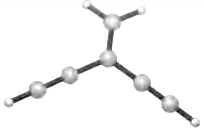	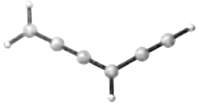	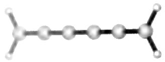	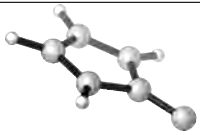	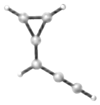
**B-MIN6**	**B-MIN7**	**B-MIN8**	**B-MIN9**	**B-MIN10**
19.61	22.9	23.42	26.57	31.02
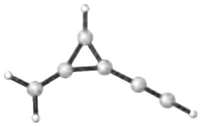	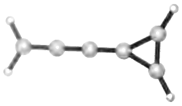	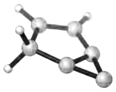	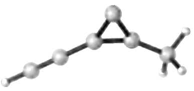	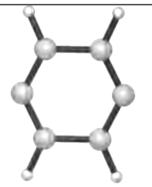
**B-MIN11**	**B-MIN12**	**B-MIN13**	**B-MIN14**	**B-MIN15**
32.44	38.17	44.22	44.35	44.51
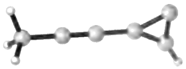	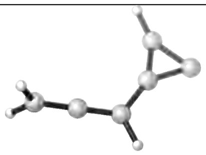	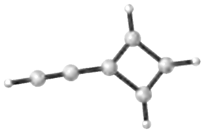	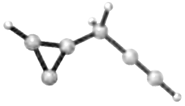	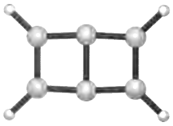
**B-MIN16**	**B-MIN17**	**B-MIN18**	**B-MIN19**	**B-MIN20**
44.62	45.05	46.59	53.14	55.72

Table S3: The 20 most stable isomers with C_6H_4 empirical formula.

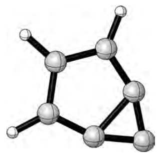	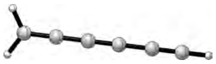	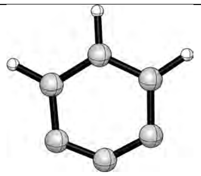	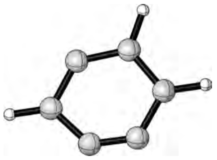	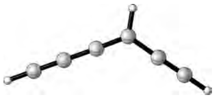
**C-MIN1**	**C-MIN2**	**C-MIN3**	**C-MIN4**	**C-MIN5**
0.0	7.36	8.84	10.84	17.26
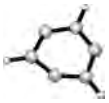	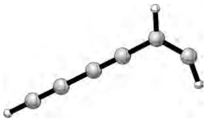	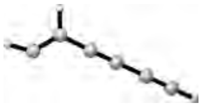	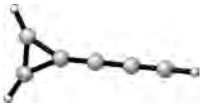	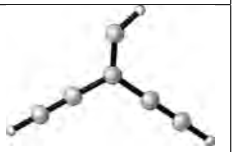
**C-MIN6**	**C-MIN7**	**C-MIN8**	**C-MIN9**	**C-MIN10**
24.68	24.74	24.74	31.06	32.35
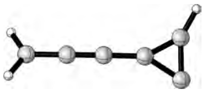	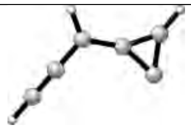	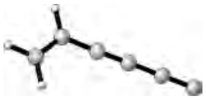	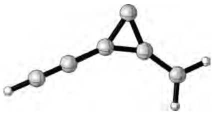	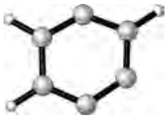
**C-MIN11**	**C-MIN12**	**C-MIN13**	**C-MIN14**	**C-MIN15**
32.47	34.23	36.71	37.32	38.78
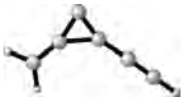	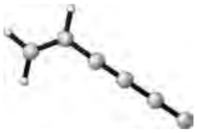	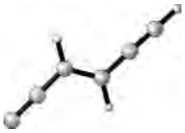	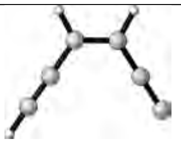	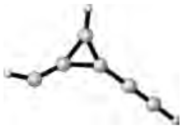
**C-MIN16**	**C-MIN17**	**C-MIN18**	**C-MIN19**	**C-MIN20**
39.76	39.84	40.48	41.79	42.37

Table S4: The 20 most stable isomers with C_6H_3 empirical formula.

S7 Absolute energies, Cartesian coordinates and frequencies of the stationary points reported in the main text

PR7frag1

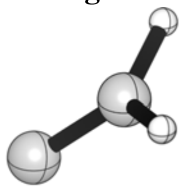

M08HX/6-31+G(d,p) = -77.245464

M08HX/6-31+G(d,p)+ZPVE = -77.22145

CCSD(T)-F12/cc-pVTZ-F12//M08HX/6-31+G(d,p) = -77.144687

Cartesian coordinates

C 0.000000 0.000000 -0.821579

C -0.000000 -0.000000 0.479304

H 0.000000 0.945005 1.026827

H 0.000000 -0.945005 1.026827

Frequencies

1740.8562

3153.8161

3242.6285

412.4233

771.3452

1219.6548

PR7frag2

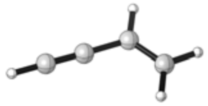

M08HX/6-31+G(d,p) = -154.697844

M08HX/6-31+G(d,p)+ZPVE = -154.636449

CCSD(T)-F12/cc-pVTZ-F12//M08HX/6-31+G(d,p) = -154.501632

Cartesian coordinates

C 1.656237 -0.380795 0.000021

C 0.646144 0.497141 0.000117

C -0.736438 0.117934 -0.000019

C -1.910708 -0.173382 -0.000127

H 2.689979 -0.038628 0.000133

H 0.851972 1.569773 0.000311

H -2.947230 -0.441505 -0.000223

H 1.473869 -1.455030 -0.000172

Frequencies

3171.2273

3263.9418

3483.3042

1701.2261

2248.0942

3161.2419

1105.7190

1311.8815

1430.6503

892.3775

967.4014

1007.2486

675.4151

691.9258

716.3175

225.7253

337.6865

557.9479

PR7-vdw

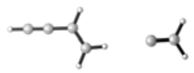

M08HX/6-31+G(d,p) = -231.945695

M08HX/6-31+G(d,p)+ZPVE = -231.8595

CCSD(T)-F12/cc-pVTZ-F12//M08HX/6-31+G(d,p) = -231.648547

Cartesian coordinates

C 1.487449 -0.159291 0.279110

C 2.782627 -0.053987 0.278332

H 3.403801 -0.951870 0.314243

H 3.253586 0.930893 0.241815

C -2.271459 -0.648347 0.186046

C -3.078613 0.417827 0.121916

C -4.509421 0.333606 0.068375

6 -5.718021 0.291025 0.021584

H -1.188739 -0.528371 0.224334

H -2.654443 1.424331 0.107503

H -6.786580 0.242830 -0.019597

H -2.680521 -1.658932 0.201336

Frequencies

3240.0602

3255.5428

3487.6521

3150.5003

3151.8248

3168.5325

1699.2872

1745.2100

2247.4689

1224.2790

1311.8554

1442.3334

986.2329

1018.3319

1109.3936

729.4808

781.7303

898.7598

563.6453

669.4714

694.9211

234.3885

354.4024

405.4324

59.2487

64.5588

75.3488

-40.3013

21.1152

44.2644

TS246

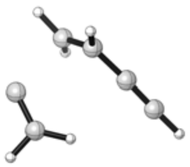

M08HX/6-31+G(d,p) = -231.950795

M08HX/6-31+G(d,p)+ZPVE = -231.861653

CCSD(T)-F12/cc-pVTZ-F12//M08HX/6-31+G(d,p) = -231.653157

Cartesian coordinates

C 1.3919030.055012 0.105314

C 0.394453 1.003739 0.468627

C -0.520911 1.484358 -0.405800

C -1.944717 -0.227728 0.020755

C 2.207123 -0.793575 -0.184379

C -1.383778 -1.414503 0.057197

H 0.357892 1.302934 1.517780

H -0.470892 1.238975 -1.466509

H -1.238515 2.239746 -0.096881

H -2.133234 -2.214604 0.040543

H -0.328261 -1.692600 0.088072

H 2.948572 -1.518273 -0.453287

Frequencies

3207.1170

3281.1637

3476.3433

3117.3080

3173.9139

3183.9522

1645.1117

1688.0593

2234.7915

1283.3192

1303.2136

1431.7738

980.8395

992.8054

1109.5838

764.6599

835.9698

904.1331

677.9821

685.4151

696.8668

410.9381

478.3093

562.4009

206.3230

228.6650

325.2091

-231.5532

112.6218

129.8004

MIN31

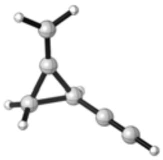

M08HX/6-31+G(d,p) = -232.056053

M08HX/6-31+G(d,p)+ZPVE = -231.960951

CCSD(T)-F12/cc-pVTZ-F12//M08HX/6-31+G(d,p) = -231.752629

Cartesian coordinates

C -1.4443280.089267 0.135023

C -0.175968 -0.423520 0.603056

C 0.694312 -1.285649 -0.342205

C 1.091455 0.072959 0.019859

C -2.505670 0.517332 -0.253168

C 1.927343 1.089588 -0.087669

H -0.153805 -0.711421 1.656468

H 0.299563 -1.460389 -1.343665

H 1.212921 -2.132095 0.109348

H 2.826854 1.014939 -0.699206

H 1.734980 2.030200 0.428296

H -3.443380 0.898910 -0.600615

Frequencies

3223.9263

3243.0619

3485.6019

3129.9165

3149.9973

3151.6724

1465.8050

1875.0902

2261.6246

1148.0614

1340.4291

1430.0182

1070.1218

1104.4354

1115.8688

937.3525

985.2709

1052.7800

757.4211

834.6736

919.2917

657.8927

674.0736

699.0610

342.9158

494.4131

547.2003

122.1677

199.9022

324.9530

TS132

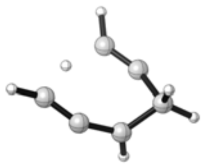

M08HX/6-31+G(d,p) = -231.971957

M08HX/6-31+G(d,p)+ZPVE = -231.882766

CCSD(T)-F12/cc-pVTZ-F12//M08HX/6-31+G(d,p) = -231.675079

Cartesian coordinates

C 0.299931 -1.085757 -0.370343

C 1.499200 -0.534052 0.350790

C 0.970290 0.770151 -0.044063

C 0.079510 1.666193 -0.160170

C -0.985109 -0.898427 0.021498

C -1.938606 -0.083271 0.163189

H 0.472311 -1.390365 -1.404261

H 2.452015 -0.836593 -0.091622

H 1.511501 -0.648964 1.440258

H 0.080514 2.744861 -0.060048

H -1.134026 1.089136 -0.215308

H -2.933608 0.032899 0.565577

Frequencies

3178.8120

3296.9480

3349.6385

2029.1203

3094.1233

3166.5504

1454.7453

1460.1959

1953.4061

1217.4273

1229.0183

1430.5158

1053.3583

1138.3630

1147.5960

904.6465

957.7438

980.7847

731.7922

828.2937

866.3677

546.3633

593.0447

685.2908

378.1426

430.1180

508.5448

-1360.7395

250.7006

288.7720

MIN26

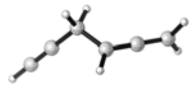

M08HX/6-31+G(d,p) = -232.059911

M08HX/6-31+G(d,p)+ZPVE = -231.965611

CCSD(T)-F12/cc-pVTZ-F12//M08HX/6-31+G(d,p) = -231.760165

Cartesian coordinates

C 0.483292 0.286057 0.612259

C -0.619387 0.783748 -0.304999

C -1.855417 0.006128 -0.151976

C -2.868804 -0.634985 -0.009254

C 1.665999 -0.075207 0.184931

C 2.847041 -0.436010 -0.247557

H 0.259383 0.230033 1.680070

H -0.280496 0.740739 -1.348952

H -0.836742 1.839709 -0.080327

H -3.766304 -1.205604 0.112716

H 3.048193 -1.469347 -0.531573

H 3.659623 0.286082 -0.332356

Frequencies

3169.3438

3233.6834

3482.9055

3042.6620

3102.6411

3147.7464

1483.0328

2094.6583

2261.9610

1300.5986

1374.9574

1459.8079

1040.0698

1148.2546

1217.5054

926.9408

969.9717

1020.9975

688.2670

877.1034

892.2687

555.6812

604.2844

680.0654

342.5538

365.0675

467.7164

59.5735

165.1704

217.3044

TS241

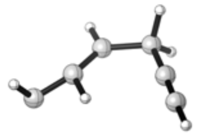

M08HX/6-31+G(d,p) = -231.951744

M08HX/6-31+G(d,p)+ZPVE = -231.862344

CCSD(T)-F12/cc-pVTZ-F12//M08HX/6-31+G(d,p) = -231.651884

Cartesian coordinates

C 2.019964 -1.238090 -0.010027

C -2.413476 -0.916069 -0.156297

C -1.336364 -0.079972 0.013653

C -0.660616 1.067162 -0.009769

C 1.511970 -0.141712 0.003567

C 0.850895 1.165781 0.020641

H 2.493210 -2.198629 -0.027675

H -2.731798 -1.330915 0.821988

H -0.756537 -1.083553 -0.001090

H -1.219509 2.001393 -0.033994

H 1.178797 1.718918 0.914989

H 1.201603 1.750183 -0.844819

Frequencies

3067.1217

3208.9683

3479.9499

2639.8431

3008.6202

3030.4741

1467.6587

1840.0630

2259.7213

1227.2269

1321.5304

1361.7521

982.8489

1052.6415

1201.6493

869.6298

914.9901

957.3174

701.6470

728.5470

833.8218

551.0424

568.9857

676.6147

300.9122

352.7148

357.8746

-304.0423

130.7416

147.0501

MIN171

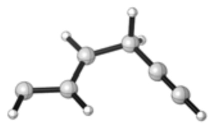

08HX/6-31+G(d,p) = -231.966818

M08HX/6-31+G(d,p)+ZPVE = -231.874643

CCSD(T)-F12/cc-pVTZ-F12//M08HX/6-31+G(d,p) = -231.66673778

Cartesian coordinates

C 2.395491 -0.966046 -0.003913

C -2.718564 -0.503222 0.020955

C -1.280332 -0.468340 -0.067798

C -0.734817 0.774357 -0.046631

C 1.637787 -0.027060 0.014990

C 0.723508 1.116766 0.037422

H 3.069233 -1.798315 -0.021029

H -3.014412 -1.549266 0.261623

H -0.613313 -1.340069 -0.066518

H -1.432612 1.617672 -0.062751

H 0.890542 1.709789 0.952207

H 0.962117 1.801460 -0.793678

Frequencies

3111.8990

3165.9868

3479.0411

2973.9706

3026.3345

3062.9273

1429.7048

1656.8267

2270.9008

1294.8656

1329.2983

1371.0748

1108.1390

1216.7342

1232.3503

973.7074

1021.5419

1025.2375

700.8787

814.5447

873.7259

466.7486

687.4366

689.9211

309.6254

347.2369

433.8892

100.4789

134.7368

150.2822

TS219

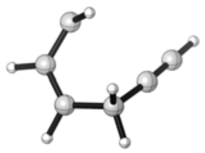

M08HX/6-31+G(d,p) = -231.957035

M08HX/6-31+G(d,p)+ZPVE = -231.957035

CCSD(T)-F12/cc-pVTZ-F12//M08HX/6-31+G(d,p) = -232.164387

Cartesian coordinates

C -1.587084 0.427686 -0.182389

C -1.081323 -0.826335 -0.268588

C 0.250510 -1.209602 0.351266

C 1.308082 -0.256633 0.001556

C -0.837305 1.556708 0.303923

C 2.109074 0.594799 -0.304587

H -2.508623 0.643829 -0.733476

H -1.590859 -1.576865 -0.876645

H 0.541376 -2.222559 0.044454

H 0.139506 -1.229709 1.450987

H 2.816783 1.350887 -0.576683

H -0.369905 1.314679 1.284278

Frequencies

3132.9302

3179.7267

3481.4253

2983.2952

3011.4925

3104.3057

1454.3491

1615.9744

2258.9709

1236.5727

1337.9737

1401.8105

1037.6602

1136.5974

1222.4398

923.4913

974.8668

1016.4306

691.9450

796.6364

901.8609

568.3819

575.7099

684.4074

325.5118

359.3308

487.1544

-93.7683

129.1661

266.6844

MIN33

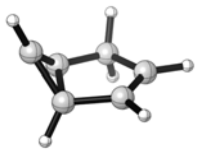

M08HX/6-31+G(d,p) = -232.057197

M08HX/6-31+G(d,p)+ZPVE = -231.959549

CCSD(T)-F12/cc-pVTZ-F12//M08HX/6-31+G(d,p) = -231.75232

Cartesian coordinates

C -1.594691 -0.283185 -0.423795

C -0.601948 -0.771360 0.266747

C 0.833650 -1.147718 0.137982

C 1.380341 0.245737 -0.229324

C -0.829700 0.709874 0.416628

C 0.487005 1.242801 -0.106901

H -2.228154 -0.397280 -1.297426

H 0.998050 -1.897453 -0.646690

H 1.302479 -1.509434 1.065918

H 2.421554 0.372208 -0.525304

H 0.719695 2.293749 -0.278312

H -1.261559 1.161317 1.313796

Frequencies

3195.8466

3219.5224

3257.6784

3048.6511

3113.3423

3126.4341

1461.3340

1650.1199

1827.9165

1260.1176

1296.9525

1341.4090

1108.9809

1137.7430

1183.3704

999.1829

1019.1925

1032.2896

941.9716

953.7443

976.9755

780.5344

823.0080

903.0475

673.1363

694.9487

724.8361

279.0787

334.5444

496.2981

TS11

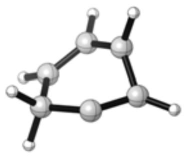

M08HX/6-31+G(d,p) = -232.035246

M08HX/6-31+G(d,p)+ZPVE = -231.939648

CCSD(T)-F12/cc-pVTZ-F12//M08HX/6-31+G(d,p) = -231.738349

Cartesian coordinates

C -0.422839 1.263451 -0.053957

C 0.928395 0.825484 0.276988

C 1.518840 -0.323684 -0.339435

C 0.547991 -1.122868 0.078240

C -1.378621 0.328340 -0.204907

C -0.927645 -1.058587 0.181403

H 1.466226 1.354317 1.072125

H 2.531362 -0.522942 -0.689018

H -1.341104 -1.839713 -0.477745

H -1.250336 -1.324404 1.200167

H -2.377358 0.527131 -0.595342

H -0.625505 2.332785 -0.140178

Frequencies

3178.8084

3185.5061

3201.6196

3035.1010

3078.1908

3122.2709

1414.9755

1664.8027

1712.9445

1284.6375

1352.0772

1391.0210

1152.5113

1188.2912

1262.9914

982.0027

1016.0952

1126.6243

884.2304

916.3685

947.4770

736.2363

785.9753

878.2920

519.4570

537.7918

734.0912

-374.9764

283.5077

388.8701

MIN41

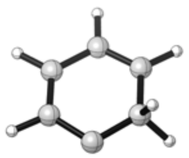

M08HX/6-31+G(d,p) = -232.049303

M08HX/6-31+G(d,p)+ZPVE = -231.953258

CCSD(T)-F12/cc-pVTZ-F12//M08HX/6-31+G(d,p) = -231.74519

Cartesian coordinates

C -1.280122 -0.684067 -0.002181

C -0.129955 -1.551329 -0.050472

C 1.149006 -0.791985 0.010690

C 1.201669 0.693919 -0.023869

C -1.201773 0.686730 0.015183

C 0.051450 1.403046 -0.005642

H -2.273690 -1.139557 -0.006558

H 1.907120 -1.247811 -0.648542

H 1.527221 -1.117259 1.009345

H 2.172018 1.196334 -0.040608

H 0.048463 2.492584 -0.012270

H -2.122778 1.277823 0.036389

Frequencies

3160.5769

3168.4364

3206.8473

2937.6430

3050.6102

3142.0977

1465.1354

1560.6320

1695.6968

1291.2568

1356.3485

1406.4117

1172.5280

1182.5603

1245.6059

1009.2013

1049.2436

1102.7551

969.9732

980.6879

1000.1539

754.4256

879.8134

883.9789

562.8641

591.9927

662.9960

92.5248

185.7602

389.8253

TS6

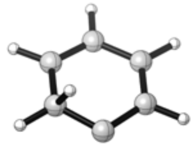

M08HX/6-31+G(d,p) = -232.047704

M08HX/6-31+G(d,p)+ZPVE = -231.952793

CCSD(T)-F12/cc-pVTZ-F12//M08HX/6-31+G(d,p) = -231.744463

Cartesian coordinates

C -1.235692 0.642707 -0.045313

C -1.117393 -0.813512 -0.000728

C 0.149796 -1.546290 -0.093618

C 1.277086 -0.633817 -0.024165

C -0.102106 1.393221 0.002327

C 1.169899 0.733796 0.028017

H -2.220888 1.108508 -0.102410

H -2.031801 -1.387346 -0.178035

H -0.800084 -1.138745 1.075050

H 2.278011 -1.070570 -0.062932

H 2.072318 1.349240 0.071069

H -0.147095 2.482277 -0.001854

Frequencies

3170.3852

3183.2199

3205.0054

2547.5584

3143.3726

3152.8314

1469.0244

1571.5090

1685.7204

1243.1983

1371.8133

1429.1979

1172.0482

1189.5683

1229.5814

1022.1235

1040.0385

1049.0994

928.0395

997.8994

999.2605

684.7431

839.8061

892.6072

577.5453

596.1455

653.6382

-495.0167

254.3674

361.6921

MIN1

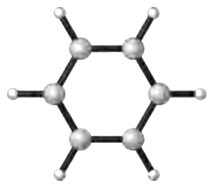

M08HX/6-31+G(d,p) = -232.197545

M08HX/6-31+G(d,p)+ZPVE = -232.09688

CCSD(T)-F12/cc-pVTZ-F12//M08HX/6-31+G(d,p) = -231.893881

Cartesian coordinates

C -0.698102 -1.209088 0.000017

C 0.698102 -1.209088 0.000021

C 1.396161 0.000000 -0.000004

C 0.698102 1.209088 -0.000034

C -1.396161 0.000000 -0.000012

C -0.698102 1.209088 -0.000038

H -1.243140 -2.153354 0.000037

H 1.243140 -2.153354 0.000044

H 2.486491 0.000000 -0.000001

H 1.243140 2.153354 -0.000054

H -1.243140 2.153354 -0.000061

H -2.48649 1 -0.000000 -0.000015

Frequencies

3202.7043

3202.7687

3212.0850

3178.4598

3188.2972

3188.4456

1518.9013

1674.6526

1675.4273

1346.1785

1365.0778

1518.2045

1159.8151

1191.8032

1194.0661

1030.4674

1068.8668

1069.3170

996.0142

1010.6771

1022.1095

867.5056

868.9986

994.4946

610.1325

686.8658

719.0252

407.0521

408.6781

610.1179

PR29frag1

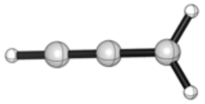

M08HX/6-31+G(d,p) = -115.969623

M08HX/6-31+G(d,p)+ZPVE = -115.928075

CCSD(T)-F12/cc-pVTZ-F12//M08HX/6-31+G(d,p) = -231.893881

Cartesian coordinates

C 0.000000 -0.000000 -0.118716

C -0.000000 0.000000 1.260382

C 0.000000 -0.000000 -1.343613

H -0.000000 -0.935082 1.812604

H 0.000000 0.935082 1.812604

H 0.000000 -0.000000 -2.413529

Frequencies

3181.8875

3285.2153

3475.0953

1077.5771

1452.7033

2033.9871

663.3096

694.8469

1030.8877

364.4946

421.9277

555.6391

PR29frag2

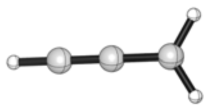

M08HX/6-31+G(d,p) = -115.969623

M08HX/6-31+G(d,p)+ZPVE = -115.928075

CCSD(T)-F12/cc-pVTZ-F12//M08HX/6-31+G(d,p) = -231.893881

Cartesian coordinates

C 0.000000 -0.000000 -0.118716

C -0.000000 0.000000 1.260382

C 0.000000 -0.000000 -1.343613

H -0.000000 -0.935082 1.812604

H 0.000000 0.935082 1.812604

H 0.000000 -0.000000 -2.413529

Frequencies

3181.8875

3285.2153

3475.0953

1077.5771

1452.7033

2033.9871

663.3096

694.8469

1030.8877

364.4946

421.9277

555.6391

MIN15

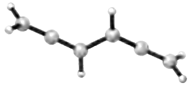

M08HX/6-31+G(d,p) = -232.070801

M08HX/6-31+G(d,p)+ZPVE = -231.977026

CCSD(T)-F12/cc-pVTZ-F12//M08HX/6-31+G(d,p) = -231.767567

Cartesian coordinates

C -0.612459 1.720717 0.000000

C 0.614188 -0.405597 -0.000000

C 0.612459 -1.720717 0.000000

C -0.614188 0.405597 -0.000000

C -0.612459 3.027630 0.000000

C 0.612459 -3.027630 0.000000

H -0.612719 3.591242 -0.934279

H 1.570337 0.122527 -0.000000

H 0.612719 -3.591242 -0.934279

H 0.612719 -3.591242 0.934279

H -1.570337 -0.122527 -0.000000

H -0.612719 3.591242 0.934279

Frequencies

3171.8310

3221.7941

3221.8047

3137.8576

3138.2674

3164.1873

1512.1368

2066.7838

2083.6754

1260.4281

1387.4978

1449.8704

1052.9769

1117.4732

1172.9841

914.8834

1010.3516

1012.0433

890.7052

892.4100

893.1538

544.8923

555.0632

675.2608

329.4287

362.6931

484.1708

80.4630

121.8197

235.8280

TS178

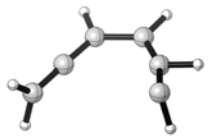

M08HX/6-31+G(d,p) = -231.96043

M08HX/6-31+G(d,p)+ZPVE = -231.871907

CCSD(T)-F12/cc-pVTZ-F12//M08HX/6-31+G(d,p) = -231.657651

Cartesian coordinates

C 1.515210 -0.080766 -0.043459

C 1.721992 -1.400122 0.187035

C -2.183010 -0.876089 -0.018685

C -1.383745 0.158880 0.013411

C 0.897841 1.105771 -0.034884

C -0.568582 1.192811 0.028344

H 2.684575 -0.205008 0.001977

H 1.908867 -1.970257 -0.743306

H -2.586626 -1.249587 -0.962189

H -2.475791 -1.393686 0.896629

H -1.003593 2.191309 0.107175

H 1.474334 2.024321 -0.090852

Frequencies

3170.2109

3209.5136

3238.2971

2561.6305

3023.8498

3127.7028

1479.6913

1829.8979

2059.4094

1167.5387

1361.7351

1410.2230

993.6267

1015.9000

1076.8166

903.0089

909.6243

920.0223

785.4205

854.5426

898.6291

439.1092

559.0206

642.6649

271.2110

327.5821

418.2406

-544.4112

94.9599

107.2122

MIN2

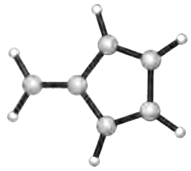

M08HX/6-31+G(d,p) = -232.140897

M08HX/6-31+G(d,p)+ZPVE = -232.042615

CCSD(T)-F12/cc-pVTZ-F12//M08HX/6-31+G(d,p) = -231.841269

Cartesian coordinates

C -0.000000 -1.180897 0.126417

C -0.000000 -0.000000 -0.761194

C -0.000000 -0.000000 -2.103233

C -0.000000 1.180897 0.126417

C 0.000000 -0.739222 1.404015

C -0.000000 0.739222 1.404015

H -0.000000 -2.211221 -0.219329

H -0.000000 -0.932008 -2.669814

H 0.000000 0.932008 -2.669814

H -0.000000 2.211221 -0.219329

H 0.000000 1.356187 2.299828

H -0.000000 -1.356187 2.299828

Frequencies

3244.6307

3250.0907

3255.3943

3149.7151

3221.7425

3231.5132

1571.6244

1652.9878

1739.2179

1357.1802

1373.7754

1447.8145

1099.6834

1104.5353

1254.7497

958.3997

974.0893

1014.1205

916.2427

949.8650

956.8408

790.6873

794.9944

803.3291

626.6983

676.7502

699.3814

201.1142

331.8203

491.9726

TS40

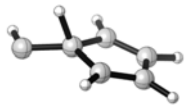

M08HX/6-31+G(d,p) = -232.01111

M08HX/6-31+G(d,p)+ZPVE = -231.918152

CCSD(T)-F12/cc-pVTZ-F12//M08HX/6-31+G(d,p) = -231.708677

Cartesian coordinates

C -2.193483 -0.169263 -0.232816

C 0.154743 -1.194392 0.050099

C 1.402018 -0.703158 -0.060323

C 1.350848 0.776682 -0.054693

C -0.803832 -0.038439 0.113504

C 0.073862 1.184721 0.060595

H -2.597496 0.868551 -0.243198

H -0.167225 -2.231815 0.061559

H 2.316716 -1.284052 -0.155007

H 2.224065 1.420228 -0.142187

H -0.302751 2.204169 0.090184

H -1.378243 -0.113984 1.130450

Frequencies

3230.0706

3248.3837

3262.6661

2551.4961

2972.5723

3218.2419

1422.9910

1599.7780

1677.6675

1253.0830

1327.1204

1344.0277

1060.4139

1093.4435

1103.9672

966.8520

979.3695

1007.6853

854.2435

891.6364

960.7031

722.1408

792.1796

819.4835

504.5692

614.8003

701.2433

-174.4972

269.6631

353.2746

MIN79

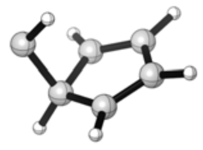

M08HX/6-31+G(d,p) = -232.017296

M08HX/6-31+G(d,p)+ZPVE = -231.921618

CCSD(T)-F12/cc-pVTZ-F12//M08HX/6-31+G(d,p) = -231.715569

Cartesian coordinates

C -0.326937 1.054004 -0.330285

C -1.694305 -0.069741 0.709654

C -0.846019 -0.355734 -0.494655

C 0.341471 -1.198421 -0.187015

C 0.974838 0.976192 0.077737

C 1.390356 -0.408292 0.171653

H -0.901288 1.955202 -0.518204

H -1.005620 -0.189884 1.576049

H -1.410574 -0.555403 -1.411869

H 0.309035 -2.285384 -0.151309

H 2.358710 -0.745673 0.533363

H 1.613311 1.833097 0.289433

Frequencies

3226.3915

3246.5110

3271.5835

2984.7059

3120.7058

3210.6742

1426.1327

1502.7976

1623.7710

1230.5833

1280.7186

1336.3932

1077.7621

1113.4749

1129.4616

981.2981

1019.3832

1064.2165

896.0721

953.7782

971.1645

771.1508

819.4426

821.1819

535.7248

727.4220

756.6597

176.9790

257.9957

463.4685

TS42

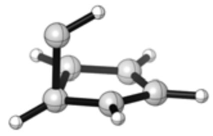

M08HX/6-31+G(d,p) = -232.012825

M08HX/6-31+G(d,p)+ZPVE = -231.917669

CCSD(T)-F12/cc-pVTZ-F12//M08HX/6-31+G(d,p) = -231.711379

Cartesian coordinates

C -0.034935 -1.158486 -0.164439

C 1.275288 -0.577135 0.033946

C 1.129034 0.781040 -0.014729

C -0.275919 1.075202 -0.223835

C -0.959445 -0.152375 -0.596854

C -1.264793 0.045169 0.896852

H -0.282766 -2.215584 -0.108058

H 2.168286 -1.134224 0.300268

H 1.893897 1.526509 0.186287

H -0.703793 2.063682 -0.358748

H -0.454941 -0.048529 1.623840

H -1.836063 -0.272345 -1.229228

Frequencies

3245.6431

3256.2880

3265.9113

3197.7581

3208.8380

3236.5780

1419.4452

1429.4389

1564.7477

1169.4759

1271.6967

1392.2054

1064.9167

1089.6097

1109.3374

980.2181

1030.2664

1058.0095

872.5626

897.0225

926.2898

789.9314

833.2583

848.4812

577.8539

711.2031

746.5328

-273.8440

157.8104

417.5928

MIN8

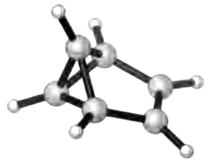

M08HX/6-31+G(d,p) = -232.086174

M08HX/6-31+G(d,p)+ZPVE = -231.987707

CCSD(T)-F12/cc-pVTZ-F12//M08HX/6-31+G(d,p) = -231.775615

Cartesian coordinates

C -0.000000 1.071135 0.205954

C -0.000000 0.669337 -1.251937

C 0.000000 -0.669337 -1.251937

C 0.000000 -1.071135 0.205954

C 0.724175 0.000000 1.007809

C -0.724175 0.000000 1.007809

H -0.000000 2.105267 0.546105

H -0.000000 1.351088 -2.097603

H 0.000000 -1.351088 -2.097603

H 0.000000 -2.105267 0.546105

H -1.486195 0.000000 1.780544

H 1.486195 -0.000000 1.780544

Frequencies

3236.1018

3249.8580

3261.9321

3209.9592

3210.5169

3231.8135

1347.1412

1438.3427

1650.8482

1209.7275

1225.3933

1287.4682

1120.1888

1123.7774

1135.3809

994.0232

995.1048

1003.7585

906.7347

932.0629

938.7731

818.6957

828.5613

864.0619

701.8728

777.3507

791.6878

514.3969

546.7940

669.9459

TS15

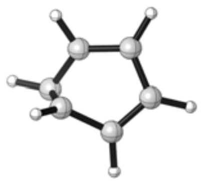

M08HX/6-31+G(d,p) = -232.026442

M08HX/6-31+G(d,p)+ZPVE = -231.931264

CCSD(T)-F12/cc-pVTZ-F12//M08HX/6-31+G(d,p) = -231.72736

Cartesian coordinates

C -0.822320 -0.623550 0.516860

C -1.407093 -0.049716 -0.607035

C -0.515098 0.987288 0.052983

C 0.891048 1.041718 0.072116

C 0.468436 -1.185813 -0.005254

C 1.452699 -0.239117 -0.132095

H -1.251967 -0.883757 1.494694

H -2.477394 0.143195 -0.709402

H -1.094230 1.831072 0.434146

H 1.440059 1.969746 0.215153

H 2.467646 -0.425596 -0.476703

H 0.509857 -2.219523 -0.343339

Frequencies

3218.6288

3231.2827

3243.7458

3070.8976

3159.0850

3173.8828

1466.4790

1492.9387

1559.9438

1262.4103

1284.6183

1396.4448

1110.8382

1121.2393

1135.1981

975.6866

1015.5303

1091.6868

905.4649

914.9331

934.4424

747.7430

813.6571

818.5410

496.0159

687.7551

712.8772

-375.6860

336.9236

399.4423

PR32frag1

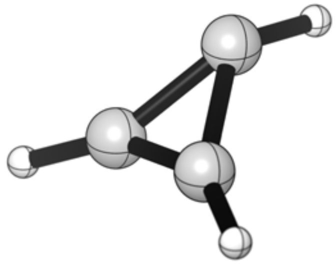

M08HX/6-31+G(d,p) = -232.026442

M08HX/6-31+G(d,p)+ZPVE = -115.883532

CCSD(T)-F12/cc-pVTZ-F12//M08HX/6-31+G(d,p) = -231.72736

Cartesian coordinates

C 0.875974 0.000028 -0.137292

C -0.419397 -0.657559 0.013563

C -0.419438 0.657536 0.013571

H -0.983532 -1.580487 0.066553

H 1.744353 0.000047 0.527859

H -0.983656 1.580414 0.066535

Frequencies

3108.7180

3263.8679

3311.0957

1048.1224

1262.4303

1698.5170

918.4182

975.7923

990.2090

617.8492

772.5042

887.9196

PR32frag2

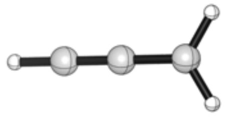

M08HX/6-31+G(d,p) = -232.026442

M08HX/6-31+G(d,p)+ZPVE = -115.928075

CCSD(T)-F12/cc-pVTZ-F12//M08HX/6-31+G(d,p) = -231.72736

Cartesian coordinates

C 0.000000 -0.000000 -0.118716

C -0.000000 0.000000 1.260382

C 0.000000 -0.000000 -1.343613

H -0.000000 -0.935082 1.812604

H 0.000000 0.935082 1.812604

H 0.000000 -0.000000 -2.413529

Frequencies

3181.8875

3285.2153

3475.0953

1077.5771

1452.7033

2033.9871

663.3096

694.8469

1030.8877

364.4946

421.9277

555.6391

MIN63

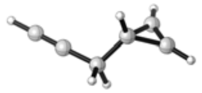

M08HX/6-31+G(d,p) = -232.031627

M08HX/6-31+G(d,p)+ZPVE = -231.93721

CCSD(T)-F12/cc-pVTZ-F12//M08HX/6-31+G(d,p) = -231.728125

Cartesian coordinates

C -2.691719 0.452550 0.048965

C -1.647666 -0.140425 -0.087840

C 1.575872 0.947345 -0.191595

C 2.184058 -0.186187 -0.016895

C -0.365964 -0.840475 -0.233235

C 0.771346 -0.119495 0.494121

H -3.618464 0.975030 0.166717

H -0.472668 -1.865404 0.155596

H 1.582789 1.968625 -0.550538

H 3.072058 -0.796244 -0.125965

H 0.597041 -0.034608 1.574730

H -0.116315 -0.927274 -1.301667

Frequencies

3271.6884

3319.5150

3483.7868

3034.3120

3074.6171

3090.5665

1462.0472

1754.4884

2256.8996

1254.1347

1309.9093

1402.9213

1045.2176

1080.1918

1206.3089

948.1584

973.7636

1038.8835

822.5817

876.8755

893.0110

634.6962

672.8668

680.9567

361.5378

394.0725

530.1352

64.5644

154.4314

351.4135

TS199

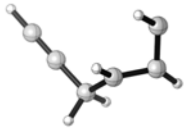

M08HX/6-31+G(d,p) = -231.959297

M08HX/6-31+G(d,p)+ZPVE = -231.867307

CCSD(T)-F12/cc-pVTZ-F12//M08HX/6-31+G(d,p) = -231.668366

Cartesian coordinates

C -0.542791 -1.041870 -0.016540

C -1.626239 -0.052657 -0.071682

C 0.737534 -0.428659 0.503482

C 1.862542 -0.220987 -0.282182

C -2.494943 0.785230 -0.101480

C 2.027921 1.136168 -0.148478

H -0.382627 -1.472341 -1.014952

H -0.841855 -1.863438 0.655123

H 2.124701 -0.799280 -1.171080

H 1.876596 1.582947 0.848083

H -3.266272 1.527003 -0.136788

H 0.705311 -0.038240 1.520897

Frequencies

3162.5438

3204.3557

3485.3066

3028.7747

3081.6886

3097.0140

1448.4940

1521.6194

2264.3664

1290.1663

1310.9851

1443.9454

1063.6703

1196.5816

1235.5817

912.6647

972.2155

983.2253

705.2462

853.7142

872.1296

550.0068

676.1037

686.7772

338.9829

361.0327

437.4500

-243.6616

39.5060

154.4273

PR1frag1

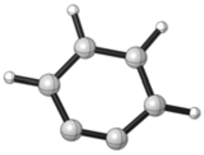

M08HX/6-31+G(d,p) = -230.85576

M08HX/6-31+G(d,p)+ZPVE = -230.780071

CCSD(T)-F12/cc-pVTZ-F12//M08HX/6-31+G(d,p) = -230.564185

Cartesian coordinates

C 0.000000 1.267717 -0.000000

C 1.171362 0.484682 0.000000

C 1.144164 -0.924865 0.000000

C -0.167125 -1.371979 0.000000

C -1.292114 0.703599 -0.000000

C -1.203698 -0.678885 -0.000000

H 0.096482 2.354534 -0.000000

H 2.138731 0.989303 0.000000

H 2.047237 -1.531670 0.000000

H -2.197980 1.306216 -0.000000

Frequencies

3197.6527

3221.0125

3224.4849

1511.3480

2062.7828

3182.9086

1324.8565

1444.8703

1497.8930

1115.9508

1158.0549

1271.3902

984.7419

1011.2052

1089.5075

840.0412

879.9493

939.1796

611.0695

617.4796

755.0418

390.8301

430.2818

461.1099

PR1frag2

M08HX/6-31+G(d,p) = -1.173503

M08HX/6-31+G(d,p)+ZPVE = -1.16333

CCSD(T)-F12/cc-pVTZ-F12//M08HX/6-31+G(d,p) = -1.174044

Cartesian coordinates

H -0.000000 -0.000000 0.371363

H 0.000000 0.000000 -0.371363

Frequencies

4465.6576

TS77

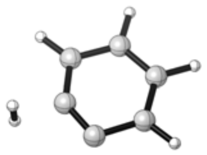

M08HX/6-31+G(d,p) = -231.990762

M08HX/6-31+G(d,p)+ZPVE = -231.900436

CCSD(T)-F12/cc-pVTZ-F12//M08HX/6-31+G(d,p) = -231.689094

Cartesian coordinates

C 0.285085 1.355061 -0.000008

C 1.335379 0.412675 -0.000006

C 1.087333 -0.961441 0.000012

C -0.237897 -1.479408 -0.000006

C -1.032745 0.910612 0.000016

C -1.098579 -0.479905 0.000003

H 0.499234 2.423164 -0.000030

H 2.365197 0.776200 -0.000019

H 1.942538 -1.643110 0.000023

H -2.471822 -0.839698 0.410608

H -2.471818 -0.839637 -0.410698

H -1.894781 1.577514 0.000056

Frequencies

3195.0494

3211.5729

3454.7351

1719.3649

3144.2903

3178.3521

1429.7605

1488.3041

1591.2083

1162.2154

1266.9203

1368.4442

1048.9704

1131.0701

1154.0567

955.8546

1015.5249

1017.7790

780.9586

885.3253

913.0543

663.2614

733.1785

758.6305

476.6628

531.1729

646.7189

-1063.8567

321.5188

404.4562

PR25frag1

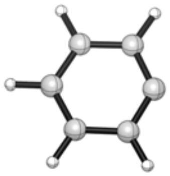

M08HX/6-31+G(d,p) = -231.990762

M08HX/6-31+G(d,p)+ZPVE = -231.418028

CCSD(T)-F12/cc-pVTZ-F12//M08HX/6-31+G(d,p) = -231.689094

Cartesian coordinates

C 0.000000 0.000000 1.397853

C 0.000000 1.226652 0.771096

C 0.000000 1.213827 -0.631645

C -0.000000 0.000000 -1.323479

C -0.000000 -1.226652 0.771096

C -0.000000 -1.213827 -0.631645

H 0.000000 2.163810 1.327439

H 0.000000 2.156464 -1.180621

H -0.000000 0.000000 -2.413295

H -0.000000 -2.156464 -1.180621

H -0.000000 -2.163810 1.327439

Frequencies

3199.4666

3201.2583

3209.8151

1671.6233

3179.8913

3186.4063

1469.7287

1482.0763

1615.0366

1170.4577

1299.5770

1331.6157

1058.7664

1079.3082

1166.7015

978.0895

1002.7999

1033.5288

825.9562

898.5965

975.9445

607.5861

672.5083

720.4719

396.4599

422.5562

589.8075

PR25frag2

M08HX/6-31+G(d,p)+ZPVE = -0.499294

CCSD(T)-F12/cc-pVTZ-F12//M08HX/6-31+G(d,p) = -231.689094

Cartesian coordinates

H 0.000000 0.000000 0.000000

PR28frag1

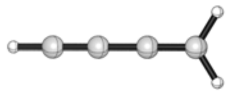

M08HX/6-31+G(d,p) = -231.990762

M08HX/6-31+G(d,p)+ZPVE = -153.978275

CCSD(T)-F12/cc-pVTZ-F12//M08HX/6-31+G(d,p) = -231.689094

Cartesian coordinates

C 0.000000 0.000000 0.569462

C 0.000000 0.000000 1.876843

C -0.000000 -0.000000 -0.759799

C -0.000000 -0.000000 -1.993088

H -0.000000 0.931828 2.451240

H 0.000000 -0.931828 2.451240

H -0.000000 -0.000000 -3.062987

Frequencies

3089.4057

3164.1770

3472.6834

1422.8712

1836.4827

2013.3114

903.5277

905.9029

969.3605

500.9576

617.2419

719.1471

123.7147

246.8425

414.7616

PR28frag2

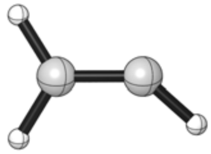

M08HX/6-31+G(d,p) = -231.990762

M08HX/6-31+G(d,p)+ZPVE = -77.844393

CCSD(T)-F12/cc-pVTZ-F12//M08HX/6-31+G(d,p) = -231.689094

Cartesian coordinates

C -0.048458 -0.588744 0.000000

C -0.048458 0.721900 -0.000000

H 0.674795 1.530686 -0.000000

H -0.977842 -1.163351 0.000000

H 0.884538 -1.166268 0.000000

Frequencies

3081.2834

3180.4500

3265.1302

1047.7922

1383.5158

1678.4607

707.8018

844.5855

928.9269

MIN140

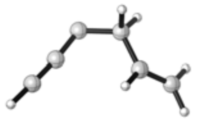

M08HX/6-31+G(d,p) = -231.977609

M08HX/6-31+G(d,p)+ZPVE = -231.886076

CCSD(T)-F12/cc-pVTZ-F12//M08HX/6-31+G(d,p) = -231.67828

Cartesian coordinates

C 1.193060 -0.381530 0.302259

C 2.369200 -0.712058 -0.229884

C -2.481548 -0.856520 -0.020994

C -1.651245 0.040671 -0.103826

C 0.584703 0.990656 0.184005

C -0.822128 1.137785 -0.264563

H 0.603172 -1.130702 0.838734

H 2.774592 -1.719138 -0.137125

H 2.968050 0.017001 -0.780327

H -3.225455 -1.624376 0.041392

H 0.486767 1.455563 1.193648

H 1.240626 1.687635 -0.358313

Frequencies

3159.5148

3241.1140

3470.4369

2930.0510

3075.1145

3143.2300

1448.2006

1731.9046

2075.4837

1298.7245

1327.0554

1384.1770

1063.3922

1160.2079

1250.8816

947.0596

960.4274

1017.9101

720.7124

832.0388

860.9912

427.2919

538.2113

681.2298

321.2841

340.7390

413.7189

69.6301

135.1676

152.2568

TS147

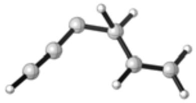

M08HX/6-31+G(d,p) = -231.96882

M08HX/6-31+G(d,p)+ZPVE = -231.879262

CCSD(T)-F12/cc-pVTZ-F12//M08HX/6-31+G(d,p) = -231.669517

Cartesian coordinates

C 1.205244 -0.452649 0.036071

C 2.531581 -0.590277 -0.030406

C -2.537818 -0.836356 0.003499

C -1.696616 0.042780 -0.026813

C 0.553372 0.879300 -0.003131

C -0.826795 1.158159 -0.128714

H 0.545758 -1.321723 0.096920

H 3.005431 -1.571268 -0.028734

H 3.189100 0.279527 -0.096696

H -3.293076 -1.594652 0.030055

H -0.056739 1.271680 1.015468

H 1.235723 1.730691 -0.120054

Frequencies

3175.9333

3243.7183

3482.8376

2218.5254

3110.0175

3144.4364

1481.2976

1721.4461

2167.5523

1301.9805

1366.1153

1409.7305

1028.7297

1169.2050

1255.8181

874.4124

954.8047

984.3355

700.3754

758.7948

837.2208

484.1144

529.0514

676.0801

247.4679

314.9015

436.2355

-763.7623

106.7171

129.4955

MIN4

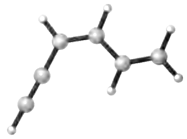

M08HX/6-31+G(d,p) = -232.086444

M08HX/6-31+G(d,p)+ZPVE = -231.99114

CCSD(T)-F12/cc-pVTZ-F12//M08HX/6-31+G(d,p) = -231.789565

Cartesian coordinates

C 1.266297 0.329739 0.000000

C 2.457172 0.944272 0.000000

C -1.626768 -2.137692 -0.000000

C -1.424516 -0.943834 -0.000000

C 0.000000 1.053507 -0.000000

C -1.216857 0.471127 -0.000000

H 1.210574 -0.762060 0.000000

H 3.389303 0.381244 0.000000

H 2.532789 2.033569 0.000000

H -1.802581 -3.193825 -0.000000

H -2.112922 1.093170 -0.000000

H 0.050866 2.145188 -0.000000

Frequencies

3191.3328

3249.1709

3485.7809

3149.0382

3163.6300

3170.8413

1666.3844

1729.4748

2242.3567

1306.0903

1385.3757

1459.5857

1036.2189

1163.7887

1253.7035

954.8067

982.7108

994.6195

711.5216

814.2462

903.9389

661.3230

698.8605

700.0908

305.8038

429.7032

486.7699

130.3094

130.8423

275.0450

TS17

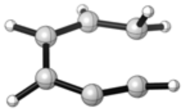

M08HX/6-31+G(d,p) = -232.025366

M08HX/6-31+G(d,p)+ZPVE = -231.930221

CCSD(T)-F12/cc-pVTZ-F12//M08HX/6-31+G(d,p) = -231.728972

Cartesian coordinates

C -1.241813 -0.811186 0.006943

C -1.259291 0.581828 0.167534

C -0.112930 1.356982 -0.078474

C 1.217984 0.920329 0.049528

C 0.002254 -1.416639 -0.043607

C 1.219599 -1.089613 -0.105510

H -2.156133 -1.316958 -0.302588

H -2.230598 1.079315 0.131506

H -0.294642 2.308496 -0.584590

H 1.979996 1.494592 -0.485510

H 2.196425 -1.405529 0.228610

H 1.550142 0.589877 1.034087

Frequencies

3193.7459

3199.7269

3340.1199

3117.6508

3158.6286

3178.1886

1497.1238

1557.9702

1944.7168

1256.8205

1400.9738

1473.1207

1100.5569

1134.1676

1189.4237

991.5191

1003.9333

1063.8227

799.8743

896.7003

971.8123

652.1404

712.2287

767.7061

439.1531

526.5122

568.5891

-575.5116

254.5480

372.3580

MIN21

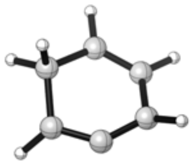

M08HX/6-31+G(d,p) = -232.066541

M08HX/6-31+G(d,p)+ZPVE = -231.969173

CCSD(T)-F12/cc-pVTZ-F12//M08HX/6-31+G(d,p) = -231.769017

Cartesian coordinates

C 1.243777 -0.792684 0.178412

C 0.057704 -1.348253 -0.027323

C -1.159309 -0.859292 -0.210411

C -1.247438 0.579003 0.274428

C 1.238846 0.646156 -0.220946

C 0.069172 1.294527 -0.065007

H 2.029110 -1.184693 0.824624

H -1.457656 0.711464 1.350614

H -1.904598 -1.273279 -0.888975

H -2.077370 1.066860 -0.256250

H 0.034405 2.382690 -0.147815

H 2.159596 1.180214 -0.457113

Frequencies

3190.2941

3194.4055

3202.9043

3016.1879

3092.0269

3178.1101

1456.8081

1647.6986

1886.9970

1297.0889

1357.5860

1423.1792

1153.4003

1172.4351

1277.5563

997.8298

1010.2133

1144.5351

872.4124

928.7794

972.8416

762.1281

804.8452

850.4337

525.3188

580.6706

698.0520

232.5855

364.8669

447.5560

TS44

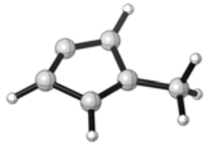

M08HX/6-31+G(d,p) = -232.010882

M08HX/6-31+G(d,p)+ZPVE = -231.917503

CCSD(T)-F12/cc-pVTZ-F12//M08HX/6-31+G(d,p) = -231.711497

Cartesian coordinates

C -0.079453 1.272985 -0.058012

C 0.593067 -0.037384 -0.082592

C 2.081599 -0.199728 0.009780

C -0.358196- 1.043531 0.058394

C -1.325445 0.775312 -0.009792

C -1.732839 -0.491768 0.076792

H 0.329408 2.213430 0.285939

H 2.597752 0.283427 -0.830502

H 2.326804 -1.270884 -0.002083

H 2.489185 0.217372 0.941958

H -0.185962 -2.108774 -0.098347

H -2.629583 -0.989894 -0.264388

Frequencies

3193.4496

3298.2362

3305.0018

3028.2541

3104.9863

3122.6703

1485.6895

1571.2919

1711.7735

1332.0035

1404.3863

1464.0887

1167.7569

1203.5370

1297.0850

975.8893

1051.5703

1069.6052

739.1535

877.2892

956.1206

653.5861

675.7713

706.8955

324.6944

325.8172

566.6039

-449.8454

132.3536

242.8775

MIN44

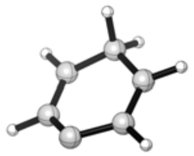

UM08HX/6-31+G(d,p) = -232.047000

UM08HX/6-31+G(d,p)+ZPVE = -231.950165

UCCSD(T)-F12/cc-pVTZ-F12//M08HX/6-31+G(d,p) = -231.745042

Cartesian coordinates

C -0.208473 -1.542058 0.000000

C 0.056850 -0.787973 1.213886

C 0.05685 0.567262 1.244839

C -0.039561 1.375282 -0.000000

C 0.056850 -0.787973 -1.213886

C 0.056850 0.567262 -1.244839

H 0.106711 -1.334660 2.159014

H 0.076034 1.107028 2.195770

H -0.985351 1.949597 -0.000000

H 0.743673 2.154850 -0.000000

H 0.076034 1.107028 -2.195770

H 0.106711 -1.334660 -2.159014

Frequencies

3167.7540

3166.3611

3147.9622

3147.9362

3032.7183

2997.8140

1652.5252

1597.0828

1443.8660

1433.5641

1385.8720

1367.3058

1274.9322

1214.0751

1173.7837

1135.8944

1029.5573

1024.4820

986.0126

980.4916

949.8989

912.2790

891.8741

773.0195

685.6936

552.4001

534.3139

380.0727

295.5731

170.7618

TS23

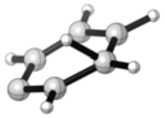

M08HX/6-31+G(d,p) = -232.02086

M08HX/6-31+G(d,p)+ZPVE = -231.926678

CCSD(T)-F12/cc-pVTZ-F12//M08HX/6-31+G(d,p) = -231.718211

Cartesian coordinates

C -1.212161 -0.738686 -0.005767

C -0.069429 -1.566864 -0.052225

C 1.134606 -0.811561 -0.025346

C 1.190303 0.662836 -0.059107

C -1.207597 0.671106 0.045492

C -0.023154 1.387535 -0.040567

H -2.197736 -1.214005 -0.057629

H 2.111436 -1.303000 0.030115

H 1.223826 0.032234 1.021602

H 2.151825 1.167538 -0.174282

H -0.011588 2.474758 -0.092955

H -2.153169 1.216278 0.098275

Frequencies

3166.3743

3174.7454

3219.2995

2223.2008

3126.8577

3138.3321

1448.3773

1557.0213

1613.2277

1310.3756

1330.7154

1406.2551

1141.0505

1187.3238

1199.9819

1031.2463

1067.0137

1089.1250

940.7339

967.4109

1023.6429

709.8284

848.0905

913.0195

567.7178

609.0239

660.4722

-790.0587

312.3639

358.3282

PR35frag1

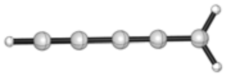

M08HX/6-31+G(d,p) = -232.02086

M08HX/6-31+G(d,p)+ZPVE = -192.054468

CCSD(T)-F12/cc-pVTZ-F12//M08HX/6-31+G(d,p) = -231.718211

Cartesian coordinates

C -0.000000 0.000000 2.639960

C 0.000000 -0.000000 -2.536974

C 0.000000 -0.000000 -1.175921

C -0.000000 0.000000 1.423473

C -0.000000 0.000000 0.061541

H 0.000000 -0.935090 -3.091543

H 0.000000 0.935090 -3.091543

H -0.000000 0.000000 3.710614

Frequencies

3168.9876

3270.3594

3476.2422

1462.5977

2025.4662

2168.3832

782.9507

1022.1546

1299.1740

732.3072

752.2281

780.1293

414.2488

624.7186

640.3722

159.1008

159.3907

357.5354

PR35frag2

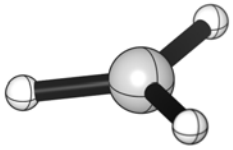

M08HX/6-31+G(d,p) = -232.02086

M08HX/6-31+G(d,p)+ZPVE = -39.798649

CCSD(T)-F12/cc-pVTZ-F12//M08HX/6-31+G(d,p) = -231.718211

Cartesian coordinates

C 0.000000 0.000000 0.000000

H 0.000000 -1.083479 -0.000000

H -0.938320 0.541739 -0.000000

H 0.938320 0.541739 -0.000000

Frequencies

3147.2841

3331.6225

3331.6501

533.0333

1410.2481

1410.2527

MIN22

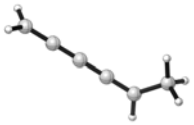

M08HX/6-31+G(d,p) = -232.063207

M08HX/6-31+G(d,p)+ZPVE = -231.968983

CCSD(T)-F12/cc-pVTZ-F12//M08HX/6-31+G(d,p) = -231.760304

Cartesian coordinates

C 0.527200 -1.862205 0.000000

C 2.005963 -2.147482 0.000000

C -1.559203 2.892792 -0.000000

C -1.030327 1.690013 -0.000000

C -0.000000 -0.657047 0.000000

C -0.515387 0.516588 -0.000000

H 2.284759 -2.736419 -0.884978

H 2.284759 -2.736419 0.884978

H -1.786058 3.407336 0.934603

H -1.786058 3.407336 -0.934603

H 2.588444 -1.219992 0.000000

H -0.155315 -2.717802 0.000000

Frequencies

3140.8128

3156.2718

3227.1784

3036.8698

3110.0277

3138.8397

1547.1122

1986.9573

2270.6657

1405.0196

1475.0142

1476.8616

1107.8451

1255.7179

1396.9859

984.9520

1009.8697

1050.8900

729.3361

810.2750

878.3445

515.7000

586.3185

604.3233

248.9297

335.9969

461.6843

105.0886

127.3746

178.1964

TS211

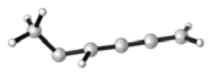

M08HX/6-31+G(d,p) = -231.9546

M08HX/6-31+G(d,p)+ZPVE = -231.86595

CCSD(T)-F12/cc-pVTZ-F12//M08HX/6-31+G(d,p) = -231.649791

Cartesian coordinates

C -1.874867 -0.456897 -0.390581

C -2.954541 0.434070 0.132648

C 3.272235 0.218959 0.010331

C 1.966632 0.026107 0.008321

C -0.590575 -0.328080 0.011472

C 0.709496 -0.157399 -0.003223

H -3.115032 1.147674 -0.693569

H -3.904002 -0.096527 0.261513

H 3.901682 -0.227503 0.778709

H 3.746449 0.828155 -0.757709

H -1.093564 -1.089721 0.766037

H -2.705818 1.017360 1.031215

Frequencies

3141.4637

3161.4999

3250.7545

2484.1586

3013.7695

3093.1555

1493.5278

1853.7259

2228.9008

1375.6234

1437.3414

1480.2745

1059.1106

1075.0571

1354.5338

830.7843

959.4265

1009.8157

591.8509

708.8682

824.3017

429.0273

498.0497

541.8340

185.6021

217.4513

369.8294

-651.1634

106.4606

136.5126

MIN149

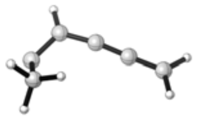

UM08HX/6-31+G(d,p) = -231.974885

UM08HX/6-31+G(d,p)+ZPVE = -231.882471

UCCSD(T)-F12/cc-pVTZ-F12//M08HX/6-31+G(d,p) = -231.673181

Cartesian coordinates

C 2.744398 -0.478498 -0.078809

C 1.548014 0.071658 0.028107

C 0.409879 0.619424 0.158186

C -0.830466 1.141762 0.093675

C -1.927997 -1.077829 0.101838

C -1.858087 0.300491 -0.423141

H 2.862455 -1.560096 -0.150341

H 3.644161 0.137092 -0.100744

H -1.041090 -1.433559 0.658774

H -0.968286 2.224601 0.150444

H -2.768070 -1.028145 0.821550

H -2.243609 -1.801942 -0.658820

Frequencies

3238.0386

3157.2025

3147.1152

3124.6711

3040.5097

2965.2403

2179.9423

1624.5991

1479.5235

1424.4936

1414.4377

1360.0459

1305.9851

1263.2762

1062.5457

1029.4095

955.1537

905.4443

893.6736

872.6531

813.2622

636.8379

565.6697

436.0564

347.5268

328.6378

278.0061

205.0738

158.0623

87.1190

TS200

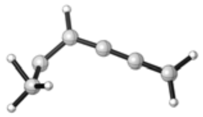

M08HX/6-31+G(d,p) = -231.956501

M08HX/6-31+G(d,p)+ZPVE = -231.86713

CCSD(T)-F12/cc-pVTZ-F12//M08HX/6-31+G(d,p) = -231.653787

Cartesian coordinates

C 2.854947 -0.454618 -0.065354

C 1.634089 0.041037 0.011923

C 0.463715 0.53108C 0.103306

C -0.771024 1.030473 0.084488

C -2.075473 -1.007178 0.118646

C -1.894373 0.255295 -0.441863

H 3.023548 -1.497013 -0.336002

H 3.726718 0.168179 0.136396

H -1.405390 -1.433278 0.886014

H -0.921675 2.090283 0.306043

H -2.852049 -0.029845 0.426077

H -2.842434 -1.674891 -0.285405

Frequencies

3157.4024

3166.0206

3236.7262

2228.1589

3016.0543

3147.8220

1528.5116

1675.3952

2203.1655

1323.7480

1416.7262

1438.6691

1052.6127

1109.1624

1293.0267

920.8184

1008.0040

1028.6254

683.9539

881.7984

887.0487

456.4234

576.5917

629.7396

250.6797

307.0481

358.5820

-1064.0847

87.3151

159.6920

MIN11

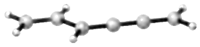

M08HX/6-31+G(d,p) = -232.076166

M08HX/6-31+G(d,p)+ZPVE = -231.981513

CCSD(T)-F12/cc-pVTZ-F12//M08HX/6-31+G(d,p) = -231.77552

Cartesian coordinates

C -0.320372 1.730629 0.000000

C -1.144471 2.787862 0.000000

C 1.555016 -2.780691 -0.000000

C 0.763367 -1.725386 -0.000000

C -0.799044 0.350518 0.000000

C -0.000000 -0.708703 -0.000000

H -2.228489 2.659429 0.000000

H -0.762073 3.807234 0.000000

H 2.639528 -2.669786 -0.000000

H 1.144275 -3.790603 -0.000000

H -1.882682 0.194483 0.000000

H 0.762465 1.873861 0.000000

Frequencies

3175.7808

3235.5587

3252.0284

3139.2317

3146.4042

3151.9848

1683.0613

1729.5893

2226.9341

1315.7659

1421.8687

1459.3587

1027.6663

1193.5463

1283.3162

949.9045

1009.9746

1022.8667

884.4457

891.8054

891.9678

526.8473

596.0652

694.4335

305.1992

388.4481

469.0761

108.4408

112.0844

254.4591

TS206

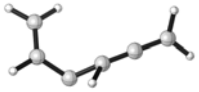

M08HX/6-31+G(d,p) = -231.9555

M08HX/6-31+G(d,p)+ZPVE = -231.866495

CCSD(T)-F12/cc-pVTZ-F12//M08HX/6-31+G(d,p) = -231.653709

Cartesian coordinates

C -1.990397 -0.280141 0.140023

C -2.112841 1.042938 -0.050960

C 2.868006 0.429671 0.022584

C 1.643037 -0.039961 0.017761

C -0.794332 -1.011108 -0.301408

C 0.435789 -0.510446 0.012694

H -1.262990 1.642307 -0.385194

H -3.070047 1.550641 0.069292

H 3.106461 1.400367 0.463959

H 3.693875 -0.132524 -0.420894

H 0.095394 -1.372976 0.793715

H -2.858261 -0.873535 0.434965

Frequencies

3174.4417

3196.8367

3241.7497

2361.4137

3115.0969

3142.2571

1612.8963

1662.3276

2149.1752

1316.9713

1414.4244

1432.2316

1025.6632

1033.6118

1170.6683

944.0000

969.2145

979.8571

677.5701

805.8219

925.9198

425.4456

575.8387

626.5829

231.3843

241.0666

365.3076

-801.1281

95.5988

155.4826

MIN112

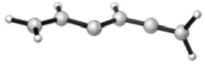

M08HX/6-31+G(d,p) = -231.991449

M08HX/6-31+G(d,p)+ZPVE = -231.899131

CCSD(T)-F12/cc-pVTZ-F12//M08HX/6-31+G(d,p) = -231.687857

Cartesian coordinates

C -1.287460 1.309773 0.000000

C -1.291394 2.657101 0.000000

C 1.996273 -2.308533 -0.000000

C 0.923373 -1.575924 -0.000000

C 0.000000 0.624054 0.000000

C -0.169604 -0.814922 0.000000

H -0.338293 3.188925 0.000000

H -2.213133 3.242665 0.000000

H 2.459901 -2.613419 -0.938052

H 2.459901 -2.613419 0.938052

H -1.147413 -1.322905 0.000000

H -2.248085 0.768854 0.000000

Frequencies

3157.1941

3244.2028

3245.4008

3045.4392

3049.5427

3139.3506

1439.9332

1665.2384

2023.2510

1314.6947

1362.9141

1422.2364

1093.8806

1165.4415

1185.0003

980.5333

1003.2041

1044.8857

860.7143

912.3464

957.4535

494.4175

574.2464

692.1804

315.7531

409.1603

457.4429

45.3758

89.4361

131.9166

TS223

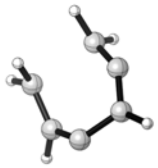

M08HX/6-31+G(d,p) = -231.957237

M08HX/6-31+G(d,p)+ZPVE = -231.864889

CCSD(T)-F12/cc-pVTZ-F12//M08HX/6-31+G(d,p) = -231.66393

Cartesian coordinates

C -0.807198 -1.086641 -0.221012

C 0.491773 -1.336810 0.114365

C 1.565861 -0.357739 0.051289

C 1.023454 0.854358 0.014119

C -1.562146 0.012577 0.235696

C -0.136101 1.549700 -0.167091

H -1.253840 -1.778372 -0.942873

H 2.614845 -0.621893 0.161122

H -2.589565 0.124120 -0.125472

H -0.280474 1.966105 -1.170386

H -0.503925 2.185049 0.636725

H -1.440895 0.312321 1.276681

Frequencies

3209.7320

3210.2364

3225.9766

3120.3808

3122.5986

3147.6200

1496.3234

1540.4753

1782.7238

1217.1176

1296.7876

1430.3900

1032.4897

1084.3553

1213.4001

954.3722

995.2606

1030.4023

852.0422

887.1692

893.0666

568.6367

699.7694

757.4438

363.8932

419.2311

543.6169

-507.9724

143.7137

296.9134

MIN64

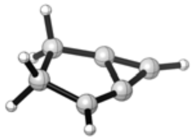

M08HX/6-31+G(d,p) = -232.033834

M08HX/6-31+G(d,p)+ZPVE = -231.936994

CCSD(T)-F12/cc-pVTZ-F12//M08HX/6-31+G(d,p) = -231.724454

Cartesian coordinates

C 0.574949 -1.239794 -0.000000

C 1.064233 0.005699 -0.000000

C 1.243641 1.432394 0.000000

C -0.000000 1.009960 0.000000

C -0.963866 -1.200692 -0.000000

C -1.330401 0.332908 0.000000

H 1.138816 -2.168830 -0.000000

H 1.946129 2.261072 0.000000

H -1.395113 -1.702773 -0.881123

H -1.913029 0.635227 -0.880369

H -1.913029 0.635227 0.880369

H -1.395113 -1.702773 0.881123

Frequencies

3123.4381

3230.4733

3237.4841

3020.6977

3057.7804

3073.1344

1503.4602

1701.3824

1881.0259

1307.3072

1314.5519

1444.2095

1157.0346

1230.8775

1235.7721

1005.1357

1012.6208

1090.7074

838.1646

911.6659

977.0085

761.9196

782.4569

798.9794

531.0432

692.2156

711.5372

76.3529

323.3681

476.0361

TS242

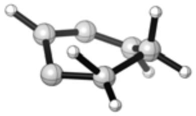

M08HX/6-31+G(d,p) = -231.954137

M08HX/6-31+G(d,p)+ZPVE = -231.861907

CCSD(T)-F12/cc-pVTZ-F12//M08HX/6-31+G(d,p) = -231.653599

Cartesian coordinates

C 1.415036 -0.549922 0.162454

C 0.313924 -1.262447 0.038000

C -0.963805 -1.046576 -0.240027

C -1.336236 0.298331 0.325243

C 1.174526 0.875007 -0.342420

C -0.168454 1.254039 -0.136663

H 2.340974 -0.804394 0.670900

H -1.389949 0.338527 1.423549

H -1.497482 -1.496937 -1.075104

H -2.300342 0.653167 -0.060954

H -0.475589 2.252204 -0.469388

H 0.712441 1.646841 0.671474

Frequencies

3142.4945

3209.6369

3233.1135

2241.0365

3053.9023

3119.8974

1418.6676

1452.7573

1895.3243

1249.5154

1372.0738

1408.2300

1165.8994

1202.1755

1239.1923

946.4474

1036.7362

1116.9357

810.4699

846.8121

912.7001

704.4816

751.8705

784.7855

450.1006

556.2792

576.5844

-892.4864

240.0680

345.6887

A-PR150frag1

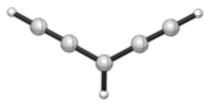

M08HX/6-31+G(d,p) = -231.954137

M08HX/6-31+G(d,p)+ZPVE = -192.049877

CCSD(T)-F12/cc-pVTZ-F12//M08HX/6-31+G(d,p) = -231.653599

Cartesian coordinates

C -0.000000 1.225874 -0.097287

C -0.000000 2.302680 0.472602

C 0.000000 -2.302680 0.472602

C 0.000000 -1.225874 -0.097287

C 0.000000 0.000000 -0.767328

H -0.000000 3.246069 0.978413

H 0.000000 -3.246069 0.978413

H 0.000000 0.000000 -1.856646

Frequencies

3192.0471

3476.5590

3480.6159

1377.8384

2074.5863

2156.2659

701.7296

967.8327

1126.4997

608.3360

688.4238

689.9472

404.6338

603.6794

607.6817

143.3037

364.2316

381.0659

A-PR150frag2

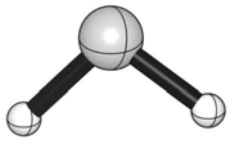

UM08HX/6-31+G(d,p) = -39.127031

UM08HX/6-31+G(d,p)+ZPVE = -39.107896

UCCSD(T)-F12/cc-pVTZ-F12//M08HX/6-31+G(d,p) = -39.075011

Cartesian coordinates

C 0.000000 0.000000 -0.176097

H 0.000000 0.866702 0.528290

H 0.000000 -0.866702 0.528290

Frequencies

1402.9010

2943.0663

3027.2733

A-MIN14

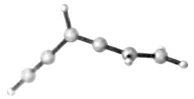

M08HX/6-31+G(d,p) = -39.124693

M08HX/6-31+G(d,p)+ZPVE = -231.327447

CCSD(T)-F12/cc-pVTZ-F12//M08HX/6-31+G(d,p) = -39.075012

Cartesian coordinates

C 0.496704 0.462156 0.096456

C 1.740901 0.119794 0.477672

C 2.653337 -0.593372 -0.311608

C -2.874705 -0.664472 0.113331

C -0.713700 0.812104 -0.273645

C -1.878490 -0.000639 -0.057080

H 2.045721 0.427144 1.482724

H -3.751214 -1.259266 0.268293

H 3.640566 -0.832709 0.070479

H -0.868264 1.772310 -0.779280

H 2.388898 -0.920904 -1.312970

Frequencies

3185.4179

3297.4861

3483.0737

2249.2784

3111.1942

3147.1031

1400.7479

1484.4726

1923.7360

1097.1937

1174.0023

1298.7594

886.4731

931.1656

1008.3920

697.1950

761.3930

812.8085

543.2571

596.7359

668.8174

296.5366

449.7212

487.7717

86.9688

163.2944

228.8753

A-TS100

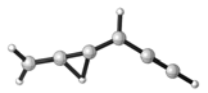

M08HX/6-31+G(d,p) = -39.124693

M08HX/6-31+G(d,p)+ZPVE = -231.248787

CCSD(T)-F12/cc-pVTZ-F12//M08HX/6-31+G(d,p) = -39.075012

Cartesian coordinates

C 0.762797 0.777006 -0.081671

C 1.978844 0.059782 0.004222

C 3.029856 -0.546175 0.052532

C -3.044218 -0.280697 0.048698

C -0.456260 0.205335 0.108595

C -1.726348 -0.067170 -0.076149

H -0.838476 -0.973274 -0.333696

H 3.955510 -1.080854 0.102680

H -3.546101 -1.087025 -0.476758

H -3.634956 0.403116 0.655556

H 0.795997 1.849546 -0.285143

Frequencies

3174.4189

3278.9382

3484.9867

2206.0131

2279.8998

3156.0141

1412.4030

1546.3875

1896.0760

1063.1969

1086.9826

1246.8490

727.6373

861.3978

935.9814

595.3689

701.9604

714.7146

478.8772

525.6762

591.0745

254.3854

301.6560

443.7086

-1364.6339

82.4384

148.5306

A-MIN6

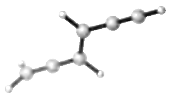

M08HX/6-31+G(d,p) = -192.753628

M08HX/6-31+G(d,p)+ZPVE = -231.340479

CCSD(T)-F12/cc-pVTZ-F12//M08HX/6-31+G(d,p) = -192.506702

Cartesian coordinates

C -0.000000 -0.650077 -0.000000

C -1.156479 -1.318893 -0.000000

C -2.282189 -1.976154 -0.000000

C 2.381799 2.037917 0.000000

C 0.092027 0.770852 0.000000

C 1.318767 1.441419 0.000000

H -2.772785 -2.261353 -0.933794

H -2.772785 -2.261353 0.933794

H 3.316986 2.558359 0.000000

H -0.822920 1.361173 0.000000

H 0.927958 -1.227219 -0.000000

Frequencies

3196.9376

3201.7641

3481.4857

2130.4729

3116.9941

3162.9730

1410.7563

1467.5622

1972.0338

1080.9570

1165.5331

1269.0623

911.7796

991.5538

1048.3995

691.9390

743.7994

904.5574

538.4637

547.0962

588.3606

314.4829

455.7142

529.4625

102.4174

126.9100

242.2243

A-TS112

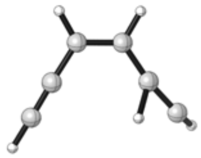

M08HX/6-31+G(d,p) = -192.753628

M08HX/6-31+G(d,p)+ZPVE = -231.246261

CCSD(T)-F12/cc-pVTZ-F12//M08HX/6-31+G(d,p) = -192.506702

Cartesian coordinates

C -1.521741 -0.063896 -0.034009

C -0.764103 1.133022 -0.049781

C 0.607463 1.124932 0.003620

C 1.388063 -0.035290 0.113332

C -2.142115 -1.107517 -0.000096

C 2.269272 -0.984129 0.005337

H -1.293099 2.082619 -0.093009

H 1.150699 2.070451 0.032777

H 1.046222 -1.079697 0.653118

H 2.778049 -1.456972 -0.845894

H -2.702902 -2.019135 0.022593

Frequencies

3188.7443

3219.0184

3484.0311

2201.5624

2387.5457

3067.6919

1387.0504

1531.9564

1915.5898

914.3490

1035.0698

1187.9783

741.9413

871.0346

912.3307

615.0684

697.1913

720.1637

476.9854

533.1304

598.7842

219.7645

294.2332

349.2139

-1654.3525

102.2555

170.7678

A-MIN24

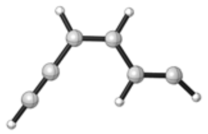

M08HX/6-31+G(d,p) = -192.753628

M08HX/6-31+G(d,p)+ZPVE = -231.314638

CCSD(T)-F12/cc-pVTZ-F12//M08HX/6-31+G(d,p) = -192.506702

Cartesian coordinates

C -0.000000 1.112264 -0.000000

C 1.298616 0.440740 -0.000000

C 2.458058 1.074455 -0.000000

C -1.447259 -2.162282 0.000000

C -1.183417 0.465164 0.000000

C -1.313315 -0.958766 0.000000

H 1.284466 -0.659322 -0.000000

H 3.510524 0.813745 -0.000000

H -1.564192 -3.226600 0.000000

H 0.003548 2.203173 -0.000000

H -2.110443 1.039549 0.000000

Frequencies

3200.1274

3276.2857

3480.9973

2236.9563

3079.9118

3182.7996

1407.6824

1649.5465

1701.1647

1126.6483

1228.1232

1251.5278

881.0447

949.7882

998.2268

708.0677

793.5421

802.1273

660.0593

693.6956

699.4700

304.9066

428.6524

503.9140

128.3615

147.9099

277.0691

A-TS12

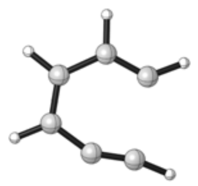

M08HX/6-31+G(d,p) = -192.753628

M08HX/6-31+G(d,p)+ZPVE = -231.301536

CCSD(T)-F12/cc-pVTZ-F12//M08HX/6-31+G(d,p) = -192.506702

Cartesian coordinates

C -0.487031 -0.814166 0.104435

C 0.543843 -1.472377 0.210002

C 1.836780 0.476199 -0.037705

C 1.039282 1.535797 -0.195667

C -1.179390 0.410754 -0.073922

C -0.420692 1.526999 -0.216116

H 1.273029 -2.249848 0.327223

H 2.922732 0.405739 -0.011580

H 1.510840 2.519004 -0.323997

H -0.921743 2.486238 -0.355210

H -2.267170 0.451232 -0.095619

Frequencies

3209.7916

3218.3805

3453.4024

2094.8494

3089.8062

3184.4276

1418.5979

1585.6715

1654.1248

1052.5173

1198.1303

1266.6805

902.1850

990.2235

1005.0749

758.0573

766.5579

893.9453

629.1462

668.1744

713.9279

374.3097

464.8340

479.0079

-522.4755

240.2781

292.7259

A-MIN1

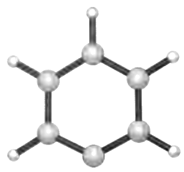

M08HX/6-31+G(d,p) = -192.753628

M08HX/6-31+G(d,p)+ZPVE = -231.418028

CCSD(T)-F12/cc-pVTZ-F12//M08HX/6-31+G(d,p) = -192.506702

Cartesian coordinates

C -0.000000 -0.000000 1.397853

C -0.000000 1.226652 0.771096

C 0.000000 1.213827 -0.631645

C 0.000000 0.000000 -1.323479

C -0.000000 -1.226652 0.771096

C -0.000000 -1.213827 -0.631645

H -0.000000 2.163810 1.327439

H 0.000000 2.156464 -1.180621

H 0.000000 0.000000 -2.413295

H -0.000000 -2.156464 -1.180621

H -0.000000 -2.163810 1.327439

Frequencies

3199.4666

3201.2583

3209.8151

1671.6233

3179.8913

3186.4063

1469.7287

1482.0763

1615.0366

1170.4577

1299.5770

1331.6157

1058.7664

1079.3082

1166.7015

978.0895

1002.7999

1033.5288

825.9562

898.5965

975.9445

607.5861

672.5083

720.4719

396.4599

422.5562

589.8075

A-PR129frag1

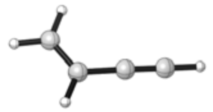

M08HX/6-31+G(d,p) = -154.697846

M08HX/6-31+G(d,p)+ZPVE = -154.636396

CCSD(T)-F12/cc-pVTZ-F12//M08HX/6-31+G(d,p) = -154.501632

Cartesian coordinates

C -0.000000 -0.745864 -0.000000

C -0.593648 0.559126 0.000000

C 0.113436 1.695408 0.000000

C 0.474575 -1.858703 -0.000000

H -1.685354 0.592399 0.000000

H -0.387896 2.662041 0.000000

H 1.203018 1.685166 0.000000

H 0.904049 -2.839405 -0.000000

Frequencies

3174.1707

3264.6216

3482.5152

1699.7295

2250.8878

3162.5889

1107.3439

1313.1379

1433.5556

895.5249

968.5780

1008.9904

674.6070

694.3379

717.2274

228.7588

337.5944

559.6372

A-MIN106

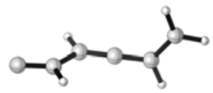

M08HX/6-31+G(d,p) = -39.124693

M08HX/6-31+G(d,p)+ZPVE = -231.259088

CCSD(T)-F12/cc-pVTZ-F12//M08HX/6-31+G(d,p) = -39.075012

Cartesian coordinates

C 0.465237 -0.408006 0.016179

C 1.758754 -0.301535 0.379365

C 2.698697 0.531637 -0.240827

C -3.168578 0.319024 -0.028925

C -0.788078 -0.532410 -0.343776

C -1.881624 0.292846 0.198799

H 2.084094 -0.916762 1.223989

H 3.724846 0.561207 0.111724

H 2.419854 1.158815 -1.082947

H -1.668710 1.070108 0.951968

H -1.066532 -1.282704 -1.089626

Frequencies

3141.9326

3184.8166

3296.9414

1923.4277

3080.3959

3136.2068

1399.5179

1484.7192

1771.4129

1116.8077

1174.2639

1302.1591

933.6907

972.9701

1052.9551

768.2467

816.8422

826.2729

534.2247

569.5911

643.9615

212.2329

271.4564

440.1059

90.1479

107.0812

176.8148

A-MIN62

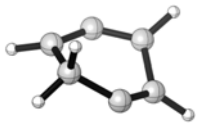

M08HX/6-31+G(d,p) = -39.124693

M08HX/6-31+G(d,p)+ZPVE = -231.292219

CCSD(T)-F12/cc-pVTZ-F12//M08HX/6-31+G(d,p) = -39.075012

Cartesian coordinates

C 0.060269 1.287021 -0.024872

C 1.246311 0.725502 0.185535

C 1.262665 -0.717004 -0.240396

C 0.075071 -1.286634 -0.059318

C -1.162815 0.802984 -0.194914

C -1.262627 -0.661349 0.251926

H 2.011033 1.102669 0.865251

H 2.172470 -1.260761 -0.491062

H -1.521499 -0.841475 1.308252

H -2.049075 -1.138709 -0.349135

H -1.926170 1.235147 -0.841073

Frequencies

3188.0263

3196.3612

3205.0266

1871.8452

3035.5579

3100.0564

1411.0484

1438.9300

1653.4992

1212.3064

1252.9300

1267.7038

984.5722

1142.3345

1161.0749

883.6218

907.2941

963.3194

752.5563

815.1393

849.9625

523.5835

581.5320

720.9845

247.3655

358.9731

390.7235

A-TS41

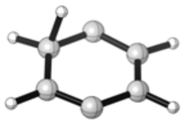

M08HX/6-31+G(d,p) = -39.124693

M08HX/6-31+G(d,p)+ZPVE = -231.275195

CCSD(T)-F12/cc-pVTZ-F12//M08HX/6-31+G(d,p) = -39.075012

Cartesian coordinates

C -0.037934 -1.404053 0.003937

C 1.214933 -0.752219 0.021564

C 1.251623 0.627383 -0.018269

C 0.078955 1.475879 -0.094077

C -1.220997 -0.776112 -0.041802

C -1.155320 0.703049 -0.003169

H 2.140002 -1.332953 0.057157

H 2.232834 1.108218 -0.046411

H -0.885186 1.039915 1.079265

H -2.092391 1.229734 -0.210102

H -2.182813 -1.288478 -0.089015

Frequencies

3155.2985

3172.8934

3192.0173

1645.9680

2566.9939

3137.8023

1351.8720

1434.0244

1510.7669

1228.2576

1242.0636

1287.1617

1035.0766

1036.7699

1167.4496

917.2151

957.6327

1010.3747

709.3619

823.4982

878.1216

571.8701

578.2413

614.3181

-509.1783

239.2406

379.7196

A-PR330frag1

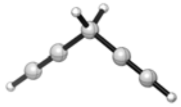

M08HX/6-31+G(d,p) = -192.74313

M08HX/6-31+G(d,p)+ZPVE = -192.676913

CCSD(T)-F12/cc-pVTZ-F12//M08HX/6-31+G(d,p) = -192.500182

Cartesian coordinates

C 0.000000 0.000000 -0.884784

C 0.000000 1.226588 -0.074151

C 0.000000 2.240203 0.579373

C 0.000000 -1.226588 -0.074151

C 0.000000 -2.240203 0.579373

H -0.881605 0.000000 -1.543381

H 0.000000 3.135538 1.166402

H 0.000000 -3.135538 1.166402

H 0.881605 -0.000000 -1.543381

Frequencies

3090.9463

3484.6124

3485.7526

2272.4169

2275.2221

3045.8291

1245.7596

1344.1877

1450.0892

919.9622

935.9253

1017.1092

682.2278

693.1056

695.7908

352.6273

571.4058

679.9956

138.8490

333.4074

350.5932

A-PR330frag2

M08HX/6-31+G(d,p) = -192.74313

M08HX/6-31+G(d,p)+ZPVE = -38.465931

CCSD(T)-F12/cc-pVTZ-F12//M08HX/6-31+G(d,p) = -192.500182

Cartesian coordinates

C 0.000000 -0.000000 0.160715

H -0.000000 0.000000 -0.964289

Frequencies

2874.9180

A-MIN171

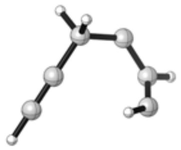

M08HX/6-31+G(d,p) = -192.74313

M08HX/6-31+G(d,p)+ZPVE = -231.209087

CCSD(T)-F12/cc-pVTZ-F12//M08HX/6-31+G(d,p) = -192.500182

Cartesian coordinates

C -1.351299 0.138071 -0.018961

C -0.351725 1.190817 0.222392

C 1.082411 0.932796 -0.119643

C 1.690553 -0.305235 -0.389237

C -2.166524 -0.728189 -0.230263

C 1.249334 -1.283186 0.429926

H -0.392455 1.514812 1.276730

H -0.640454 2.095417 -0.338402

H 2.605277 -0.381727 -0.979018

H 0.401104 -1.404822 1.097404

H -2.889973 -1.494132 -0.422004

Frequencies

3173.7893

3251.1326

3484.1196

2255.5656

3022.8749

3057.8423

1327.0050

1408.7105

1437.6420

1193.3172

1230.2518

1279.2649

867.0964

902.6561

955.9849

684.0603

811.6230

836.9302

541.5221

669.1591

681.1365

260.9569

309.2009

350.7660

100.4260

136.0628

230.3173

A-TS221

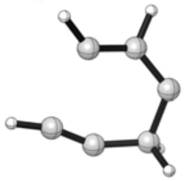

M08HX/6-31+G(d,p) = -192.74313

M08HX/6-31+G(d,p)+ZPVE = -231.196665

CCSD(T)-F12/cc-pVTZ-F12//M08HX/6-31+G(d,p) = -192.500182

Cartesian coordinates

C -1.312354 0.196965 -0.036772

C -0.888937 -1.200992 0.027684

C 0.615628 -1.324762 0.129150

C 1.436890 -0.224515 -0.162086

C -1.129643 1.406418 -0.063510

C 1.191943 1.072957 0.181556

H -1.357261 -1.739191 0.863699

H -1.186150 -1.756517 -0.877804

H 2.443851 -0.458318 -0.538905

H 1.871579 1.926662 0.198106

H -1.253173 2.470941 -0.101232

Frequencies

3090.2436

3183.0802

3458.3786

2132.1832

3038.1939

3073.2725

1268.2098

1366.5760

1413.8420

1152.5239

1215.6051

1252.4260

858.8267

931.1670

972.0800

715.1403

813.8393

835.1978

554.1692

673.5230

697.5630

324.5293

342.1551

447.0696

-420.8931

139.7074

273.6902

A-MIN65

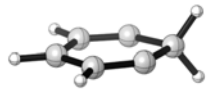

M08HX/6-31+G(d,p) = -192.74313

M08HX/6-31+G(d,p)+ZPVE = -231.289667

CCSD(T)-F12/cc-pVTZ-F12//M08HX/6-31+G(d,p) = -192.500182

Cartesian coordinates

C 0.000000 -1.294555 0.000000

C 1.398486 -0.812299 0.000000

C 1.124264 0.643304 -0.000000

C 0.014691 1.385890 -0.000000

C -1.209746 -0.684593 0.000000

C -1.274611 0.729892 -0.000000

H 1.965361 -1.138001 0.887476

H 1.965361 -1.138001 -0.887476

H 0.084511 2.475031 -0.000000

H -2.213419 1.280749 -0.000000

H -2.120319 -1.285606 0.000000

Frequencies

3179.3293

3185.2930

3215.0145

1661.4848

3007.0907

3042.1975

1341.2928

1422.5250

1538.5123

1224.2073

1241.5568

1323.5722

1007.1566

1070.1810

1131.5011

863.0666

912.3130

961.2210

825.7319

851.0397

851.7089

463.3133

556.5255

677.0868

217.3105

313.0852

361.6814

A-PR218frag1

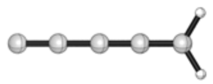

M08HX/6-31+G(d,p) = -191.431709

M08HX/6-31+G(d,p)+ZPVE = -191.390042

CCSD(T)-F12/cc-pVTZ-F12//M08HX/6-31+G(d,p) = -191.188387

Cartesian coordinates

C 0.000000 0.000000 -1.494317

C 0.000000 0.000000 -2.781065

C -0.000000 -0.000000 2.398744

C 0.000000 0.000000 -0.191045

C -0.000000 -0.000000 1.079770

H -0.000000 0.935117 2.963741

H 0.000000 -0.935117 2.963741

Frequencies

2241.2802

3121.3915

3213.2826

1373.3875

1524.6913

2011.4096

781.5703

978.4568

1026.2940

302.3936

504.5903

645.3481

140.3858

144.0043

281.4584

A-PR218frag2

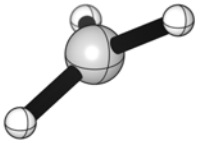

M08HX/6-31+G(d,p) = -191.431709

M08HX/6-31+G(d,p)+ZPVE = -39.798649

CCSD(T)-F12/cc-pVTZ-F12//M08HX/6-31+G(d,p) = -191.188387

Cartesian coordinates

C 0.000000 0.000000 0.000000

H -0.000000 -1.083469 -0.000000

H -0.938312 0.541734 -0.000000

H 0.938312 0.541734 -0.000000

Frequencies

3147.3764

3331.7215

3331.7491

532.9556

1410.2309

1410.2355

A-MIN5

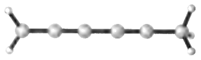

M08HX/6-31+G(d,p) = -191.431709

M08HX/6-31+G(d,p)+ZPVE = -231.345409

CCSD(T)-F12/cc-pVTZ-F12//M08HX/6-31+G(d,p) = -191.188387

Cartesian coordinates

C -0.776363 0.000017 0.000080

C -2.014554 -0.000066 0.000048

C -3.375917 -0.000165 -0.000005

C 3.262536 -0.000046 -0.000049

C 0.585368 0.000316 -0.000041

C 1.803899 0.000724 -0.000254

H -3.931642 -0.934712 -0.000070

H -3.931758 0.934313 -0.000002

H 3.650744 -0.630476 -0.810726

H 3.650278 -0.390275 0.950211

H 3.652568 1.016477 -0.138087

Frequencies

3128.7033

3167.1494

3266.9358

2292.9559

3039.4271

3122.9092

1467.9061

1476.2177

2031.6856

1359.2797

1409.2398

1467.4264

1028.0369

1038.6210

1044.9634

690.3983

747.6687

1022.5375

438.3241

587.2575

689.7028

252.9799

257.7478

389.4549

44.7508

106.3762

107.3608

A-PR233frag1

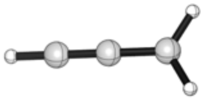

M08HX/6-31+G(d,p) = -191.431709

M08HX/6-31+G(d,p)+ZPVE = -115.928075

CCSD(T)-F12/cc-pVTZ-F12//M08HX/6-31+G(d,p) = -191.188387

Cartesian coordinates

C -0.000000 -0.000000 1.260382

C 0.000000 0.000000 -1.343612

C 0.000000 0.000000 -0.118714

H 0.000000 0.935087 1.812596

H -0.000000 -0.935087 1.812596

H 0.000000 0.000000 -2.413525

Frequencies

3181.8733

3285.2052

3475.1154

1077.5818

1452.7023

2033.9881

663.2943

694.8497

1030.8956

364.4951

421.9227

555.6212

A-PR233frag2

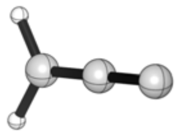

M08HX/6-31+G(d,p) = -115.300224

M08HX/6-31+G(d,p)+ZPVE = -115.269005

CCSD(T)-F12/cc-pVTZ-F12//M08HX/6-31+G(d,p) = -115.154272

Cartesian coordinates

C 0.000000 -0.000000 -1.125066

C -0.000000 0.000000 1.490224

C -0.000000 0.000000 0.199706

H -0.000000 0.933809 -1.694592

H -0.000000 -0.933809 -1.694592

Frequencies

2075.0599

3109.8592

3201.6389

1044.9848

1166.7799

1474.6031

283.6147

302.8409

1044.4025

A-MIN11

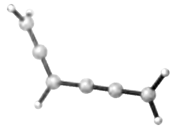

M08HX/6-31+G(d,p) = -115.300224

M08HX/6-31+G(d,p)+ZPVE = -231.337653

CCSD(T)-F12/cc-pVTZ-F12//M08HX/6-31+G(d,p) = -115.154272

Cartesian coordinates

C -1.124658 0.135797 -0.000000

C -1.047903 1.461531 -0.000000

C -0.986915 2.762994 -0.000000

C 2.039423 -2.325189 0.000000

C -0.000000 -0.714989 -0.000000

C 0.966028 -1.485575 0.000000

H -0.956798 3.325737 0.934958

H -0.956798 3.325737 -0.934958

H 3.052169 -1.930569 0.000000

H 1.902109 -3.403375 0.000000

H -2.116533 -0.324941 -0.000000

Frequencies

3169.7048

3213.9101

3270.1386

2088.1602

3130.7938

3146.6792

1448.2576

1499.8999

1973.0648

1078.6160

1271.6735

1377.8726

893.5957

992.7446

1030.0287

741.7779

842.6014

886.1126

442.4907

567.1950

604.7209

260.7086

333.6826

394.1634

94.6557

182.9319

247.2114

A-TS125

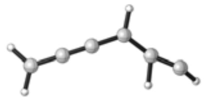

M08HX/6-31+G(d,p) = -115.300224

M08HX/6-31+G(d,p)+ZPVE = -231.240362

CCSD(T)-F12/cc-pVTZ-F12//M08HX/6-31+G(d,p) = -115.154272

Cartesian coordinates

C 0.726148 0.811158 -0.012568

C 1.818508 -0.013800 -0.094072

C 3.017041 -0.517473 0.203418

C -3.053984 -0.409471 0.008778

C -0.580601 0.374440 0.000903

C -1.770872 -0.000685 0.003928

H 1.703476 -1.192098 -0.068728

H 3.595086 -0.999469 -0.602453

H -3.867442 0.311732 0.044589

H -3.302961 -1.467840 -0.022036

H 0.934402 1.882656 -0.013700

Frequencies

3166.3533

3171.7296

3263.5370

2023.1964

2525.0859

3053.9649

1420.4773

1545.2221

1699.0632

1051.7262

1118.8369

1287.4181

824.3889

877.6932

1026.0799

581.2456

751.4795

774.1275

440.4479

465.6470

539.9139

244.8082

311.9659

355.1067

-1011.3615

100.4455

173.4175

A-MIN43

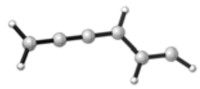

M08HX/6-31+G(d,p) = -115.300224

M08HX/6-31+G(d,p)+ZPVE = -231.304556

CCSD(T)-F12/cc-pVTZ-F12//M08HX/6-31+G(d,p) = -115.154272

Cartesian coordinates

C 0.889896 -0.337045 -0.000000

C 0.521349 -1.754047 0.000000

C 1.396653 -2.744870 -0.000000

C -1.725198 2.579080 0.000000

C -0.000000 0.646709 -0.000000

C -0.846598 1.595076 0.000000

H -0.553656 -1.981700 0.000000

H 1.379192 -3.828580 0.000000

H -1.401667 3.620108 -0.000000

H -2.796277 2.376331 0.000000

H 1.955802 -0.095574 -0.000000

Frequencies

3160.9026

3237.8822

3279.9620

2215.9896

3085.2232

3148.4126

1437.0939

1652.5115

1711.1995

1149.2057

1224.4984

1308.8758

890.4926

959.7452

1026.3610

779.0968

863.2476

889.4829

525.9861

594.0400

689.1250

304.5977

384.1635

487.8634

110.4631

117.6391

254.6888

A-TS109

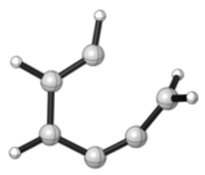

M08HX/6-31+G(d,p) = -115.300224

M08HX/6-31+G(d,p)+ZPVE = -231.247067

CCSD(T)-F12/cc-pVTZ-F12//M08HX/6-31+G(d,p) = -115.154272

Cartesian coordinates

C -0.105865 -1.361358 -0.139904

C 1.018776 -0.799574 -0.082394

C 1.889933 0.219001 0.120260

C -0.192725 1.425904 -0.109499

C -1.333725 -0.754115 0.039866

C -1.313611 0.683104 0.033271

H 2.647594 0.174380 0.907579

H 2.068856 0.935338 -0.680349

H -0.014695 2.495759 -0.062553

H -2.242852 1.194773 0.311303

H -2.235601 -1.278022 0.354420

Frequencies

3196.8706

3224.4825

3261.1427

1984.5709

3116.0398

3127.1081

1382.6994

1470.1027

1531.2614

1077.2623

1210.7015

1322.5375

905.9005

1002.6559

1032.1989

791.3275

868.5678

885.6569

561.0891

634.5238

657.3209

372.5027

426.4722

521.3727

-575.6150

217.7892

329.5460

A-MIN15

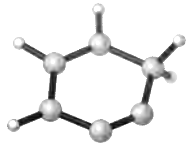

M08HX/6-31+G(d,p) = -115.300224

M08HX/6-31+G(d,p)+ZPVE = -231.325784

CCSD(T)-F12/cc-pVTZ-F12//M08HX/6-31+G(d,p) = -115.154272

Cartesian coordinates

C 0.027305 -1.418819 0.000000

C 1.100298 -0.819846 -0.000000

C 1.399072 0.623747 -0.000000

C -0.000000 1.259992 -0.000000

C -1.277365 -0.872819 0.000000

C -1.182029 0.564652 0.000000

H 1.980117 0.949092 0.880233

H 1.980117 0.949092 -0.880233

H -0.030650 2.350542 -0.000000

H -2.111928 1.136584 0.000000

H -2.221351 -1.406751 0.000000

Frequencies

3176.1265

3194.0872

3254.7657

2122.6729

3002.3757

3031.4829

1371.7359

1438.4593

1526.8832

1181.9169

1271.5933

1305.7342

1003.9907

1150.8037

1163.2623

910.1414

945.3243

977.1862

723.2006

798.8629

876.3384

469.0825

564.0527

641.7765

210.8655

358.8145

417.5146

A-TS23

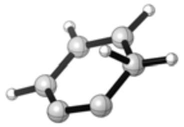

M08HX/6-31+G(d,p) = -115.300224

M08HX/6-31+G(d,p)+ZPVE = -231.287325

CCSD(T)-F12/cc-pVTZ-F12//M08HX/6-31+G(d,p) = -115.154272

Cartesian coordinates

C -0.335496 -1.394897 -0.031130

C -1.352784 -0.485880 0.008725

C -1.005279 0.905868 0.024265

C 0.308615 1.282159 -0.041824

C 1.015858 -1.162881 -0.072582

C 1.346433 0.265866 -0.023593

H -2.407166 -0.772869 -0.001011

H -1.794570 1.658859 0.046481

H 0.587775 2.335025 -0.092484

H 1.367393 -0.261697 1.042086

H 2.382485 0.579270 -0.178239

Frequencies

3163.9152

3183.6843

3202.7214

1660.7430

2421.6659

3151.6135

1401.3937

1452.3335

1498.1519

1173.8860

1200.2725

1358.8623

1029.2748

1064.7858

1170.1247

929.2540

979.0356

1001.4184

696.5801

838.8851

867.8284

476.9436

591.4823

638.5786

-655.2543

280.6896

348.0317

A-PR14frag1

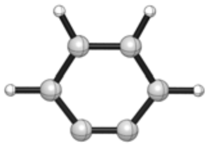

M08HX/6-31+G(d,p) = -230.855749

M08HX/6-31+G(d,p)+ZPVE = -230.780105

CCSD(T)-F12/cc-pVTZ-F12//M08HX/6-31+G(d,p) = -230.564185

Cartesian coordinates

C 0.000000 0.623477 -1.233361

C -0.000000 -0.623477 -1.233361

C -0.000000 -1.465217 -0.133041

C -0.000000 -0.704516 1.053820

C 0.000000 1.465217 -0.133041

C 0.000000 0.704516 1.053820

H -0.000000 -2.553243 -0.135575

H -0.000000 -1.228055 2.011068

H 0.000000 1.228055 2.011068

H 0.000000 2.553243 -0.135575

Frequencies

3198.1163

3220.5836

3224.1148

1509.2856

2063.8665

3183.4531

1322.7889

1442.4684

1497.3636

1115.2040

1157.4714

1269.6598

983.9124

1010.7404

1086.7512

839.5162

878.8154

938.3217

610.5863

616.6409

753.7833

389.5891

430.0926

460.9487

A-PR14frag2

M08HX/6-31+G(d,p) = -230.855749

M08HX/6-31+G(d,p)+ZPVE = -0.499294

CCSD(T)-F12/cc-pVTZ-F12//M08HX/6-31+G(d,p) = -230.564185

Cartesian coordinates

H 0.000000 0.000000 0.000000

A-MIN13

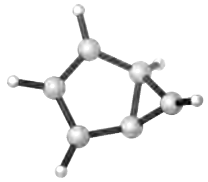

M08HX/6-31+G(d,p) = -230.855749

M08HX/6-31+G(d,p)+ZPVE = -231.328857

CCSD(T)-F12/cc-pVTZ-F12//M08HX/6-31+G(d,p) = -230.564185

Cartesian coordinates

C 0.648676 -0.707873 -0.327740

C 1.647268 -0.189245 0.370587

C 0.701535 0.798317 -0.310714

C -0.672425 1.136280 0.136772

C -0.686660 -1.177017 -0.142099

C -1.449126 -0.017432 0.151510

H 2.323285 -0.326715 1.208141

H 1.109514 1.381067 -1.143608

H -1.060965 2.145940 0.253189

H -2.500052 -0.051672 0.435148

H -1.007390 -2.206791 -0.022763

Frequencies

3229.4103

3251.1512

3254.4722

1624.8174

3103.1638

3210.5564

1280.8864

1373.9141

1462.9400

1099.3168

1129.4920

1256.4400

1004.9456

1050.1186

1054.9167

901.9659

916.1616

959.4077

764.3312

790.4486

815.6727

592.0364

696.5188

718.7961

257.1310

338.8941

509.9227

A-TS4

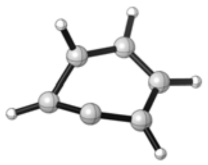

M08HX/6-31+G(d,p) = -230.855749

M08HX/6-31+G(d,p)+ZPVE = -231.322241

CCSD(T)-F12/cc-pVTZ-F12//M08HX/6-31+G(d,p) = -230.564185

Cartesian coordinates

C -0.587868 -0.884118 0.198120

C 0.786177 -1.146153 0.138914

C 1.459615 0.067083 -0.129083

C 0.603813 1.162599 -0.104819

C -1.625159 -0.249780 -0.298444

C -0.774442 0.839056 0.209906

H 1.215336 -2.141851 0.077053

H 2.518333 0.119694 -0.382967

H 0.950178 2.195251 -0.097146

H -1.224606 1.455858 1.003585

H -2.632066 -0.361074 -0.688092

Frequencies

3217.1080

3238.1126

3243.8613

1733.0852

3032.3665

3198.6887

1331.4357

1417.1411

1487.0173

1111.1198

1169.4021

1287.3247

1036.1027

1072.8476

1083.5209

822.2681

919.9930

936.2232

708.1360

785.8632

809.5276

546.8662

594.9917

670.4815

-611.2909

338.6279

431.4031

A-PR82frag1

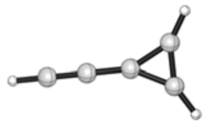

M08HX/6-31+G(d,p) = -230.855749

M08HX/6-31+G(d,p)+ZPVE = -192.011789

CCSD(T)-F12/cc-pVTZ-F12//M08HX/6-31+G(d,p) = -230.564185

Cartesian coordinates

C -0.000000 0.000000 -1.105717

C -0.000000 0.000000 -2.336569

C 0.000000 -0.660210 1.532324

C 0.000000 -0.000000 0.246770

C -0.000000 0.660210 1.532324

H -0.000000 0.000000 -3.405616

H 0.000000 -1.585737 2.095411

H -0.000000 1.585737 2.095411

Frequencies

3264.1771

3314.7493

3483.2846

1504.8760

1729.0197

2070.5659

870.8908

971.1179

1032.6841

698.1534

722.3803

793.0311

421.2093

526.3076

607.9081

43.9574

207.0360

305.7995

A-PR82frag2

UM08HX/6-31+G(d,p) = -39.127031

UM08HX/6-31+G(d,p)+ZPVE = -39.107896

UCCSD(T)-F12/cc-pVTZ-F12//M08HX/6-31+G(d,p) = -39.075011

Cartesian coordinates

C 0.000000 0.000000 -0.176097

H 0.000000 0.866702 0.528290

H 0.000000 -0.866702 0.528290

Frequencies

1402.9010

2943.0663

3027.2733

A-MIN53

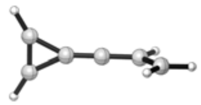

M08HX/6-31+G(d,p) = -230.855749

M08HX/6-31+G(d,p)+ZPVE = -231.300137

CCSD(T)-F12/cc-pVTZ-F12//M08HX/6-31+G(d,p) = -39.054513

Cartesian coordinates

C 0.301714 -0.359018 0.000000

C 0.627970 -1.681219 -0.000000

C -0.288585 -2.728089 -0.000000

C -0.288585 2.169454 -0.656434

C 0.010817 0.897792 0.000000

C -0.288585 2.169454 0.656434

H 0.048115 -3.761370 -0.000000

H -1.358483 -2.533753 -0.000000

H -0.415418 2.712255 -1.586531

H -0.415418 2.712255 1.586531

H 1.692722 -1.939634 0.000000

Frequencies

3247.0335

3281.5362

3293.3782

2005.8907

3121.5409

3172.4191

1422.5333

1492.0878

1685.5362

1049.9585

1193.9767

1268.9113

934.6056

942.6351

1042.0498

792.8595

849.7468

883.0039

564.6719

646.2500

686.8481

359.0179

440.4313

493.0037

120.8363

125.7438

247.1770

A-TS204

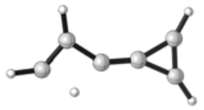

M08HX/6-31+G(d,p) = -230.855749

M08HX/6-31+G(d,p)+ZPVE = -231.205636

CCSD(T)-F12/cc-pVTZ-F12//M08HX/6-31+G(d,p) = -39.054513

Cartesian coordinates

C -0.489431 -0.162578 0.000033

C -1.713641 0.619848 0.000008

C -2.591317 -0.390615 -0.000019

C 2.149984 -0.655513 -0.000006

C 0.824470 -0.075037 0.000012

C 2.086553 0.658885 -0.000014

H -1.860906 1.701108 0.000006

H -3.673356 -0.525649 -0.000059

H 2.761050 -1.550803 -0.000012

H 2.602102 1.611804 -0.000030

H -1.428596 -1.206400 0.000015

Frequencies

3213.1311

3262.0597

3307.5687

1888.5492

1903.1915

3175.5331

1310.1684

1612.2637

1649.6252

1036.5862

1072.4604

1131.5233

906.9567

942.0657

1004.2290

830.6901

887.8271

899.9186

534.1309

602.0637

653.4279

225.2651

376.0558

490.6368

-2323.0195

121.9904

158.6467

A-MIN68

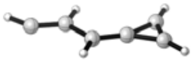

M08HX/6-31+G(d,p) = -230.855749

M08HX/6-31+G(d,p)+ZPVE = -231.285926

CCSD(T)-F12/cc-pVTZ-F12//M08HX/6-31+G(d,p) = -39.054513

Cartesian coordinates

C -0.000000 -0.770414 0.000000

C -1.455171 -0.632445 0.000000

C -2.338353 -1.616553 0.000000

C 2.102280 0.925098 -0.000000

C 0.810341 0.295993 -0.000000

C 1.045410 1.719059 -0.000000

H -1.838295 0.401201 -0.000000

H -3.417101 -1.716260 0.000000

H 3.181673 0.830910 -0.000000

H 0.656427 2.730217 -0.000000

H 0.430253 -1.770494 0.000000

Frequencies

3265.4059

3280.2406

3307.6185

1860.4841

3044.3743

3200.8629

1348.7818

1626.2832

1676.0361

1118.6917

1158.8676

1231.9182

907.2623

941.5979

1041.9904

827.6526

845.6460

879.4163

636.1257

685.9707

780.3033

387.3478

444.8170

504.9267

133.6319

149.8375

273.9723

A-TS209

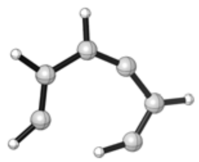

M08HX/6-31+G(d,p) = -230.855749

M08HX/6-31+G(d,p)+ZPVE = -231.202747

CCSD(T)-F12/cc-pVTZ-F12//M08HX/6-31+G(d,p) = -39.054513

Cartesian coordinates

C 0.987921 -0.026760 -2.444003

C 1.744052 -0.984473 -1.704491

C 1.596156 -0.752759 -0.408797

C -0.249049 1.527073 -0.111287

C 0.160566 1.006374 -2.511834

C -0.450763 1.748689 -1.399431

H 2.337242 -1.776723 -2.160353

H 1.069980 -0.026092 0.219136

H -0.594225 1.944990 0.828115

H -1.137156 2.558700 -1.683234

H -0.105031 1.347185 -3.517427

Frequencies

3133.4715

3182.4011

3272.3455

1765.5181

3081.9909

3104.7778

1316.9358

1569.9515

1659.6549

1089.8509

1256.4202

1263.7079

879.0083

897.4286

913.7046

695.2136

768.5928

855.9587

602.8486

615.3008

679.2327

200.0310

319.4330

461.5968

-244.3794

34.2069

170.1376

B-PR134frag1

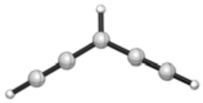

M08HX/6-31+G(d,p) = -230.855749

M08HX/6-31+G(d,p)+ZPVE = -192.049877

CCSD(T)-F12/cc-pVTZ-F12//M08HX/6-31+G(d,p) = -39.054513

Cartesian coordinates

C -0.000000 0.000000 -0.767328

C -0.000000 -1.225874 -0.097287

C -0.000000 1.225874 -0.097287

C 0.000000 2.302680 0.472602

C 0.000000 -2.302680 0.472602

H 0.000000 3.246068 0.978413

H -0.000000 0.000000 -1.856646

H 0.000000 -3.246068 0.978413

Frequencies

3192.0470

3476.5590

3480.6158

1377.8385

2074.5866

2156.2662

701.7296

967.8328

1126.4998

608.3361

688.4238

689.9473

404.6337

603.6796

607.6818

143.3032

364.2315

381.0659

B-PR134frag2

M08HX/6-31+G(d,p) = -230.855749

M08HX/6-31+G(d,p)+ZPVE = -38.465931

CCSD(T)-F12/cc-pVTZ-F12//M08HX/6-31+G(d,p) = -39.054513

Cartesian coordinates

C 0.000000 -0.000000 0.160715

H -0.000000 0.000000 -0.964289

Frequencies

2874.9176

B-MIN178

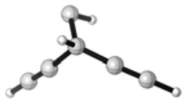

M08HX/6-31+G(d,p) = -230.687036

M08HX/6-31+G(d,p)+ZPVE = -230.619016

CCSD(T)-F12/cc-pVTZ-F12//M08HX/6-31+G(d,p) = -230.395182

Cartesian coordinates

C 1.346414 -0.111791 0.118462

C 2.417238 -0.552179 -0.221802

C -2.111923 -0.896538 -0.158489

C -1.104763 -0.355108 0.240223

C 0.073585 0.491524 0.524450

C -0.642239 1.481946 -0.366933

H -0.303939 1.217523 -1.393162

H 3.362195 -0.952716 -0.527610

H -3.003174 -1.400149 -0.476238

H 0.075054 0.788218 1.581543

Frequencies

3089.1025

3473.2724

3484.3617

2219.7997

2265.6781

2997.6493

1119.7241

1237.5906

1349.2405

884.4422

904.5830

1113.9149

704.9226

730.5435

764.5505

561.9093

668.6219

681.1389

288.9971

335.0643

530.4115

112.5136

141.8964

197.4596

B-TS154

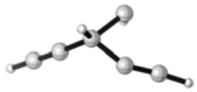

M08HX/6-31+G(d,p) = -230.686307

M08HX/6-31+G(d,p)+ZPVE = -230.617949

CCSD(T)-F12/cc-pVTZ-F12//M08HX/6-31+G(d,p) = -230.395251

Cartesian coordinates

C 1.046969 -0.465874 0.316149

C 0.790907 1.276719 -0.380657

C 2.123014 -0.741092 -0.183724

C -2.480536 -0.422996 -0.259302

C -1.388484 -0.069115 0.112945

C -0.087981 0.428290 0.554953

H -0.099883 0.796863 1.587168

H 0.448138 0.974207 -1.393977

H 3.073876 -1.061297 -0.561751

H -3.445455 -0.745358 -0.593626

Frequencies

3110.2339

3469.9893

3480.7552

2173.3246

2265.0250

2997.8568

1127.2763

1241.7048

1350.2995

898.3817

943.2502

1093.3292

710.6679

743.1938

851.5933

572.4471

656.4823

686.5061

317.6278

439.7594

529.3325

-167.3251

142.7506

204.2321

B-MIN34

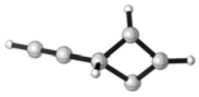

M08HX/6-31+G(d,p) = -230.756163

M08HX/6-31+G(d,p)+ZPVE = -230.684319

CCSD(T)-F12/cc-pVTZ-F12//M08HX/6-31+G(d,p) = -230.459992

Cartesian coordinates

C 1.028497 -0.896107 -0.380862

C 0.944444 0.839213 0.050075

C 2.045763 -0.017146 -0.091171

C -2.587368 0.031346 -0.229018

C -1.430892 -0.050913 0.106057

C -0.043351 -0.140051 0.524507

H 0.083994 -0.464259 1.558138

H 0.684011 1.691811 -0.587080

H 3.101344 0.075904 -0.315354

H -3.611905 0.098497 -0.533229

Frequencies

3168.0294

3293.5515

3483.9013

1515.3917

2273.0013

3123.8765

1250.9468

1337.9703

1389.1140

1043.3779

1073.0123

1163.1091

809.1239

908.1830

1020.6679

696.8339

706.9707

751.1839

482.5480

580.5880

633.8132

181.4745

198.0583

450.8694

B-TS21

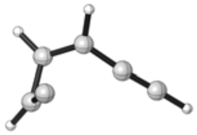

M08HX/6-31+G(d,p) = -230.738779

M08HX/6-31+G(d,p)+ZPVE = -230.668802

CCSD(T)-F12/cc-pVTZ-F12//M08HX/6-31+G(d,p) = -230.442469

Cartesian coordinates

C -1.481064 -0.745265 -0.374290

C -1.131131 0.661472 -0.359961

C 0.113859 0.935904 0.170165

C 2.345246 -0.433350 -0.155687

C -1.063119 -0.737149 0.892290

C 1.295048 0.145864 -0.003282

H -2.117986 -1.354786 -1.002162

H -1.889901 1.449150 -0.360577

H 0.273533 1.910867 0.640670

H 3.261320 -0.970087 -0.293337

Frequencies

3157.4681

3296.1156

3484.1994

1636.6387

2251.9585

3138.9302

1229.5122

1398.5481

1520.8496

975.1824

1018.5764

1167.5320

771.0656

867.5742

938.9438

666.3702

681.4072

703.0802

404.2045

451.1669

563.8675

-297.1414

175.5242

217.9430

B-MIN2

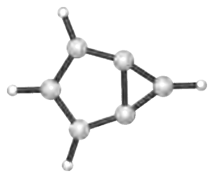

M08HX/6-31+G(d,p) = -230.844005

M08HX/6-31+G(d,p)+ZPVE = -230.769374

CCSD(T)-F12/cc-pVTZ-F12//M08HX/6-31+G(d,p) = -230.539001

Cartesian coordinates

C 0.000000 1.172296 -0.667495

C 0.000000 0.000000 -1.457940

C -0.000000 -1.172296 -0.667495

C -0.000000 -0.770243 0.658661

C -0.000000 -0.000000 1.769590

C 0.000000 0.770243 0.658661

H 0.000000 2.193186 -1.036359

H 0.000000 0.000000 -2.550944

H -0.000000 -2.193186 -1.036359

H -0.000000 -0.000000 2.859765

Frequencies

3192.1777

3247.6537

3251.1055

1594.4726

1874.9493

3179.3226

1292.4529

1384.4585

1426.7360

1066.0598

1108.8807

1147.2890

912.6989

986.7321

1065.7486

818.5960

823.0766

830.3069

633.4077

646.3616

798.6877

303.3636

585.1827

589.5639

B-TS14

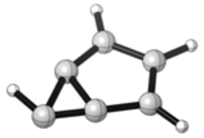

M08HX/6-31+G(d,p) = -230.743816

M08HX/6-31+G(d,p)+ZPVE = -230.673945

CCSD(T)-F12/cc-pVTZ-F12//M08HX/6-31+G(d,p) = -230.443783

Cartesian coordinates

C 1.289089 -0.389827 -0.000000

C 1.078299 0.988845 -0.000000

C -0.321780 1.303126 0.000000

C -1.018192 0.111719 0.000000

C -1.223001 -1.450100 0.000000

C -0.000000 -0.947110 0.000000

H 2.233887 -0.920572 -0.000000

H 1.877674 1.732931 -0.000000

H -0.754454 2.298665 0.000000

H -2.183602 -0.810934 0.000000

Frequencies

3186.8905

3245.5249

3277.6131

1537.3569

1845.1618

2846.6965

1263.2434

1352.0831

1396.3874

1017.1297

1052.0293

1109.2022

825.4891

912.7444

968.3832

696.1249

779.7438

820.0055

550.2001

572.7364

638.1821

-1591.1063

258.0998

518.7111

B-PR113frag1

M08HX/6-31+G(d,p) = -230.743816

M08HX/6-31+G(d,p)+ZPVE = -38.465931

CCSD(T)-F12/cc-pVTZ-F12//M08HX/6-31+G(d,p) = -230.443783

Cartesian coordinates

C 0.000000 -0.000000 0.160715

H -0.000000 0.000000 -0.964289

Frequencies

2874.9176

B-PR113frag2

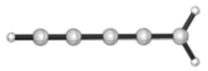

M08HX/6-31+G(d,p) = -230.743816

M08HX/6-31+G(d,p)+ZPVE = -192.054468

CCSD(T)-F12/cc-pVTZ-F12//M08HX/6-31+G(d,p) = -230.443783

Cartesian coordinates

C -0.000000 -0.000000 -0.061540

C 0.000000 0.000000 1.175923

C -0.000000 -0.000000 -1.423473

C 0.0000000.000000 2.536976

C -0.000000-0.000000 -2.639961

H 0.000000-0.935093 3.091534

H -0.0000000.935093 3.091534

H -0.000000-0.000000 -3.710615

Frequencies

3169.0106

3270.3893

3476.2371

1462.5906

2025.4603

2168.3802

782.9511

1022.1528

1299.1727

732.3064

752.2116

780.1280

414.2479

624.7204

640.3741

159.0992

159.3862

357.5325

B-MIN146

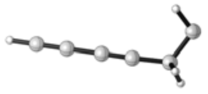

M08HX/6-31+G(d,p) = -230.704849

M08HX/6-31+G(d,p)+ZPVE = -230.635968

CCSD(T)-F12/cc-pVTZ-F12//M08HX/6-31+G(d,p) = -230.408465

Cartesian coordinates

C 2.004442 -0.456786 -0.008730

C 0.547731 -0.311666 0.016960

C -0.653932 -0.152207 0.003980

C -2.021764 0.029752 -0.003663

C 2.893047 0.720367 -0.096782

C -3.221235 0.189438 -0.009654

H 2.284202 1.593296 0.231204

H 2.378575 -0.741698 1.011953

H 2.330226 -1.295855 -0.639653

H -4.282741 0.330863 -0.016167

Frequencies

2984.5730

3084.3331

3480.8841

2213.3476

2400.8970

2875.3629

1225.6445

1310.8206

1387.5606

985.4795

1116.6138

1178.5895

717.0153

808.8867

823.4072

634.7347

652.0108

671.0296

328.0945

371.7376

483.8629

101.3154

146.1361

253.0864

B-TS455

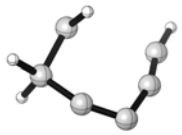

M08HX/6-31+G(d,p) = -230.627769

M08HX/6-31+G(d,p)+ZPVE = -230.56015

CCSD(T)-F12/cc-pVTZ-F12//M08HX/6-31+G(d,p) = -230.336771

Cartesian coordinates

C -0.680363 -0.976420 0.068943

C -1.564276 0.134848 -0.235448

C -0.771659 1.381374 0.186805

C 1.694937 0.784831 -0.157853

C 1.288419 -0.360244 0.000687

C 0.450874 -1.498429 0.121406

H -1.889271 0.141688 -1.282603

H -2.447477 0.202679 0.427400

H -0.439360 1.178594 1.225880

H 2.268519 1.681286 -0.277923

Frequencies

3029.4735

3118.1022

3471.9728

2017.5978

2125.5775

3015.7358

1158.2395

1286.0830

1432.2649

950.8709

1061.0434

1083.0849

709.7205

842.2035

922.8261

530.1075

606.7765

665.4462

308.0395

408.0391

447.9143

-288.5891

206.8853

283.6297

B-MIN143

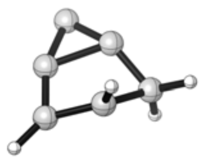

M08HX/6-31+G(d,p) = -230.711016

M08HX/6-31+G(d,p)+ZPVE = -230.639361

CCSD(T)-F12/cc-pVTZ-F12//M08HX/6-31+G(d,p) = -230.407782

Cartesian coordinates

C 0.560209 0.785919 0.220427

C 1.434474 -0.283047 -0.277531

C 0.226018 -0.979379 0.302832

C -1.142710 -0.941542 -0.223758

C -1.039090 0.355727 0.012434

C -0.429824 1.670591 -0.020598

H 1.590362 -0.271082 -1.364326

H 2.337464 -0.495893 0.294428

H 0.351233 -1.221178 1.364428

H -1.933522 -1.661465 -0.377377

Frequencies

3127.5517

3204.9000

3309.3529

1602.7294

1738.6492

3078.8714

1248.3789

1371.3878

1451.5690

1073.7512

1122.6457

1149.3219

930.4062

976.3346

1052.7455

657.7382

788.7490

827.2552

504.0223

567.9435

627.8480

237.8569

386.4372

416.5962

B-TS84

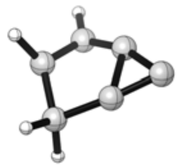

M08HX/6-31+G(d,p) = -230.707019

M08HX/6-31+G(d,p)+ZPVE = -230.636516

CCSD(T)-F12/cc-pVTZ-F12//M08HX/6-31+G(d,p) = -230.411256

Cartesian coordinates

C -0.554482 1.329276 -0.234119

C -1.174635 0.113760 0.267027

C -0.691855 -1.229566 -0.211611

C 0.695531 -0.792307 0.057542

C 0.655656 0.829541 0.014274

C 1.804419 -0.075577 0.095461

H -0.927302 2.339625 -0.344075

H -1.609364 0.123594 1.277766

H -0.830898 -1.459721 -1.279117

H -1.040235 -2.054259 0.413985

Frequencies

3084.2878

3174.3443

3279.2339

1632.7140

1780.6749

3050.0222

1252.7589

1401.0783

1436.7946

1138.4285

1159.0797

1226.2356

902.6642

955.8405

1035.1069

668.0422

774.7702

813.7455

465.0583

503.7137

539.0279

-423.2904

293.3741

380.5771

B-MIN13

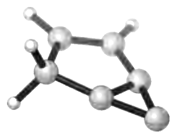

M08HX/6-31+G(d,p) = -230.783358

M08HX/6-31+G(d,p)+ZPVE = -230.70969

CCSD(T)-F12/cc-pVTZ-F12//M08HX/6-31+G(d,p) = -230.479738

Cartesian coordinates

C 0.510429 1.252943 -0.000000

C -0.840903 1.163821 -0.000000

C -1.310852 -0.285772 -0.000000

C -0.000000 -1.004199 0.000000

C 1.025878 -0.118093 0.000000

C 1.327533 -1.527620 0.000000

H 1.095386 2.169316 -0.000000

H -1.525264 2.014064 -0.000000

H -1.921314 -0.534929 -0.880960

H -1.921314 -0.534929 0.880960

Frequencies

3108.3930

3194.8508

3241.2626

1606.2727

1678.6639

3059.9686

1327.9154

1369.0073

1404.4588

1153.9989

1200.2144

1265.8534

928.5173

974.4034

1114.7175

823.6149

909.9489

910.9809

557.8319

753.8502

766.9335

224.7557

325.5311

434.4391

B-TS24

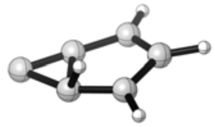

M08HX/6-31+G(d,p) = -230.73542

M08HX/6-31+G(d,p)+ZPVE = -230.665567

CCSD(T)-F12/cc-pVTZ-F12//M08HX/6-31+G(d,p) = -230.430582

Cartesian coordinates

C -0.737070 -0.765522 -0.105642

C 0.651159 -1.117893 -0.062415

C 1.450892 0.085819 0.018759

C 0.612545 1.179331 0.004897

C -0.714023 0.676171 -0.003781

C -1.965912 0.141290 0.015977

H -0.264728 -1.396097 0.902262

H 1.032584 -2.133019 -0.165067

H 2.538797 0.109279 0.077285

H 0.907797 2.224659 -0.021249

Frequencies

3204.7691

3218.2793

3251.0628

1535.5181

1730.3534

2144.2651

1270.6081

1344.7289

1410.0486

1056.6576

1132.7653

1176.9949

953.8248

984.4506

1017.5639

815.2092

821.0905

889.9980

558.8809

622.2966

766.5342

-1201.9316

290.1280

466.0399

B-PR141frag1

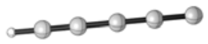

M08HX/6-31+G(d,p) = -230.73542

M08HX/6-31+G(d,p)+ZPVE = -230.665567

CCSD(T)-F12/cc-pVTZ-F12//M08HX/6-31+G(d,p) = -230.430582

Cartesian coordinates

C -5.364165 -0.003276 -0.000681

C -0.217652 0.002112 0.069349

C -1.536522 0.000807 0.046744

C -2.801303 0.001447 0.031030

C -4.141294 -0.000046 0.015351

H -6.434013 -0.001984 -0.035138

Frequencies

146.3013

317.7506

343.3320

523.6256

667.7105

702.7304

769.7585

853.7492

1449.5735

1896.3474

2109.5102

3463.9970

B-PR141frag2

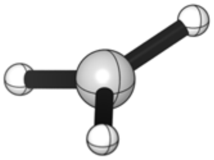

M08HX/6-31+G(d,p) = -230.73542

M08HX/6-31+G(d,p)+ZPVE = -39.798649

CCSD(T)-F12/cc-pVTZ-F12//M08HX/6-31+G(d,p) = -230.430582

Cartesian coordinates

C 0.000000 0.000000 0.000000

H -0.000000 1.083468 -0.000000

H -0.938311 -0.541734 -0.000000

H 0.938311 -0.541734 -0.000000

Frequencies

3147.3835

3331.7291

3331.7567

532.9497

1410.2296

1410.2342

B-MIN53

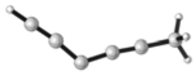

M08HX/6-31+G(d,p) = -230.746062

M08HX/6-31+G(d,p)+ZPVE = -230.677465

CCSD(T)-F12/cc-pVTZ-F12//M08HX/6-31+G(d,p) = -230.451207

Cartesian coordinates

C -3.060961 0.549853 -0.000020

C 2.971997 0.352032 -0.003070

C 1.574437 -0.043894 -0.011640

C 0.384802 -0.352200 -0.003540

C -0.883307 -0.864100 0.003451

C -1.994565 -0.051708 0.001213

H -3.988180 1.084862 -0.001213

H 3.617422 -0.409873 -0.458898

H 3.128996 1.316620 -0.502642

H 3.287339 0.468487 1.044389

Frequencies

3113.9639

3126.4344

3481.0350

2091.5816

2199.5739

3032.8286

1419.0578

1448.2002

1475.9738

1042.6163

1046.8291

1375.6906

722.9828

813.9927

1009.2740

440.6214

443.8755

522.2136

207.9256

334.0090

413.4619

67.7019

93.3195

187.3978

B-TS41

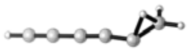

M08HX/6-31+G(d,p) = -230.718666

M08HX/6-31+G(d,p)+ZPVE = -230.651169

CCSD(T)-F12/cc-pVTZ-F12//M08HX/6-31+G(d,p) = -230.423525

Cartesian coordinates

C -3.260081 0.174954 0.014211

C 2.817337 0.464305 -0.029482

C 1.888350 -0.571083 -0.107687

C 0.522871 -0.254139 -0.016833

C -0.693963 -0.132421 -0.012081

C -2.056231 0.029890 0.001704

H -4.323398 0.300101 0.024178

H 2.559026 1.533720 -0.000411

H 2.572471 -0.328796 0.977357

H 3.882208 0.225933 -0.100114

Frequencies

3050.7309

3178.4262

3480.5197

2178.8574

2208.7743

2299.5403

1319.1962

1445.6197

1539.5078

1045.5388

1100.7881

1167.1128

732.3779

752.1926

844.9644

607.0280

667.9294

678.6130

249.1981

323.1706

508.6864

-1165.7141

112.4669

136.6115

B-MIN3

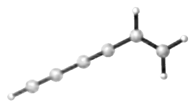

M08HX/6-31+G(d,p) = -230.831737

M08HX/6-31+G(d,p)+ZPVE = -230.758697

CCSD(T)-F12/cc-pVTZ-F12//M08HX/6-31+G(d,p) = -230.539154

Cartesian coordinates

C -1.321057 -2.983629 -0.000000

C 0.462652 1.93436C 0.000000

C 1.758644 2.273791 0.000000

C -0.000000 0.582849 0.000000

C -0.429520 -0.553706 -0.000000

C -0.904418 -1.846665 -0.000000

H -1.690065 -3.988821 -0.000000

H -0.308507 2.708086 0.000000

H 2.057496 3.320808 0.000000

H 2.543272 1.517894 0.000000

Frequencies

3171.3710

3267.0347

3480.9482

2207.4944

2377.5691

3162.3212

1309.6935

1436.5979

1694.9903

1001.2855

1040.3969

1204.6607

808.6279

840.4544

968.1383

654.6624

694.2478

713.0054

350.2117

508.1291

646.9070

107.4311

142.1976

272.4267

B-TS97

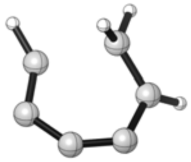

M08HX/6-31+G(d,p) = -230.704067

M08HX/6-31+G(d,p)+ZPVE = -230.633519

CCSD(T)-F12/cc-pVTZ-F12//M08HX/6-31+G(d,p) = -230.419304

Cartesian coordinates

C 1.389778 0.047667 -0.185590

C 0.652149 1.190871 0.197957

C -1.248546 0.812637 -0.159317

C -1.463236 -0.456454 -0.116511

C -0.428513 -1.282911 0.109461

C 0.823507 -1.188159 0.145527

H 2.155535 0.107886 -0.962410

H 0.965119 2.149193 -0.220301

H -1.790450 1.739183 -0.005999

H 0.318961 1.261836 1.239542

Frequencies

3163.1360

3208.7761

3286.3019

1768.8565

1927.5914

3103.9538

1328.8123

1410.5020

1491.5019

1074.1436

1159.2199

1212.3535

878.0978

956.8559

1008.5648

535.7641

705.5525

769.5152

393.8840

475.7056

494.6116

-522.4049

302.1583

311.1461

B-MIN98

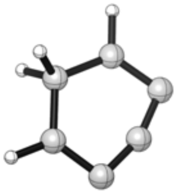

M08HX/6-31+G(d,p) = -230.730029

M08HX/6-31+G(d,p)+ZPVE = -230.658661

CCSD(T)-F12/cc-pVTZ-F12//M08HX/6-31+G(d,p) = -230.435579

Cartesian coordinates

C 0.000000 1.276130 0.441868

C -0.000000 -0.000000 1.228703

C -0.000000 -1.276130 0.441868

C -0.000000 -1.297073 -0.955673

C 0.000000 0.000000 -1.183673

C 0.000000 1.297073 -0.955673

H 0.000000 2.205552 1.026288

H -0.864930 0.000000 1.921451

H -0.000000 -2.205552 1.026288

H 0.864930 -0.000000 1.921451

Frequencies

3032.2198

3108.4420

3109.0614

1474.2736

1737.5996

3000.2360

1321.6390

1351.8869

1372.4166

1127.7724

1263.1899

1310.9021

977.8902

980.4610

1116.8094

681.3918

821.3397

884.4503

523.1344

678.4418

680.7720

202.8770

216.9519

352.9848

B-TS57

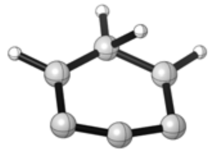

M08HX/6-31+G(d,p) = -230.714946

M08HX/6-31+G(d,p)+ZPVE = -230.645211

CCSD(T)-F12/cc-pVTZ-F12//M08HX/6-31+G(d,p) = -230.419202

Cartesian coordinates

C 1.325640 -0.901078 -0.031925

C 1.217595 0.534111 -0.053530

C -0.100594 1.209405 -0.045814

C -1.303820 0.433685 -0.025447

C -1.233679 -0.979919 0.014084

C 0.055571 -1.203019 0.013011

H 0.522999 0.994128 1.024649

H 2.096853 1.185546 -0.061258

H -0.125896 2.295180 -0.144693

H -2.258228 0.966039 -0.040975

Frequencies

3152.7453

3158.5632

3204.2630

1451.1704

1699.1813

2205.0415

1298.9976

1361.3749

1408.5581

1118.8759

1190.6563

1213.8819

954.2210

1062.6698

1072.9070

773.1983

845.5841

888.4074

458.9350

607.5094

712.0497

-725.6459

378.7470

392.5396

B-MIN66

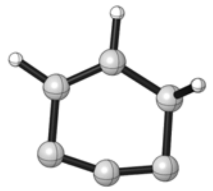

M08HX/6-31+G(d,p) = -230.744437

M08HX/6-31+G(d,p)+ZPVE = -230.673005

CCSD(T)-F12/cc-pVTZ-F12//M08HX/6-31+G(d,p) = -230.448857

Cartesian coordinates

C 1.169608 -1.119590 0.000000

C 1.266812 0.420623 -0.000000

C -0.000000 1.228530 -0.000000

C -1.239618 0.666169 -0.000000

C -1.362792 -0.807552 0.000000

C -0.125442 -1.224081 0.000000

H 1.889920 0.708279 0.866099

H 1.889920 0.708279 -0.866099

H 0.107779 2.316151 -0.000000

H -2.139030 1.282689 -0.000000

Frequencies

3056.7016

3174.6963

3198.0948

1567.0031

1605.3787

3017.0778

1313.0925

1348.5881

1373.2210

1144.6693

1165.8154

1235.8514

962.7367

992.0123

1022.3632

798.3223

865.6897

868.9646

432.3840

669.0257

676.1379

177.8550

308.6191

380.9214

B-TS33

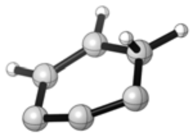

M08HX/6-31+G(d,p) = -230.728284

M08HX/6-31+G(d,p)+ZPVE = -230.658804

CCSD(T)-F12/cc-pVTZ-F12//M08HX/6-31+G(d,p) = -230.433231

Cartesian coordinates

C -1.363681 0.314613 -0.004271

C -0.307113 1.204193 -0.013636

C 1.066170 0.742320 -0.031536

C 1.428089 -0.699974 -0.096541

C 0.184007 -1.215142 -0.015026

C -1.079971 -1.108888 0.022816

H -2.391188 0.685260 0.004938

H -0.491767 2.278571 -0.010903

H 1.870875 1.469463 -0.162162

H 1.447077 0.143978 0.997286

Frequencies

3168.2883

3172.8688

3206.2067

1521.7791

1794.7746

2201.0265

1284.1777

1360.9977

1428.4438

1083.3974

1163.4978

1184.5678

974.9102

993.1550

1029.5368

764.8686

836.2825

872.1061

407.4143

607.2569

684.4355

-864.2803

367.4039

390.8907

B-PR100frag1

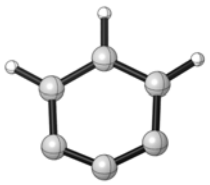

M08HX/6-31+G(d,p) = -230.728284

M08HX/6-31+G(d,p)+ZPVE = -230.105128

CCSD(T)-F12/cc-pVTZ-F12//M08HX/6-31+G(d,p) = -230.433231

Cartesian coordinates

C -1.178936 -0.846490 -0.000000

C -1.239620 0.549538 -0.000000

C -0.000000 1.223001 0.000000

C 1.239624 0.549547 0.000000

C 1.178929 -0.846480 0.000000

C 0.000004 -1.383310 -0.000000

H -2.175946 1.105580 -0.000000

H -0.000005 2.313997 0.000000

H 2.175949 1.105589 0.000000

Frequencies

3189.5101

3209.4402

3214.8511

1426.2351

1486.8323

1592.4292

1175.4049

1306.5821

1369.4392

1037.9602

1119.3706

1131.5116

832.6291

888.3214

968.4819

557.1207

570.3818

765.7453

411.7974

411.9015

439.1501

B-PR100frag2

M08HX/6-31+G(d,p) = -230.728284

M08HX/6-31+G(d,p)+ZPVE = -0.499294

CCSD(T)-F12/cc-pVTZ-F12//M08HX/6-31+G(d,p) = -230.433231

Cartesian coordinates

H 0.000000 0.000000 0.000000

B-PR150frag1

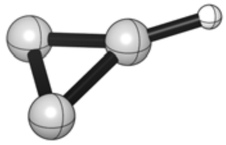

M08HX/6-31+G(d,p) = -230.728284

M08HX/6-31+G(d,p)+ZPVE = -114.629842

CCSD(T)-F12/cc-pVTZ-F12//M08HX/6-31+G(d,p) = -230.433231

Cartesian coordinates

C 0.000000 0.700736 0.000000

C -0.697148 -0.440177 0.000000

C 0.674701 -0.557068 -0.000000

H 0.134679 1.779052 0.000000

Frequencies

1227.9051

1661.9669

3251.9050

678.5972

853.4195

890.4778

B-PR150frag2

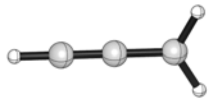

M08HX/6-31+G(d,p) = -230.728284

M08HX/6-31+G(d,p)+ZPVE = -115.928075

CCSD(T)-F12/cc-pVTZ-F12//M08HX/6-31+G(d,p) = -230.433231

Cartesian coordinates

C -0.000000 0.000000 -1.343613

C -0.000000 0.000000 -0.118715

C 0.000000 -0.000000 1.260381

H 0.000000 -0.935082 1.812605

H -0.000000 0.000000 -2.413527

H -0.000000 0.935082 1.812605

Frequencies

3181.8784

3285.2047

3475.1114

1077.5812

1452.7060

2033.9851

663.2973

694.8544

1030.8894

364.4960

421.9269

555.6240

B-MIN19

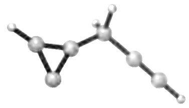

M08HX/6-31+G(d,p) = -230.766309

M08HX/6-31+G(d,p)+ZPVE = -230.695552

CCSD(T)-F12/cc-pVTZ-F12//M08HX/6-31+G(d,p) = -230.465855

Cartesian coordinates

C 0.971179 -0.216007 -0.000000

C 0.340759 -1.539794 0.000000

C -0.178699 -2.627826 0.000000

C -0.235839 2.220849 -0.000000

C -0.000000 0.910558 -0.000000

C -1.352551 1.345095 0.000000

H 1.627139 -0.106386 -0.877443

H 1.627139 -0.106386 0.877443

H -0.652205 -3.588219 0.000000

H 0.128826 3.243744 -0.000000

Frequencies

3084.7896

3256.3309

3482.9118

1798.4920

2277.3028

3041.0895

1299.8621

1369.6624

1424.2577

991.1971

1187.4097

1227.9443

832.2238

922.8346

959.0726

687.3766

701.2734

791.9590

333.6706

371.6123

529.0727

45.8148

123.9843

318.3091

B-TS258

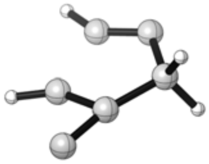

M08HX/6-31+G(d,p) = -230.664153

M08HX/6-31+G(d,p)+ZPVE = -230.59632

CCSD(T)-F12/cc-pVTZ-F12//M08HX/6-31+G(d,p) = -230.369125

Cartesian coordinates

C 0.601078 -1.216137 -0.000031

C 1.597664 -0.138605 0.000088

C 1.143583 1.063323 0.000032

C -0.742137 0.893942 -0.000098

C -0.768716 -0.473607 -0.000101

C -2.064497 -0.161847 0.000142

H 0.642803 -1.868257 0.884289

H 0.642957 -1.868168 -0.884410

H 1.412444 2.111147 0.000010

H -1.300058 1.822857 -0.000084

Frequencies

3118.4859

3304.9282

3327.3181

1708.8400

1737.9108

3068.1106

1219.3201

1316.9583

1423.5358

1004.8964

1073.6278

1153.4483

795.9216

844.9396

905.9254

679.7457

696.3161

748.3870

332.8941

427.7268

548.0744

-804.8192

35.9303

302.0639

B-MIN133

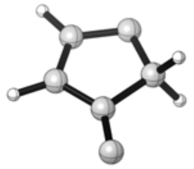

M08HX/6-31+G(d,p) = -230.715518

M08HX/6-31+G(d,p)+ZPVE = -230.644844

CCSD(T)-F12/cc-pVTZ-F12//M08HX/6-31+G(d,p) = -230.417869

Cartesian coordinates

C 1.202015 0.123556 0.000000

C 0.588250 1.480394 -0.000000

C -0.830897 1.268085 -0.000000

C -1.178065 -0.065142 -0.000000

C 0.230735 -2.178481 0.000000

C -0.000000 -0.892401 0.000000

H 1.842619 -0.006898 -0.883631

H 1.842619 -0.006898 0.883631

H -1.566960 2.070808 -0.000000

H -2.190497 -0.473084 -0.000000

Frequencies

3129.2909

3194.2427

3221.6656

1485.1198

1793.0024

3074.1754

1235.0988

1374.3447

1385.3345

1105.0679

1110.5541

1162.6289

942.1449

997.3960

1020.2929

786.3688

807.2673

858.2319

419.5592

627.5373

757.8451

111.4641

141.9022

281.9368

B-TS116

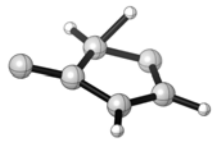

M08HX/6-31+G(d,p) = -230.696235

M08HX/6-31+G(d,p)+ZPVE = -230.62812

CCSD(T)-F12/cc-pVTZ-F12//M08HX/6-31+G(d,p) = -230.399234

Cartesian coordinates

C 0.297726 -1.197980 0.001472

C 1.427258 -0.441527 0.001591

C 1.158269 1.008230 -0.107388

C -0.254886 1.105284 -0.031901

C -0.851422 -0.312937 -0.009752

C -2.151229 -0.205843 0.003452

H 0.217116 -2.282138 0.037568

H 2.434452 -0.852339 0.032222

H 0.432769 1.394892 0.981623

H -0.838631 2.008228 -0.196256

Frequencies

3221.8111

3237.8657

3244.7023

1564.6552

1832.8280

2218.5904

1290.3662

1316.3557

1361.7641

1035.1657

1116.7716

1176.6848

872.9693

970.6981

1002.0685

776.3079

817.3827

839.0303

501.2706

548.0828

667.5569

-894.2195

104.7552

181.4885

B-TS1

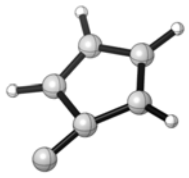

M08HX/6-31+G(d,p) = -230.810478

M08HX/6-31+G(d,p)+ZPVE = -230.737646

CCSD(T)-F12/cc-pVTZ-F12//M08HX/6-31+G(d,p) = -230.511659

Cartesian coordinates

C -1.113987 0.121687 0.000000

C -0.457523 1.312047 0.000000

C 0.981052 1.105590 -0.000000

C 1.259792 -0.226992 -0.000000

C -0.000000 -0.930678 0.000000

C -0.804180 -1.950139 0.000000

H -2.183983 -0.050959 0.000000

H -0.955731 2.278368 0.000000

H 1.722471 1.901326 -0.000000

H 2.226316 -0.717826 -0.000000

Frequencies

3243.0964

3279.2899

3282.4467

1612.5334

1879.7045

3230.0467

1284.6355

1403.3629

1512.4838

1044.2524

1114.8023

1118.6735

917.8183

949.2793

1000.2010

762.2926

821.8355

870.5951

576.6233

684.3720

685.9724

-125.3067

146.7510

548.4938

B-PR108frag1

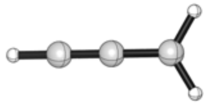

M08HX/6-31+G(d,p) = -230.810478

M08HX/6-31+G(d,p)+ZPVE = -115.928075

CCSD(T)-F12/cc-pVTZ-F12//M08HX/6-31+G(d,p) = -230.511659

Cartesian coordinates

C -0.000000 0.000000 -1.343613

C -0.000000 0.000000 -0.118715

C 0.000000 -0.000000 1.260381

H 0.000000 -0.935082 1.812605

H -0.000000 0.000000 -2.413527

H -0.000000 0.935082 1.812605

Frequencies

3181.8784

3285.2047

3475.1114

1077.5812

1452.7060

2033.9851

663.2973

694.8544

1030.8894

364.4960

421.9269

555.6240

B-PR108frag2

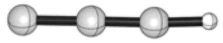

M08HX/6-31+G(d,p) = -230.810478

M08HX/6-31+G(d,p)+ZPVE = -114.622091

CCSD(T)-F12/cc-pVTZ-F12//M08HX/6-31+G(d,p) = -230.511659

Cartesian coordinates

C -0.004925 -0.025327 -1.436098

C -0.000342 -0.002016 -0.088245

C 0.003988 0.014479 1.153659

H 0.007672 0.077184 2.224107

Frequencies

1162.2537

1901.0751

3453.7308

240.6887

330.7791

384.0972

B-MIN36

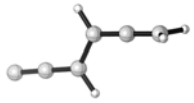

M08HX/6-31+G(d,p) = -230.754396

M08HX/6-31+G(d,p)+ZPVE = -230.684083

CCSD(T)-F12/cc-pVTZ-F12//M08HX/6-31+G(d,p) = -230.456045

Cartesian coordinates

C -0.340883 -0.753354 -0.000000

C 0.000000 0.655208 0.000000

C 1.265463 1.046527 0.000000

C 2.510623 1.422959 0.000000

C -1.600599 -1.216818 -0.000000

C -2.797954 -1.671136 -0.000000

H 0.498299 -1.459657 0.000000

H -0.807518 1.388856 -0.000000

H 3.044657 1.585242 0.937241

H 3.044657 1.585242 -0.937241

Frequencies

3145.8243

3189.5799

3233.5425

2023.5103

2090.0735

3128.7776

1278.5118

1408.9646

1505.6459

1076.8521

1134.6357

1203.3520

913.8675

938.0415

985.5531

546.9087

547.4749

891.8717

280.3725

402.6112

503.9996

90.9391

120.6833

222.1330

B-TS75

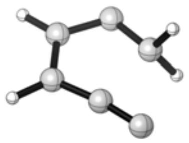

M08HX/6-31+G(d,p) = -230.708961

M08HX/6-31+G(d,p)+ZPVE = -230.639291

CCSD(T)-F12/cc-pVTZ-F12//M08HX/6-31+G(d,p) = -230.415371

Cartesian coordinates

C 1.190548 0.487675 -0.000000

C 0.209661 1.491512 -0.000000

C -1.107937 1.035681 0.000000

C -1.204280 -0.364795 0.000000

C 0.000000 -1.059346 0.000000

C 1.005014 -1.850672 0.000000

H 1.793156 0.393885 -0.910339

H 1.793156 0.393885 0.910339

H -1.988952 1.674606 0.000000

H -2.155392 -0.902710 0.000000

Frequencies

3184.7310

3193.7682

3223.2189

1474.0731

2004.8260

3114.6488

1318.1705

1370.5293

1426.4928

1039.1180

1046.3178

1115.3766

969.4400

980.7601

1023.7280

692.8292

753.3939

888.3780

321.4510

458.3671

621.8953

-417.4892

157.1693

202.7297

B-PR109frag1

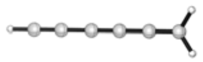

M08HX/6-31+G(d,p) = -230.708961

M08HX/6-31+G(d,p)+ZPVE = -230.107588

CCSD(T)-F12/cc-pVTZ-F12//M08HX/6-31+G(d,p) = -230.415371

Cartesian coordinates

C -0.000000 -0.000000 0.538210

C 0.000000 0.000000 -0.712397

C -0.000000 -0.000000 1.847357

C 0.000000 0.000000 -2.063652

C -0.000000 -0.000000 3.157878

C 0.000000 0.000000 -3.284081

H -0.000000 0.933219 3.727431

H 0.000000 -0.933219 3.727431

H 0.000000 0.000000 -4.354753

Frequencies

3106.8730

3187.3493

3473.3765

1878.1007

1977.8824

2144.8862

978.1893

1191.1269

1433.0512

777.2078

850.0564

897.0117

594.5901

638.1123

682.7180

290.6801

425.2014

555.0342

104.9158

115.5092

214.5517

B-PR109frag2

M08HX/6-31+G(d,p) = -230.708961

M08HX/6-31+G(d,p)+ZPVE = -0.499294

CCSD(T)-F12/cc-pVTZ-F12//M08HX/6-31+G(d,p) = -230.415371

Cartesian coordinates

H 0.000000 0.000000 0.000000

B-MIN71

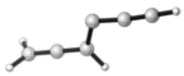

M08HX/6-31+G(d,p) = -230.740161

M08HX/6-31+G(d,p)+ZPVE = -230.671579

CCSD(T)-F12/cc-pVTZ-F12//M08HX/6-31+G(d,p) = -230.442449

Cartesian coordinates

C 2.438702 1.898683 0.000000

C -2.392695 -1.733079 -0.000000

C -1.213297 -1.192557 -0.000000

C 0.000000 -0.636166 -0.000000

C 1.356669 1.327100 0.000000

C 0.068898 0.801512 0.000000

H 3.375810 2.416689 0.000000

H -2.900313 -1.955424 -0.938689

H -2.900313 -1.955424 0.938689

H 0.875161 -1.298800 -0.000000

Frequencies

3156.6343

3245.4064

3477.7762

2015.5740

2101.0915

3099.6510

1234.7280

1363.9769

1430.9853

973.7238

974.0975

1100.6812

808.7283

865.3419

924.1534

545.4957

555.7104

560.6016

327.5247

433.4662

472.0879

68.3796

124.1764

243.8664

B-TS36

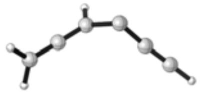

M08HX/6-31+G(d,p) = -230.721041

M08HX/6-31+G(d,p)+ZPVE = -230.653876

CCSD(T)-F12/cc-pVTZ-F12//M08HX/6-31+G(d,p) = -230.424516

Cartesian coordinates

C -0.497977 0.760543 -0.315170

C -1.577592 0.045333 -0.116575

C -2.665357 -0.648357 0.099737

C 2.769562 -0.677857 -0.103315

C 1.768608 -0.004446 0.118205

C 0.722054 0.801812 0.490610

H -3.451058 -0.273960 0.758841

H -2.821033 -1.620284 -0.371586

H 3.641658 -1.268018 -0.299430

H -0.485356 1.500092 -1.128776

Frequencies

3131.8840

3214.6131

3468.3977

2060.8696

2095.5302

3092.9499

1156.2108

1354.2045

1485.7385

910.0612

1020.6497

1085.7186

820.3664

833.9542

850.0793

471.5516

546.4718

628.7358

220.6203

309.5563

422.1195

-379.0885

107.3597

194.1774

B-MIN74

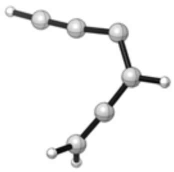

M08HX/6-31+G(d,p) = -230.738255

M08HX/6-31+G(d,p)+ZPVE = -230.669899

CCSD(T)-F12/cc-pVTZ-F12//M08HX/6-31+G(d,p) = -230.441497

Cartesian coordinates

C 1.180606 -0.719578 0.000000

C 1.280615 0.725069 0.000000

C 0.000000 -1.337251 0.000000

C 0.093815 1.445371 -0.000000

C -1.184404 -1.871468 -0.000000

C -0.872997 2.195960 -0.000000

H -1.691122 -2.095467 0.938987

H 2.095543 -1.314630 0.000000

H -1.691122 -2.095467 -0.938987

H -1.699105 2.876949 -0.000000

Frequencies

3172.0572

3243.9210

3472.7211

2000.4001

2091.4584

3154.6068

1287.7379

1351.9186

1421.4345

922.7031

971.9984

1083.1680

801.8968

854.7420

897.0970

465.0811

503.3402

690.8309

313.5683

439.3879

450.9503

62.9394

103.4886

247.4533

B-TS92

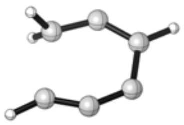

M08HX/6-31+G(d,p) = -230.702797

M08HX/6-31+G(d,p)+ZPVE = -230.634551

CCSD(T)-F12/cc-pVTZ-F12//M08HX/6-31+G(d,p) = -230.415357

Cartesian coordinates

C 1.406618 0.619706 -0.000000

C 0.233968 1.322065 0.000000

C -1.083525 0.968205 0.000000

C -1.251327 -1.091773 0.000000

C 0.000000 -1.32527H -0.000000

C 1.212724 -0.795737 -0.000000

H 2.398793 1.065098 -0.000000

H -1.646299 1.167482 -0.918352

H -1.646299 1.167482 0.918352

H -2.216945 -1.583235 0.000000

Frequencies

3190.3468

3211.3258

3307.1712

1646.4862

1885.0182

3112.7404

1280.4833

1350.8465

1430.8490

970.6071

990.8130

1055.7377

801.2650

890.8044

927.8587

564.3135

624.7563

751.7690

415.3341

499.2427

509.7059

-481.0537

234.3431

304.9908

B-MIN106

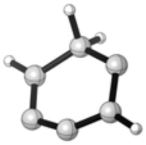

M08HX/6-31+G(d,p) = -230.727307

M08HX/6-31+G(d,p)+ZPVE = -230.656073

CCSD(T)-F12/cc-pVTZ-F12//M08HX/6-31+G(d,p) = -230.415357

Cartesian coordinates

C 1.303160 -0.872702 0.000000

C 1.270521 0.545514 0.000000

C -0.007234 1.274537 0.000000

C -1.352188 0.531483 -0.000000

C -1.206969 -0.808565 -0.000000

C 0.000000 -1.265116 -0.000000

H 2.204247 -1.479551 0.000000

H 0.020013 1.974342 -0.858771

H 0.020013 1.974342 0.858771

H -2.288009 1.099960 -0.000000

Frequencies

3034.4891

3149.2122

3232.9806

1532.9949

1917.7816

3002.7423

1264.3816

1312.3356

1372.6979

1125.5038

1156.6977

1248.0106

892.9094

928.6040

1066.8308

713.1033

829.7195

845.6622

556.8045

562.2882

651.5879

155.7697

285.9149

429.2253

B-TS44

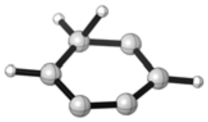

M08HX/6-31+G(d,p) = -230.720347

M08HX/6-31+G(d,p)+ZPVE = -230.65092

CCSD(T)-F12/cc-pVTZ-F12//M08HX/6-31+G(d,p) = -230.429554

Cartesian coordinates

C -1.448088 -0.179847 -0.019595

C -0.639894 1.072869 -0.030246

C 0.799609 1.136975 -0.096427

C 1.525011 -0.122853 -0.003574

C 0.619053 -1.123443 0.009932

C -0.653382 -1.278526 0.002692

H -2.539089 -0.140418 -0.014287

H -1.178574 2.008853 -0.201243

H -0.106152 1.279323 1.031693

H 2.609965 -0.178813 0.007153

Frequencies

3164.4344

3185.9066

3235.7521

1524.4977

1943.1899

2357.8327

1260.2222

1325.0152

1365.3421

1118.2418

1191.1911

1222.8730

903.4843

923.5500

1018.6231

663.9670

769.0263

852.8016

510.4575

582.3612

597.0658

-654.6532

309.3637

449.6292

B-PR213frag1

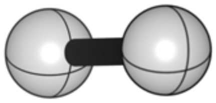

M08HX/6-31+G(d,p) = -230.720347

M08HX/6-31+G(d,p)+ZPVE = -75.896281

CCSD(T)-F12/cc-pVTZ-F12//M08HX/6-31+G(d,p) = -75.806358

Cartesian coordinates

C 0.000000 0.000000 0.625620

C 0.000000 0.000000 -0.625620

Frequencies

1872.8086

B-PR213frag2

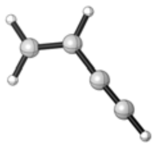

M08HX/6-31+G(d,p) = -230.720347

M08HX/6-31+G(d,p)+ZPVE = -154.71989

CCSD(T)-F12/cc-pVTZ-F12//M08HX/6-31+G(d,p) = -154.501638

Cartesian coordinates

C 1.906826 -0.171374 0.000000

C 0.735430 0.109362 0.000000

C -0.635693 0.488342 0.000000

C -1.659933 -0.371007 0.000000

H -1.501797 -1.442895 0.000000

H -2.683070 -0.015452 0.000000

H 2.936752 -0.431470 0.000251

H -0.831662 1.557875 0.000000

Frequencies

3147.6204

3236.4459

3476.3173

1668.6693

2205.5866

3136.3973

1111.5143

1320.9794

1443.5487

892.2780

954.7872

1010.0495

647.7072

680.3402

703.5328

224.3396

316.4100

557.6134

B-MIN157

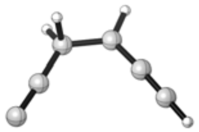

M08HX/6-31+G(d,p) = -230.720347

M08HX/6-31+G(d,p)+ZPVE = -230.722465

CCSD(T)-F12/cc-pVTZ-F12//M08HX/6-31+G(d,p) = -154.501638

Cartesian coordinates

C 1.479749 -0.000635 -0.009175

C 2.180300 -0.991859 0.016743

C 0.633268 1.080920 -0.070214

C -0.821808 0.961682 0.011440

C -1.536312 -0.265132 -0.018152

C -2.208770 -1.334059 -0.025612

H 1.054635 2.081559 -0.089274

H 2.787224 -1.865459 0.041363

H -0.886719 1.265350 1.098980

H -1.313697 1.813040 -0.481246

Frequencies

3001.1290

3166.4599

3460.8348

1998.7401

2139.2584

2767.7873

1226.4100

1332.2833

1406.9663

876.2117

1040.4077

1172.0448

719.2314

750.3722

815.0754

515.7628

642.5051

700.8577

204.8866

339.0739

402.2745

83.3883

143.5638

178.1710

B-TS201

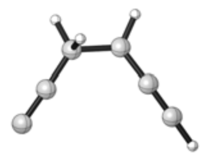

M08HX/6-31+G(d,p) = -230.720347

M08HX/6-31+G(d,p)+ZPVE = -230.722633

CCSD(T)-F12/cc-pVTZ-F12//M08HX/6-31+G(d,p) = -154.501638

Cartesian coordinates

C 1.481616 -0.011595 -0.009636

C 2.213700 -0.975164 0.009591

C 0.618388 1.069103 -0.076916

C -0.832799 0.944773 -0.012157

C -1.560145 -0.252540 -0.037221

C -2.246611 -1.311406 -0.005486

H 1.028765 2.074231 -0.063320

H 2.847247 -1.829636 0.026421

H -0.570610 1.106517 1.113647

H -1.350300 1.869862 -0.285800

Frequencies

3051.4953

3168.3758

3463.5244

2026.9436

2162.8405

2521.5858

1206.3108

1388.4012

1399.9093

879.5212

1054.1704

1196.6624

715.5315

744.9184

809.5401

485.6343

650.0548

693.8556

207.9793

269.4524

401.3278

-527.1223

88.9015

162.3430

B-MIN55

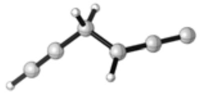

M08HX/6-31+G(d,p) = -230.720347

M08HX/6-31+G(d,p)+ZPVE = -230.785704

CCSD(T)-F12/cc-pVTZ-F12//M08HX/6-31+G(d,p) = -154.501638

Cartesian coordinates

C -2.778745 -0.461518 -0.206691

C -1.727136 0.106335 -0.096553

C -0.432827 0.768812 0.039324

C 0.646963 -0.169713 0.517238

C 3.073666 -0.217820 -0.438250

C 1.883673 -0.194645 0.034378

H -3.713696 -0.956045 -0.310704

H -0.127509 1.241670 -0.896890

H -0.513252 1.572344 0.787835

H 0.360894 -0.846669 1.323086

Frequencies

3081.4040

3111.4156

3474.4270

2043.7683

2230.6367

2983.9554

1295.9055

1424.3582

1443.8660

1032.5771

1174.4207

1213.7500

743.2715

956.8236

973.4109

503.4694

679.0909

703.3660

235.2557

327.0935

486.5516

47.3262

148.7009

199.3520

B-TS304

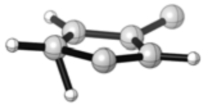

M08HX/6-31+G(d,p) = -230.720347

M08HX/6-31+G(d,p)+ZPVE = -230.703368

CCSD(T)-F12/cc-pVTZ-F12//M08HX/6-31+G(d,p) = -154.501638

Cartesian coordinates

C -0.170911 -1.190849 0.024832

C -1.385007 -0.486923 -0.073205

C -1.147824 1.034002 -0.124100

C 0.207340 1.180041 0.042630

C 0.855481 -0.215715 0.132879

C 2.144826 -0.164984 -0.109597

H -0.008323 -2.257533 -0.040678

H -2.352699 -0.937924 -0.249804

H -1.455198 0.179169 0.928592

H 0.792788 2.082858 0.001262

Frequencies

3208.0614

3229.0322

3268.5303

1416.7127

1733.3861

2266.0537

1196.0044

1282.2844

1367.4363

1034.1962

1074.6880

1124.7651

801.2713

844.9451

858.9901

674.8465

701.5057

760.5354

530.9388

550.6317

661.7662

-841.7307

137.9384

198.4834

B-TS228

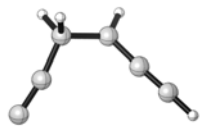

M08HX/6-31+G(d,p) = -230.720347

M08HX/6-31+G(d,p)+ZPVE = -230.717671

CCSD(T)-F12/cc-pVTZ-F12//M08HX/6-31+G(d,p) = -154.501638

Cartesian coordinates

C 2.295703 -0.867642 0.095422

C -1.494860 -0.330334 0.083000

C -2.216268 -1.317780 -0.191309

C -0.856123 0.965408 0.223675

C 0.545121 1.011844 -0.233509

C 1.454354 -0.004090 -0.059647

H 3.011060 -1.640652 0.246578

H 0.883510 1.878571 -0.797564

H -0.831967 1.247598 1.297737

H -1.430156 1.770047 -0.252549

Frequencies

3026.7386

3142.2619

3460.0159

1897.8007

2126.0718

2883.6155

1284.9238

1341.4995

1405.3454

994.4451

1086.3864

1166.1968

737.3236

806.1837

888.9197

460.7913

646.2517

687.9884

220.1067

272.1325

369.7155

-458.0081

103.3376

202.1192

B-MIN68

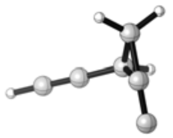

M08HX/6-31+G(d,p) = -230.720347

M08HX/6-31+G(d,p)+ZPVE = -230.778367

CCSD(T)-F12/cc-pVTZ-F12//M08HX/6-31+G(d,p) = -154.501638

Cartesian coordinates

C 0.062610 -0.278727 0.584426

C -1.263478 0.005600 0.139700

C -2.397140 0.209470 -0.203603

C 0.972620 -1.156235 -0.234751

C 1.326983 0.254711 -0.270550

C 1.458810 1.504833 -0.066369

H 0.234656 -0.254213 1.653534

H -3.393993 0.406830 -0.514597

H 1.647452 -1.811297 0.308473

H 0.549464 -1.579239 -1.140527

Frequencies

3175.2027

3189.7104

3475.1393

1935.8170

2221.5170

3104.2024

1154.8137

1357.8505

1474.7699

1050.8958

1107.5075

1112.2592

780.0145

898.0421

968.0959

663.0182

684.3358

708.2727

276.4016

485.3557

522.5393

119.2739

166.7603

203.9413

B-TS401

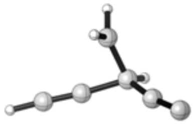

M08HX/6-31+G(d,p) = -230.720347

M08HX/6-31+G(d,p)+ZPVE = -230.685926

CCSD(T)-F12/cc-pVTZ-F12//M08HX/6-31+G(d,p) = -154.501638

Cartesian coordinates

C 2.201934 -0.760365 -0.185737

C 1.117557 -0.353169 0.175569

C 0.254560 1.362565 -0.275343

C -0.192074 0.240989 0.553751

C -1.417164 -0.326086 0.039829

C -2.584437 -0.640405 -0.296429

H 3.141453 -1.153919 -0.493844

H 0.121919 1.272464 -1.345270

H 0.636989 2.290630 0.141406

H -0.182610 0.449652 1.627867

Frequencies

3127.5944

3270.4296

3459.0334

1954.0173

2103.3593

3049.2273

1186.5693

1289.0831

1465.7248

989.4810

1004.1487

1151.5538

676.5529

796.2622

889.6402

499.9118

649.9680

662.2570

214.0526

269.5965

448.8703

-609.3232

102.6870

135.0534

C-PR95frag1

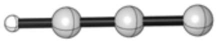

M08HX/6-31+G(d,p) = -230.720347

M08HX/6-31+G(d,p)+ZPVE = -114.62137

CCSD(T)-F12/cc-pVTZ-F12//M08HX/6-31+G(d,p) = -154.501638

Cartesian coordinates

C 1.929356 -0.256006 0.022264

C 2.871665 0.328672 -0.744695

C 1.061707 -0.794363 0.729023

H 0.312214 -1.259260 1.338840

Frequencies

1160.0133

1900.3043

3456.2901

241.2476

375.7438

658.5500

C-PR95frag2

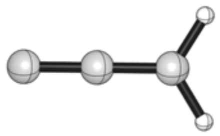

M08HX/6-31+G(d,p) = -115.300224

M08HX/6-31+G(d,p)+ZPVE = -115.269005

CCSD(T)-F12/cc-pVTZ-F12//M08HX/6-31+G(d,p) = -115.154272

Cartesian coordinates

C -0.000000 -0.000000 1.125066

C 0.000000 0.000000 -0.199706

C 0.000000 0.000000 -1.490224

H -0.000000 -0.933809 1.694592

H 0.000000 0.933809 1.694592

Frequencies

2075.0602

3109.8600

3201.6399

1044.9847

1166.7800

1474.6029

283.6144

302.8405

1044.4021

C-TS88

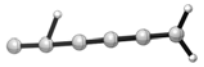

M08HX/6-31+G(d,p) = -115.300224

M08HX/6-31+G(d,p)+ZPVE = -229.98382

CCSD(T)-F12/cc-pVTZ-F12//M08HX/6-31+G(d,p) = -115.154272

Cartesian coordinates

C -0.242998 3.419840 -0.000000

C -0.194650 2.121659 -0.000000

C -0.103684 0.773315 -0.000000

C 0.000000 -0.497337 0.000000

C 0.099269 -1.781964 0.000000

C 0.200682 -3.090071 0.000000

H -0.689465 -3.721206 0.000000

H 0.961344 1.627201 -0.000000

H 1.176406 -3.578649 -0.000000

Frequencies

2264.6411

3136.5002

3224.9785

1750.3181

1942.9995

2105.4712

989.7149

1189.1519

1427.5351

560.2417

643.2238

883.3642

410.6655

471.6710

480.8274

242.2581

274.6845

382.0029

-1109.9806

110.0519

122.7284

C-MIN2

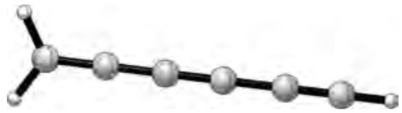

M08HX/6-31+G(d,p) = -115.300224

M08HX/6-31+G(d,p)+ZPVE = -230.107588

CCSD(T)-F12/cc-pVTZ-F12//M08HX/6-31+G(d,p) = -115.154272

Cartesian coordinates

C 0.000181 -3.157878 -0.000000

C 0.000074 -1.847357 -0.000000

C -0.000000 -0.538210 -0.000000

C -0.000068 0.712396 0.000000

C -0.000121 2.063652 0.000000

C -0.000122 3.284081 0.000000

H 0.933454 -3.727346 -0.000000

H -0.932995 -3.727503 -0.000000

H -0.000129 4.354752 0.000000

Frequencies

3106.8548

3187.3348

3473.3771

1878.1010

1977.8827

2144.8863

978.1971

1191.1266

1433.0502

777.2075

850.0561

897.0145

594.5896

638.1123

682.7174

290.6794

425.2014

555.0335

104.9184

115.5083

214.5547

C-PR3frag1

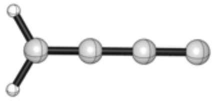

M08HX/6-31+G(d,p) = -153.3749

M08HX/6-31+G(d,p)+ZPVE = -153.338821

CCSD(T)-F12/cc-pVTZ-F12//M08HX/6-31+G(d,p) = -153.17851

Cartesian coordinates

C -0.000000 0.000000 -1.755069

C -0.000000 0.000000 -0.451617

C 0.000000 -0.000000 2.130613

C 0.000000 -0.000000 0.844643

H -0.000000 -0.940212 -2.305711

H 0.000000 0.940212 -2.305711

Frequencies

2191.4782

3162.3392

3254.0535

955.2939

1366.5295

1784.0513

550.3972

811.2957

941.0276

184.4197

205.3987

430.5479

C-PR116frag1

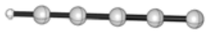

M08HX/6-31+G(d,p) = -153.3749

M08HX/6-31+G(d,p)+ZPVE = -190.752731

CCSD(T)-F12/cc-pVTZ-F12//M08HX/6-31+G(d,p) = -153.17851

Cartesian coordinates

C 0.000000 -0.000000 -1.392561

C -0.000000 0.000000 2.435329

C -0.000000 0.000000 1.212373

C 0.000000 -0.000000 -0.127811

C 0.000000 -0.000000 -2.711870

H -0.000000 0.000000 3.507242

Frequencies

3461.6365

1450.0001

1896.6301

2109.0123

703.6499

769.8343

856.2745

345.6125

528.3580

669.9795

147.6881

152.9553

323.1472

C-PR116frag2

UM08HX/6-31+G(d,p) = -39.127031

UM08HX/6-31+G(d,p)+ZPVE = -39.107896

UCCSD(T)-F12/cc-pVTZ-F12//M08HX/6-31+G(d,p) = -39.075011

Cartesian coordinates

C 0.000000 0.000000 -0.176097

H 0.000000 0.866702 0.528290

H 0.000000 -0.866702 0.528290

Frequencies

1402.9010

2943.0663

3027.2733

C-MIN25

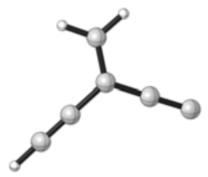

M08HX/6-31+G(d,p) = -39.124693

M08HX/6-31+G(d,p)+ZPVE = -230.044193

CCSD(T)-F12/cc-pVTZ-F12//M08HX/6-31+G(d,p) = -39.075012

Cartesian coordinates

C -0.000000 0.372628 -0.000000

C -0.278838 1.721892 -0.000000

C 2.557087 -0.427491 -0.000000

C 1.330765 -0.055039 -0.000000

C -1.067521 -0.595832 0.000000

C -1.956703 -1.412669 0.000000

H 0.538855 2.439041 -0.000000

H -1.305999 2.082077 -0.000000

H -2.741596 -2.142046 0.000000

Frequencies

3167.2171

3288.8208

3480.4502

1519.7453

2024.1885

2258.8758

980.3116

1251.8594

1365.8918

710.2382

769.1773

963.6910

577.2345

663.5264

709.2097

309.1206

478.2397

548.3015

132.9142

189.3266

231.8452

C-TS148

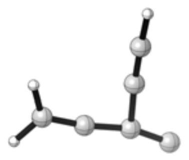

M08HX/6-31+G(d,p) = -39.124693

M08HX/6-31+G(d,p)+ZPVE = -229.963033

CCSD(T)-F12/cc-pVTZ-F12//M08HX/6-31+G(d,p) = -39.075012

Cartesian coordinates

C -2.079190 0.069631 0.000005

C -0.893026 -0.478673 -0.000006

C 0.430752 -0.878427 -0.000008

C 1.531721 -1.604501 0.000009

C 0.684152 0.571584 0.000001

C 0.975785 1.757758 -0.000002

H -2.211598 1.154614 0.000057

H -2.970150 -0.562668 -0.000038

H 1.280580 2.783824 -0.000015

Frequencies

3112.2257

3200.6528

3478.4840

1648.0279

1927.3305

2076.4065

885.6286

970.1110

1393.0050

645.6583

833.4651

883.3085

412.5981

518.5760

550.4273

182.9551

326.6348

368.3439

-593.4459

148.6930

163.3458

C-PR26frag1

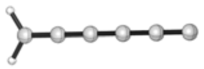

M08HX/6-31+G(d,p) = -229.50211

M08HX/6-31+G(d,p)+ZPVE = -229.455408

CCSD(T)-F12/cc-pVTZ-F12//M08HX/6-31+G(d,p) = -229.208741

Cartesian coordinates

C -0.000000 -0.000000 -3.031889

C 0.000000 0.000000 0.834905

C -0.000000 -0.000000 -0.438404

C 0.000000 0.000000 2.134541

C 0.000000 0.000000 3.419825

C -0.000000 -0.000000 -1.723537

H 0.000000 -0.938619 -3.586322

H 0.000000 0.938619 -3.586322

Frequencies

2232.8533

3153.3655

3245.0143

1408.9636

1809.0213

2140.3645

839.5725

966.8333

1234.4409

577.7678

649.0720

667.5494

242.0500

399.9256

498.7688

101.6391

103.5700

229.0405

C-PR26frag2

M08HX/6-31+G(d,p) = -229.50211

M08HX/6-31+G(d,p)+ZPVE = -0.499294

CCSD(T)-F12/cc-pVTZ-F12//M08HX/6-31+G(d,p) = -229.208741

Cartesian coordinates

H 0.000000 0.000000 0.000000

C-MIN14

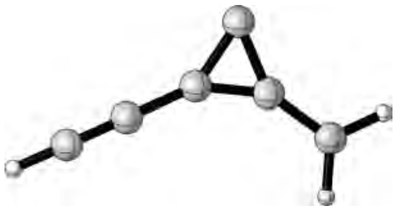

M08HX/6-31+G(d,p) = -229.50211

M08HX/6-31+G(d,p)+ZPVE = -230.059858

CCSD(T)-F12/cc-pVTZ-F12//M08HX/6-31+G(d,p) = -229.208741

Cartesian coordinates

C 1.147043 0.079381 0.000002

C -2.662006 -0.431965 -0.000013

C -1.509110 -0.062972 -0.000000

C -0.175928 0.364587 0.000013

C 0.750919 1.451642 0.000011

C 2.194140 -0.866179 -0.000006

H 1.989105 -1.933121 0.000153

H -3.685001 -0.750402 -0.000023

H 3.225545 -0.523436 -0.000174

Frequencies

3171.4566

3286.1739

3478.9824

1467.4280

1773.2095

2233.4755

1046.9766

1301.3611

1329.6456

761.0560

795.1112

854.9626

611.9855

671.5144

708.4383

337.7367

397.0663

557.9933

143.4198

150.1007

229.5479

C-TS53

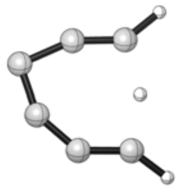

M08HX/6-31+G(d,p) = -229.50211

M08HX/6-31+G(d,p)+ZPVE = -229.995838

CCSD(T)-F12/cc-pVTZ-F12//M08HX/6-31+G(d,p) = -229.208741

Cartesian coordinates

C -0.783996 1.456719 -0.000011

C 1.745441 0.478658 0.000008

C 1.216493 -0.683901 -0.000017

C 0.017011 -1.229714 -0.000018

C -1.281393 -1.101371 0.000030

C -1.283535 0.292069 -0.000002

H -1.064389 2.498210 0.000015

H 2.699124 0.990708 0.000091

H 0.585129 1.236320 -0.000046

Frequencies

2035.6303

3314.7427

3363.8189

1401.0511

1745.4556

1909.2661

989.6741

1087.2071

1138.2869

636.1322

858.7881

888.7323

525.7774

554.9689

609.3308

462.8146

481.0740

503.9553

-1259.5288

273.0969

384.0198

C-PR51frag1

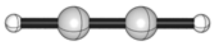

M08HX/6-31+G(d,p) = -77.310021

M08HX/6-31+G(d,p)+ZPVE = -77.282823

CCSD(T)-F12/cc-pVTZ-F12//M08HX/6-31+G(d,p) = -77.216169

Cartesian coordinates

C -0.000000 0.000000 0.602947

C 0.000000 0.000000 -0.602947

H -0.000000 0.000000 1.673947

H 0.000000 0.000000 -1.673947

Frequencies

3531.2652

769.8168

2098.0711

3426.5684

671.7108

671.7108

769.8168

C-PR51frag2

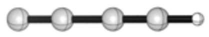

M08HX/6-31+G(d,p) = -77.310021

M08HX/6-31+G(d,p)+ZPVE = -152.688229

CCSD(T)-F12/cc-pVTZ-F12//M08HX/6-31+G(d,p) = -77.216169

Cartesian coordinates

C 0.000000 0.000000 1.781412

C 0.000000 0.000000 0.571927

C -0.000000 -0.000000 -0.808519

C -0.000000 -0.000000 -2.020246

H 0.000000 0.000000 2.852556

Frequencies

3477.8145

930.5940

2171.8034

2335.1772

553.1311

737.2823

737.2823

218.2670

218.2670

553.1311

C-PR25frag1

M08HX/6-31+G(d,p) = -115.300224

M08HX/6-31+G(d,p)+ZPVE = -115.269005

CCSD(T)-F12/cc-pVTZ-F12//M08HX/6-31+G(d,p) = -115.154272

Cartesian coordinates

C -0.000000 -0.000000 1.125066

C 0.000000 0.000000 -0.199706

C 0.000000 0.000000 -1.490224

H -0.000000 -0.933809 1.694592

H 0.000000 0.933809 1.694592

Frequencies

2075.0602

3109.8600

3201.6399

1044.9847

1166.7800

1474.6029

283.6144

302.8405

1044.4021

C-PR25frag2

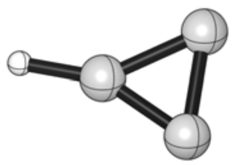

M08HX/6-31+G(d,p) = -115.300224

M08HX/6-31+G(d,p)+ZPVE = -114.629842

CCSD(T)-F12/cc-pVTZ-F12//M08HX/6-31+G(d,p) = -115.154272

Cartesian coordinates

C 0.674700 0.557069 0.000000

C -0.000000 -0.700736 -0.000000

C -0.697147 0.440177 0.000000

H 0.134684 -1.779055 -0.000000

Frequencies

1227.9072

1661.9681

3251.8738

678.6091

853.4343

890.4880

C-MIN11

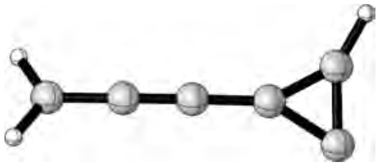

M08HX/6-31+G(d,p) = -115.300224

M08HX/6-31+G(d,p)+ZPVE = -230.067624

CCSD(T)-F12/cc-pVTZ-F12//M08HX/6-31+G(d,p) = -115.154272

Cartesian coordinates

C -2.934772 0.021192 0.002407

C -1.572140 0.006152 0.000363

C 2.263147 -0.818069 -0.076901

C 2.236787 0.577601 0.052967

C 1.044213 -0.041776 -0.003113

C -0.336001 -0.006828 -0.001672

H -3.481859 0.815920 -0.498781

H -3.495699 -0.763953 0.503423

H 2.770153 1.518396 0.151056

Frequencies

3166.9586

3251.5436

3269.6324

1460.1354

1743.4186

2030.5253

1031.7280

1263.8770

1303.1310

861.8153

939.0507

1024.1574

524.8253

608.1639

773.2751

317.2924

401.0955

486.4301

128.0060

136.0786

203.8474

C-TS171

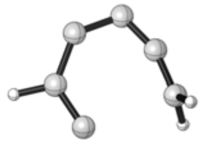

M08HX/6-31+G(d,p) = -115.300224

M08HX/6-31+G(d,p)+ZPVE = -229.949483

CCSD(T)-F12/cc-pVTZ-F12//M08HX/6-31+G(d,p) = -115.154272

Cartesian coordinates

C -0.715942 -1.473240 -0.042863

C 1.661282 -0.651878 0.055332

C 1.102699 0.548161 -0.042524

C 0.190399 1.470904 -0.139829

C -0.986486 0.951618 0.181874

C -1.483723 -0.367168 -0.035029

H 2.174192 -0.994055 0.953801

H 1.764230 -1.250743 -0.853787

H -2.547791 -0.625586 0.038221

Frequencies

3133.9434

3134.5292

3229.6786

1494.8590

1519.7756

1890.0468

1019.7460

1150.8160

1413.1266

792.6832

956.6526

994.8544

503.8976

584.1924

674.2274

346.0589

386.4016

451.3898

-371.6778

128.1677

244.1390

C-MIN35

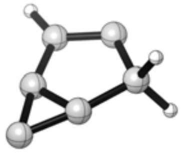

M08HX/6-31+G(d,p) = -115.300224

M08HX/6-31+G(d,p)+ZPVE = -230.03207

CCSD(T)-F12/cc-pVTZ-F12//M08HX/6-31+G(d,p) = -115.154272

Cartesian coordinates

C 0.914921 1.130488 -0.000000

C 1.345700 -0.330801 -0.000000

C 0.000000 -0.961197 0.000000

C -1.347837 -1.380465 0.000000

C -0.983772 0.009328 0.000000

C -0.418451 1.347760 0.000000

H 1.940495 -0.593478 -0.884933

H 1.940495 -0.593478 0.884933

H -0.944352 2.296283 0.000000

Frequencies

3081.9906

3135.9252

3266.3159

1391.5732

1543.8909

1675.1458

1170.7110

1240.5026

1345.6925

916.0374

1106.2498

1139.2682

828.8296

862.2937

887.0679

555.4781

731.5537

783.6312

246.9976

322.7016

461.9635

C-TS258

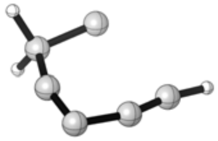

M08HX/6-31+G(d,p) = -115.300224

M08HX/6-31+G(d,p)+ZPVE = -229.918839

CCSD(T)-F12/cc-pVTZ-F12//M08HX/6-31+G(d,p) = -115.154272

Cartesian coordinates

C -0.754095 1.417418 0.254873

C -1.581603 0.187667 -0.163445

C -0.676628 -0.919431 0.059754

C 0.428583 -1.498521 0.086163

C 1.260567 -0.345963 -0.002803

C 1.679271 0.800949 -0.101802

H -2.422087 0.226625 0.552948

H -1.968343 0.217554 -1.196227

H 2.253857 1.703116 -0.153161

Frequencies

3002.0598

3062.6181

3476.1046

1417.9727

2011.9964

2125.6687

1056.3763

1081.8227

1182.0526

718.5795

892.7762

957.8428

522.7055

608.6686

659.1745

297.0321

383.2814

436.9905

-309.0344

155.0679

258.9883

C-MIN91

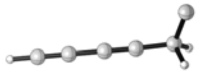

M08HX/6-31+G(d,p) = -115.300224

M08HX/6-31+G(d,p)+ZPVE = -229.998785

CCSD(T)-F12/cc-pVTZ-F12//M08HX/6-31+G(d,p) = -115.154272

Cartesian coordinates

C 2.082422 -0.386766 -0.014859

C -3.154759 0.164012 -0.002528

C -1.952317 0.030085 -0.000703

C -0.580986 -0.123720 0.001737

C 0.621972 -0.264805 0.009436

C 2.874280 0.853025 -0.022220

H 2.422286 -1.114675 -0.774842

H -4.218895 0.284497 -0.004651

H 2.452938 -0.800818 0.954314

Frequencies

2926.0314

3016.7206

3479.5886

1335.4116

2213.9367

2404.2006

1114.7251

1165.0882

1287.8257

817.2827

823.0251

986.2196

659.1877

663.6995

699.2655

329.0487

421.4040

474.7235

99.0373

145.8452

254.6089

C-TS63

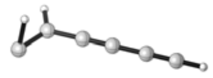

M08HX/6-31+G(d,p) = -115.300224

M08HX/6-31+G(d,p)+ZPVE = -229.992789

CCSD(T)-F12/cc-pVTZ-F12//M08HX/6-31+G(d,p) = -115.154272

Cartesian coordinates

C -2.042638 0.363224 -0.035792

C 3.171820 -0.154298 0.019215

C 1.968518 -0.028858 0.002296

C 0.599424 0.114388 -0.018481

C -0.607336 0.240523 -0.040642

C -2.931528 -0.704741 -0.091774

H -2.418991 1.404042 -0.064606

H 4.237167 -0.267294 0.034008

H -2.767742 -0.118177 1.021664

Frequencies

2378.4607

2995.8902

3475.7066

1424.8221

2210.0125

2237.9304

995.9028

1172.9489

1228.3369

743.2018

811.8217

845.3568

655.4860

667.5220

705.7290

266.1171

330.2640

497.5983

-1107.6453

106.0719

142.4650

C-MIN7

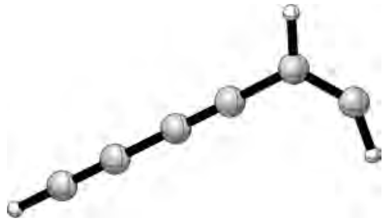

M08HX/6-31+G(d,p) = -115.300224

M08HX/6-31+G(d,p)+ZPVE = -230.079865

CCSD(T)-F12/cc-pVTZ-F12//M08HX/6-31+G(d,p) = -115.154272

Cartesian coordinates

C 0.874371 -1.892783 -0.000000

C -0.845811 3.051380 0.000000

C -0.450238 1.906987 0.000000

C 0.000000 0.605281 0.000000

C 0.390952 -0.544929 -0.000000

C 0.069748 -2.942840 -0.000000

H 1.959843 -2.033588 -0.000000

H -1.194709 4.063799 0.000000

H -0.999273 -3.128789 0.000000

Frequencies

3137.8557

3266.8007

3482.1368

1654.8325

2207.5109

2376.2495

911.9036

1196.8786

1258.0768

808.6427

832.9777

879.1278

660.6094

692.2955

729.2019

367.1031

477.8186

658.1802

112.1930

153.1012

264.5608

C-TS20

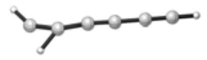

M08HX/6-31+G(d,p) = -115.300224

M08HX/6-31+G(d,p)+ZPVE = -230.016281

CCSD(T)-F12/cc-pVTZ-F12//M08HX/6-31+G(d,p) = -115.154272

Cartesian coordinates

C -0.187367 1.901189 0.000000

C 0.111661 -3.263844 -0.000000

C 0.066184 -2.051345 -0.000000

C 0.019300 -0.682958 -0.000000

C 0.000000 0.541122 0.000000

C 0.029482 3.177241 0.000000

H -1.193341 2.658909 0.000000

H 0.150438 -4.334008 -0.000000

H 0.807335 3.946666 0.000000

Frequencies

2383.6801

3123.8621

3479.8061

1916.0741

2196.1864

2278.7582

811.2553

815.7460

1170.8948

621.6774

663.1832

749.8936

450.5521

551.7952

604.8935

263.0290

268.4588

358.3987

-2229.1008

110.6710

114.6674

C-PR29frag1

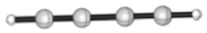

M08HX/6-31+G(d,p) = -153.442594

M08HX/6-31+G(d,p)+ZPVE = -153.40303

CCSD(T)-F12/cc-pVTZ-F12//M08HX/6-31+G(d,p) = -153.251749

Cartesian coordinates

C 0.000000 -0.000000 -1.899990

C 0.000000 -0.000000 -0.690454

C -0.000000 -0.000000 0.690454

C -0.000000 -0.000000 1.899990

H 0.000000 -0.000000 -2.971007

H -0.000000 -0.000000 2.971007

Frequencies

3479.3959

2155.1473

2337.0791

3478.9210

857.2185

857.2185

917.3129

644.8397

726.3696

726.3696

270.9663

270.9663

644.8397

C-PR29frag2

M08HX/6-31+G(d,p) = -191.459563

M08HX/6-31+G(d,p)+ZPVE = -76.569757

CCSD(T)-F12/cc-pVTZ-F12//M08HX/6-31+G(d,p) = -191.211643

Cartesian coordinates

C -0.000000 0.000000 0.732806

C 0.000000 -0.000000 -0.475059

H 0.000000 -0.000000 -1.546479

Frequencies

3476.4235

507.8749

507.8749

2130.8631

C-MIN46

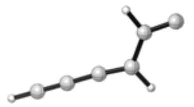

M08HX/6-31+G(d,p) = -153.442594

M08HX/6-31+G(d,p)+ZPVE = -230.023631

CCSD(T)-F12/cc-pVTZ-F12//M08HX/6-31+G(d,p) = -153.251749

Cartesian coordinates

C 1.111515 2.935974 -0.000000

C 0.417695 1.825449 -0.000000

C 0.836767 0.418211 -0.000000

C -0.000000 -0.596667 0.000000

C -0.755380 -1.685422 0.000000

C -1.472110 -2.690615 0.000000

H -0.652692 2.088086 -0.000000

H 1.915873 0.231739 0.000000

H -2.094111 -3.561409 0.000000

Frequencies

3091.6646

3119.8538

3467.1237

1756.0511

1825.8950

1987.1380

1000.6433

1100.3095

1307.3633

712.6078

815.8877

818.6389

517.5967

590.7133

643.1597

243.8750

395.2564

439.6399

54.9219

115.2240

185.9045

C-TS45

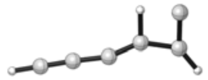

M08HX/6-31+G(d,p) = -153.442594

M08HX/6-31+G(d,p)+ZPVE = -229.99778

CCSD(T)-F12/cc-pVTZ-F12//M08HX/6-31+G(d,p) = -153.251749

Cartesian coordinates

C 1.336402 1.720316 -0.000000

C -1.271002 -2.756897 -0.000000

C -0.689905 -1.674860 -0.000000

C 0.000000 -0.526294 -0.000000

C 0.311663 0.734054 0.000000

C 0.314316 2.582053 0.000000

H 2.417536 1.738311 -0.000000

H -1.775632 -3.700527 -0.000000

H -0.650745 1.491992 0.000000

Frequencies

2241.7216

3314.7805

3476.1759

1598.5081

1896.5138

2032.2201

927.6270

1012.9951

1320.6329

720.0497

726.3196

847.4728

530.1401

570.4370

650.0916

281.0271

404.5904

494.9283

-675.8104

76.0992

142.3080

C-PR154frag1

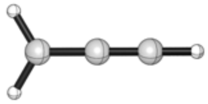

M08HX/6-31+G(d,p) = -153.442594

M08HX/6-31+G(d,p)+ZPVE = -115.928075

CCSD(T)-F12/cc-pVTZ-F12//M08HX/6-31+G(d,p) = -153.251749

Cartesian coordinates

C 0.000000 -0.000000 0.118715

C 0.000000 -0.000000 1.343613

C -0.000000 0.000000 -1.260382

H -0.000000 0.935083 -1.812602

H 0.000000 -0.000000 2.413527

H 0.000000 -0.935083 -1.812602

Frequencies

3181.8852

3285.2136

3475.1136

1077.5812

1452.7035

2033.9848

663.2954

694.8484

1030.8890

364.4941

421.9254

555.6220

C-PR154frag2

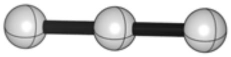

M08HX/6-31+G(d,p) = -114.011494

M08HX/6-31+G(d,p)+ZPVE = -114.002987

CCSD(T)-F12/cc-pVTZ-F12//M08HX/6-31+G(d,p) = -113.869911

Cartesian coordinates

C -0.000000 0.000000 -1.295335

C 0.000000 0.000000 0.000000

C 0.000000 0.000000 1.295335

Frequencies

2171.1212

149.2533

149.2533

1264.6210

C-MIN50

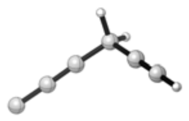

M08HX/6-31+G(d,p) = -114.011494

M08HX/6-31+G(d,p)+ZPVE = -230.018885

CCSD(T)-F12/cc-pVTZ-F12//M08HX/6-31+G(d,p) = -113.869911

Cartesian coordinates

C 0.528270 2.658891 0.000000

C 0.806092 1.485958 -0.000000

C 1.147752 0.055037 -0.000000

C -0.000000 -0.841475 -0.000000

C -2.099463 -2.354595 0.000000

C -1.019049 -1.558516 0.000000

H 0.274212 3.699445 0.000000

H 1.772091 -0.185624 0.876798

H 1.772091 -0.185624 -0.876798

Frequencies

3024.0866

3065.9841

3480.9861

1403.7279

2008.9875

2275.6766

1198.4490

1295.0817

1368.8333

801.7458

856.3706

968.2377

513.1719

692.3285

700.8847

325.3133

328.0398

355.3724

37.3309

164.8151

231.2997

C-TS208

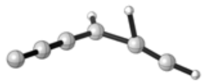

M08HX/6-31+G(d,p) = -114.011494

M08HX/6-31+G(d,p)+ZPVE = -229.938731

CCSD(T)-F12/cc-pVTZ-F12//M08HX/6-31+G(d,p) = -113.869911

Cartesian coordinates

C 1.663489 0.024856 0.066810

C 2.564245 -0.802249 -0.082481

C -3.040754 -0.695654 0.045967

C -1.874005 -0.120639 -0.015954

C -0.739921 0.470863 -0.096010

C 0.518128 0.950515 -0.061698

H 1.304897 0.542789 1.128253

H 3.368287 -1.510160 -0.091266

H 0.779718 2.001221 -0.176785

Frequencies

2105.5674

3196.8299

3463.8700

1625.7217

1908.6901

1980.6499

921.2227

991.9792

1250.4787

648.7379

733.4012

857.4534

424.4554

459.0539

564.9574

222.0874

360.8872

377.6629

-1961.5198

76.2573

178.4573

C-MIN58

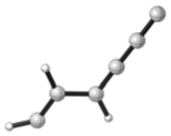

M08HX/6-31+G(d,p) = -114.011494

M08HX/6-31+G(d,p)+ZPVE = -230.010914

CCSD(T)-F12/cc-pVTZ-F12//M08HX/6-31+G(d,p) = -113.869911

Cartesian coordinates

C -1.467187 -0.774579 0.000000

C -0.000000 -0.791447 0.000000

C 0.747387 0.288987 -0.000000

C 1.489798 1.347189 -0.000000

C 2.215736 2.412563 -0.000000

C -2.203289 -1.870549 0.000000

H -1.952939 0.209444 0.000000

H 0.514358 -1.755778 0.000000

H -3.256083 -2.126657 0.000000

Frequencies

3102.5525

3168.0260

3290.4385

1665.3741

1771.3227

2168.7630

1134.9848

1217.5531

1302.7754

788.3508

861.0067

966.3270

590.7245

632.2969

775.1468

236.7717

401.4458

521.8833

93.4139

116.3237

228.4132

C-TS67

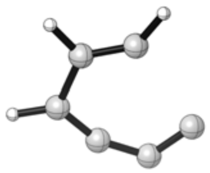

M08HX/6-31+G(d,p) = -114.011494

M08HX/6-31+G(d,p)+ZPVE = -229.991028

CCSD(T)-F12/cc-pVTZ-F12//M08HX/6-31+G(d,p) = -113.869911

Cartesian coordinates

C -1.212270 0.820147 0.000000

C -1.229031 -0.665288 0.000000

C -0.000000 -1.132684 0.000000

C 1.292098 -1.069096 0.000000

C 1.950071 0.051513 -0.000000

C -0.107708 1.551682 -0.000000

H -2.193603 1.309243 0.000000

H -2.148649 -1.239816 0.000000

H 0.183289 2.592921 -0.000000

Frequencies

3116.7275

3267.9612

3324.8839

1603.6686

1735.8555

2023.8320

1087.1073

1211.7013

1322.1841

808.8957

842.0298

983.4324

630.7173

631.9464

733.8361

267.5903

449.6490

463.2138

-254.8533

218.4524

241.8718

C-MIN3

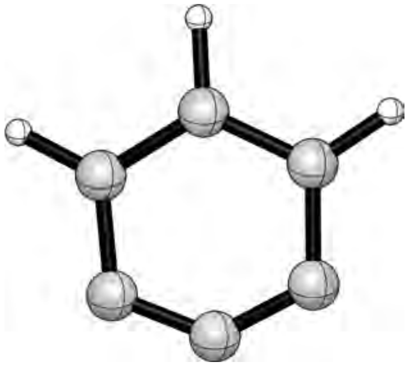

M08HX/6-31+G(d,p) = -114.011494

M08HX/6-31+G(d,p)+ZPVE = -230.105128

CCSD(T)-F12/cc-pVTZ-F12//M08HX/6-31+G(d,p) = -113.869911

Cartesian coordinates

C -0.000000 0.000000 -1.223001

C 0.000000 1.239622 -0.549542

C 0.000000 1.178932 0.846485

C 0.000000 -0.000000 1.383309

C -0.000000 -1.178932 0.846485

C -0.000000 -1.239622 -0.549542

H -0.000000 0.000000 -2.313997

H 0.000000 2.175948 -1.105584

H -0.000000 -2.175948 -1.105584

Frequencies

3189.5103

3209.4401

3214.8511

1426.2364

1486.8342

1592.4321

1175.4049

1306.5830

1369.4457

1037.9607

1119.3715

1131.5130

832.6290

888.3214

968.4820

557.1202

570.3810

765.7454

411.7945

411.9031

439.1495

C-TS58

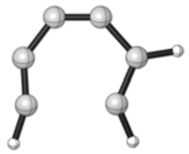

M08HX/6-31+G(d,p) = -114.011494

M08HX/6-31+G(d,p)+ZPVE = -229.994013

CCSD(T)-F12/cc-pVTZ-F12//M08HX/6-31+G(d,p) = -113.869911

Cartesian coordinates

C 1.366789 -0.534083 -0.000000

C -0.803797 -1.579926 0.000000

C -1.401722 -0.490398 0.000000

C -1.116718 0.848070 -0.000000

C -0.000000 1.369888 -0.000000

C 1.331297 0.801062 -0.000000

H 2.177574 -1.256372 0.000000

H -0.670165 -2.645884 0.000000

H 2.237499 1.414582 -0.000000

Frequencies

3129.2663

3261.1341

3426.7512

1529.3687

1930.0550

2041.6596

1018.4764

1217.8775

1229.7845

690.1724

780.5697

833.4553

505.1899

635.9347

666.2811

305.8865

412.9647

430.7534

-609.2217

200.3351

305.6748

C-PR32frag1

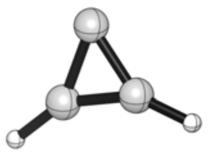

M08HX/6-31+G(d,p) = -115.33042

M08HX/6-31+G(d,p)+ZPVE = -115.297955

CCSD(T)-F12/cc-pVTZ-F12//M08HX/6-31+G(d,p) = -115.178046

Cartesian coordinates

C 0.000000 0.000000 -0.934743

C -0.000000 -0.664094 0.322234

C -0.000000 0.664094 0.322234

H -0.000000 -1.600330 0.870826

H -0.000000 1.600330 0.870826

Frequencies

1671.3914

3248.8989

3285.4422

992.0820

1114.6606

1341.7632

787.9506

898.6547

909.5079

C-PR32frag2

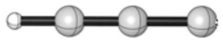

M08HX/6-31+G(d,p) = -115.33042

M08HX/6-31+G(d,p)+ZPVE = -114.62137

CCSD(T)-F12/cc-pVTZ-F12//M08HX/6-31+G(d,p) = -115.178046

Cartesian coordinates

C 1.929356 -0.256006 0.022264

C 2.871665 0.328672 -0.744695

C 1.061707 -0.794363 0.729023

H 0.312214 -1.259260 1.338840

Frequencies

1160.0133

1900.3043

3456.2901

241.2476

375.7438

658.5500

C-MIN24

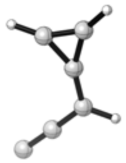

M08HX/6-31+G(d,p) = -115.33042

M08HX/6-31+G(d,p)+ZPVE = -230.044553

CCSD(T)-F12/cc-pVTZ-F12//M08HX/6-31+G(d,p) = -115.178046

Cartesian coordinates

C -1.373003 1.075311 0.000000

C -0.000000 0.740657 0.000000

C 0.992695 -0.213092 -0.000000

C 0.657287 -1.553413 -0.000000

C 0.317722 -2.785789 -0.000000

C -0.484246 2.069735 0.000000

H -2.405298 0.746408 0.000000

H 2.032699 0.117027 -0.000000

H -0.290129 3.136113 0.000000

Frequencies

3176.0889

3272.6065

3315.4534

1539.6088

1803.9513

2057.0950

1099.1922

1167.2033

1364.4774

933.8046

969.0776

1008.4390

742.4123

819.2533

851.8219

362.5794

449.8234

554.0106

118.7737

157.6768

320.0887

C-TS210

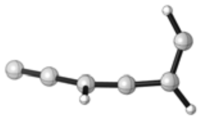

M08HX/6-31+G(d,p) = -115.33042

M08HX/6-31+G(d,p)+ZPVE = -229.938445

CCSD(T)-F12/cc-pVTZ-F12//M08HX/6-31+G(d,p) = -115.178046

Cartesian coordinates

C 1.983849 -0.411797 -0.147565

C 0.588820 -0.409206 -0.081474

C -0.668097 -0.348991 0.210076

C -1.953614 0.049754 -0.014753

C -3.157119 0.399508 -0.166278

C 2.506387 0.810266 0.039489

H 2.562812 -1.273442 -0.486247

H -0.456838 -1.034394 1.104840

H 2.092669 1.770635 0.344442

Frequencies

2709.5201

3169.0397

3231.0392

1522.8434

1922.6035

2099.7885

924.5260

1207.5977

1283.4746

731.5555

881.6499

919.0433

411.3346

617.4244

667.7725

185.8039

290.5603

357.3003

-328.6274

83.9318

180.8419

C-PR20frag1

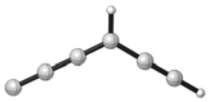

M08HX/6-31+G(d,p) = -229.495905

M08HX/6-31+G(d,p)+ZPVE = -229.44892

CCSD(T)-F12/cc-pVTZ-F12//M08HX/6-31+G(d,p) = -229.205636

Cartesian coordinates

C 0.992764 -0.099026 -0.000000

C -0.953421 1.634814 0.000000

C -1.914721 2.495822 0.000000

C 0.654572 -2.710116 -0.000000

C 0.798893 -1.510187 -0.000000

C 0.000000 0.766859 0.000000

H 0.521814 -3.772994 -0.000000

H 2.009668 0.304002 -0.000000

Frequencies

2255.8076

3152.7735

3479.2962

1287.8280

1766.3346

2187.3121

756.4528

900.4903

1063.0017

607.2475

666.7993

711.0582

376.7703

393.2571

510.4545

93.9025

201.6339

213.4393

C-PR20frag2

M08HX/6-31+G(d,p) = -229.495905

M08HX/6-31+G(d,p)+ZPVE = -0.499294

CCSD(T)-F12/cc-pVTZ-F12//M08HX/6-31+G(d,p) = -229.205636

Cartesian coordinates

H 0.000000 0.000000 0.000000

C-MIN5

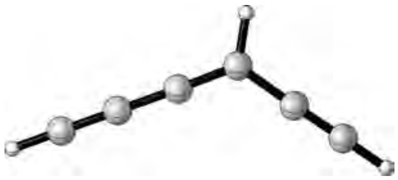

M08HX/6-31+G(d,p) = -229.495905

M08HX/6-31+G(d,p)+ZPVE = -230.091844

CCSD(T)-F12/cc-pVTZ-F12//M08HX/6-31+G(d,p) = -229.205636

Cartesian coordinates

C 1.822994 0.010214 0.000005

C 2.823172 -0.670350 0.000009

C -2.990641 -0.468789 -0.000000

C -1.825532 -0.059295 -0.000000

C -0.576045 0.376241 -0.000001

C 0.657486 0.842383 -0.000002

H 3.702627 -1.281146 -0.000056

H -3.998864 -0.827423 0.000000

H 0.827634 1.926143 -0.000011

Frequencies

3097.8416

3471.1962

3480.1808

1821.7464

1983.2739

2241.4544

859.5957

1054.7626

1308.2201

699.1633

717.0138

811.5315

530.8813

593.4420

674.3692

368.5909

411.1462

496.7345

43.5698

227.7008

243.2718

C-TS11

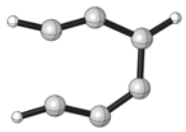

M08HX/6-31+G(d,p) = -229.495905

M08HX/6-31+G(d,p)+ZPVE = -230.030967

CCSD(T)-F12/cc-pVTZ-F12//M08HX/6-31+G(d,p) = -229.205636

Cartesian coordinates

C -1.205530 0.959047 -0.000000

C -1.097772 -1.119667 -0.000000

C 0.138191 -1.406605 -0.000000

C 1.231649 -0.635052 0.000000

C 1.294626 0.722636 0.000000

C -0.000000 1.303091 0.000000

H -2.255671 1.194409 -0.000000

H -2.131725 -1.430233 -0.000000

H 2.220414 1.295128 0.000000

Frequencies

3211.2785

3358.7851

3400.5248

1560.1354

1695.1715

1956.9665

1055.9842

1095.5908

1252.3977

737.9983

765.2268

891.5882

565.9716

625.2586

674.8716

424.7332

482.7759

542.9277

-656.1915

292.0789

401.5787

C-MIN4

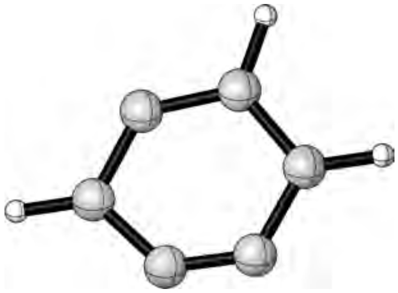

M08HX/6-31+G(d,p) = -229.495905

M08HX/6-31+G(d,p)+ZPVE = -230.101923

CCSD(T)-F12/cc-pVTZ-F12//M08HX/6-31+G(d,p) = -229.205636

Cartesian coordinates

C -1.194475 0.542609 -0.000000

C -0.000000 1.256611 -0.000000

C 1.310421 0.800687 -0.000000

C 1.190964 -0.583705 0.000000

C 0.171377 -1.311137 0.000000

C -1.146923 -0.870385 0.000000

H -2.150883 1.064866 -0.000000

H 2.206609 1.414765 -0.000000

H -2.043913 -1.487716 0.000000

Frequencies

3202.3768

3217.0082

3237.5116

1418.6789

1498.8906

2011.7008

1237.1725

1310.6530

1402.6742

1000.5273

1082.8881

1105.7881

800.3412

855.3310

948.2839

577.2371

593.9268

774.8711

376.7988

450.9207

472.1114

C-TS43

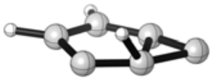

M08HX/6-31+G(d,p) = -229.495905

M08HX/6-31+G(d,p)+ZPVE = -229.998738

CCSD(T)-F12/cc-pVTZ-F12//M08HX/6-31+G(d,p) = -229.205636

Cartesian coordinates

C 1.447973 0.162233 0.026627

C 0.480269 1.228221 -0.125257

C -0.801287 0.768140 -0.077145

C -0.605524 -0.775473 0.022457

C -1.845433 -0.300563 -0.005506

C 0.782994 -1.047570 0.012148

H 2.525993 0.300351 0.079101

H -0.493903 1.533446 0.848931

H 1.213954 -2.043728 -0.047980

Frequencies

2255.6317

3226.4208

3248.0981

1450.1167

1516.0554

1730.6763

1093.8166

1113.0687

1349.1182

880.2738

947.8516

1007.9375

721.2191

807.4291

817.0651

517.3575

580.0985

624.3019

-1421.6162

310.8015

352.1960

C-MIN1

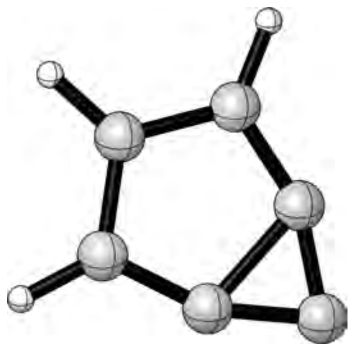

M08HX/6-31+G(d,p) = -229.495905

M08HX/6-31+G(d,p)+ZPVE = -230.1192

CCSD(T)-F12/cc-pVTZ-F12//M08HX/6-31+G(d,p) = -229.205636

Cartesian coordinates

C 1.014079 0.920816 -0.000000

C 1.229188 -0.471400 0.000000

C -0.000000 -1.097676 0.000000

C -1.092507 0.105623 -0.000000

C -1.342483 -1.218967 0.000000

C -0.350923 1.268900 -0.000000

H 1.822348 1.654460 -0.000000

H 2.191175 -0.972859 0.000000

H -0.757651 2.274626 -0.000000

Frequencies

3190.1665

3256.7688

3261.9253

1433.4021

1562.0442

1838.8612

1107.2401

1288.9122

1351.5427

952.6053

1065.6611

1085.7818

796.5034

819.3938

930.7951

592.9497

609.9558

771.0043

358.8366

585.3920

591.0134

C-TS6

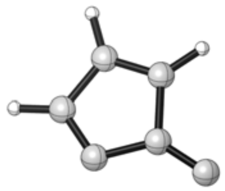

M08HX/6-31+G(d,p) = -229.495905

M08HX/6-31+G(d,p)+ZPVE = -230.048799

CCSD(T)-F12/cc-pVTZ-F12//M08HX/6-31+G(d,p) = -229.205636

Cartesian coordinates

C 0.544888 1.303677 -0.000000

C -0.926653 1.196839 -0.000000

C -1.218456 -0.113222 0.000000

C 0.213494 -2.220139 0.000000

C -0.000000 -0.923414 0.000000

C 1.114435 0.074615 -0.000000

H 1.090878 2.244786 -0.000000

H -1.624610 2.028606 -0.000000

H 2.167482 -0.183533 -0.000000

Frequencies

3227.6629

3254.0941

3277.4415

1540.7089

1627.1635

1769.3186

1110.5269

1230.5106

1333.3230

926.1067

1008.6735

1039.4596

802.7545

825.6128

922.3467

578.9683

658.3382

723.6684

-86.3406

150.1346

465.9810

C-TS74

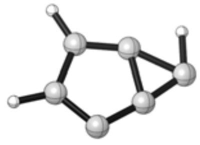

M08HX/6-31+G(d,p) = -229.495905

M08HX/6-31+G(d,p)+ZPVE = -229.987513

CCSD(T)-F12/cc-pVTZ-F12//M08HX/6-31+G(d,p) = -229.205636

Cartesian coordinates

C -1.251982 0.374433 -0.000000

C -0.000000 0.955407 -0.000000

C 1.560513 1.031188 -0.000000

C 1.000292 -0.166957 0.000000

C 0.282297 -1.336393 0.000000

C -1.075033 -1.050529 0.000000

H -2.196567 0.909928 -0.000000

H 0.978168 2.034643 -0.000000

H -1.878113 -1.787466 0.000000

Frequencies

2799.6602

3209.1574

3249.1379

1358.2780

1516.0806

1834.2643

1090.5257

1225.2651

1334.6311

849.2092

937.4479

1041.6062

637.1869

792.7176

820.8595

507.8710

540.8768

588.4328

-1622.2126

254.8591

486.3944

C-MIN3

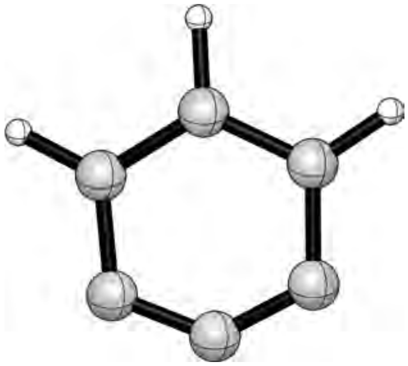

M08HX/6-31+G(d,p) = -229.495905

M08HX/6-31+G(d,p)+ZPVE = -230.105128

CCSD(T)-F12/cc-pVTZ-F12//M08HX/6-31+G(d,p) = -229.205636

Cartesian coordinates

C -0.000000 0.000000 -1.223001

C 0.000000 1.239622 -0.549542

C 0.000000 1.178932 0.846485

C 0.000000 -0.000000 1.383309

C -0.000000 -1.178932 0.846485

C -0.000000 -1.239622 -0.549542

H -0.000000 0.000000 -2.313997

H 0.000000 2.175948 -1.105584

H -0.000000 -2.175948 -1.105584

Frequencies

3189.5103

3209.4401

3214.8511

1426.2364

1486.8342

1592.4321

1175.4049

1306.5830

1369.4457

1037.9607

1119.3715

1131.5130

832.6290

888.3214

968.4820

557.1202

570.3810

765.7454

411.7945

411.9031

439.1495

C-PR44frag1

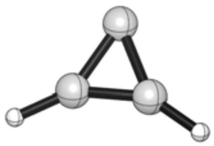

M08HX/6-31+G(d,p) = -115.33042

M08HX/6-31+G(d,p)+ZPVE = -115.297955

CCSD(T)-F12/cc-pVTZ-F12//M08HX/6-31+G(d,p) = -115.178046

Cartesian coordinates

C 0.000000 0.000000 -0.934743

C -0.000000 -0.664094 0.322234

C -0.000000 0.664094 0.322234

H -0.000000 -1.600330 0.870826

H -0.000000 1.600330 0.870826

Frequencies

1671.3914

3248.8989

3285.4422

992.0820

1114.6606

1341.7632

787.9506

898.6547

909.5079

C-PR44frag2

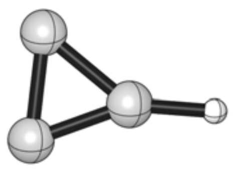

M08HX/6-31+G(d,p) = -115.33042

M08HX/6-31+G(d,p)+ZPVE = -114.629842

CCSD(T)-F12/cc-pVTZ-F12//M08HX/6-31+G(d,p) = -115.178046

Cartesian coordinates

C 0.697146 0.440172 -0.000000

C -0.674705 0.557072 -0.000000

H -0.134645 -1.779054 0.000000

C -0.000000 -0.700735 0.000000

Frequencies

1227.8995

1661.9770

3251.9226

678.6438

853.4130

890.4688

C-MIN33

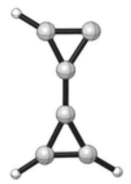

M08HX/6-31+G(d,p) = -115.33042

M08HX/6-31+G(d,p)+ZPVE = -230.036241

CCSD(T)-F12/cc-pVTZ-F12//M08HX/6-31+G(d,p) = -115.178046

Cartesian coordinates

C -0.642300 -1.886374 0.000000

C 0.688299 -1.859161 -0.000000

C 0.000000 -0.607783 -0.000000

C 0.007506 0.768599 0.000000

C -0.603953 1.983399 0.000000

H -1.548829 -2.480619 0.000000

H 1.619759 -2.413264 -0.000000

H -1.527431 2.553529 0.000000

C 0.793199 1.991379 -0.000000

Frequencies

3256.2744

3262.3924

3308.1006

1477.3534

1651.0781

1888.7376

1071.2281

1093.4364

1313.3350

904.7007

922.2055

968.7718

658.9690

787.0283

890.3690

390.6891

494.4571

605.3591

141.7305

173.5071

187.6288

C-TS146

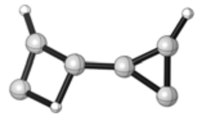

M08HX/6-31+G(d,p) = -115.33042

M08HX/6-31+G(d,p)+ZPVE = -229.963407

CCSD(T)-F12/cc-pVTZ-F12//M08HX/6-31+G(d,p) = -115.178046

Cartesian coordinates

C 1.958628 -0.835029 -0.180324

C 2.000994 0.545780 0.036301

C 0.776310 -0.026817 0.028296

C -0.599551 0.024527 0.219345

C -2.227007 -0.655456 -0.032303

C -1.791626 0.635064 -0.095163

H 2.589308 1.448423 0.166637

H -1.101258 -1.170474 0.368407

H -2.194532 1.593632 -0.391959

Frequencies

2265.5677

3261.7687

3317.6533

1423.1204

1588.6288

1774.3170

1027.6020

1121.9949

1311.7973

848.1543

967.1722

1018.6570

611.7060

737.1939

835.5635

215.9113

417.3158

557.6889

-585.8382

87.7787

162.3998

C-MIN96

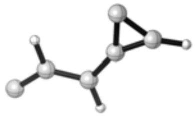

M08HX/6-31+G(d,p) = -115.33042

M08HX/6-31+G(d,p)+ZPVE = -229.998043

CCSD(T)-F12/cc-pVTZ-F12//M08HX/6-31+G(d,p) = -115.178046

Cartesian coordinates

C 0.000000 -0.824094 -0.000000

C -1.410155 -0.682371 -0.000000

C -2.393267 -1.578163 -0.000000

C 0.768818 1.783813 0.000000

C 0.799344 0.352258 0.000000

C 1.959399 1.024828 0.000000

H -1.842251 0.336442 0.000000

H 3.044995 1.018844 0.000000

H 0.452426 -1.812911 -0.000000

Frequencies

3049.1405

3214.7064

3257.8257

1385.4155

1563.6975

1769.6180

1147.2874

1226.6962

1306.1854

866.2672

952.2510

990.6026

748.8332

760.7939

799.4818

235.7990

428.5439

500.9944

103.2840

125.6017

185.8162

C-TS79

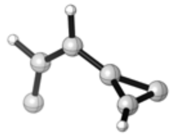

M08HX/6-31+G(d,p) = -115.33042

M08HX/6-31+G(d,p)+ZPVE = -229.98592

CCSD(T)-F12/cc-pVTZ-F12//M08HX/6-31+G(d,p) = -115.178046

Cartesian coordinates

C -0.513344 0.897203 0.033053

C -1.720459 0.173687 0.023849

C -2.064709 -1.116180 -0.017888

C 1.837245 -0.271862 -0.842152

C 0.769555 0.247145 -0.033934

C 1.836729 -0.288244 0.568044

H -2.684511 0.714355 0.060894

H 2.338782 -0.547867 1.496401

H -0.524371 1.983017 0.056873

Frequencies

3054.7398

3235.0820

3242.0254

1375.0777

1532.1461

1770.9372

1085.7973

1145.7203

1290.6207

900.6453

934.7807

1017.7410

571.0357

718.3373

770.9768

286.4655

378.4546

528.1740

-166.2887

116.7781

228.2941

C-TS73

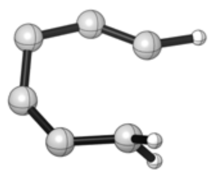

M08HX/6-31+G(d,p) = -115.33042

M08HX/6-31+G(d,p)+ZPVE = -229.987987

CCSD(T)-F12/cc-pVTZ-F12//M08HX/6-31+G(d,p) = -115.178046

Cartesian coordinates

C 1.470582 0.396207 0.045402

C 1.059931 -0.930550 -0.004028

C -0.135791 -1.342310 -0.116007

C -1.323889 -0.799375 0.134838

C -1.269684 0.529634 -0.045237

C -0.393103 1.456209 0.004343

H 2.069592 0.766621 0.883552

H 1.723680 0.853131 -0.917356

H -0.241547 2.521360 -0.082066

Frequencies

3099.9921

3182.3965

3352.2456

1481.9655

1677.7268

1894.2420

1112.7857

1231.1057

1418.8226

819.6557

986.1033

1005.6914

551.7955

593.9296

715.3856

374.1637

456.6648

482.1678

-394.4666

219.1070

287.4810

C-MIN71

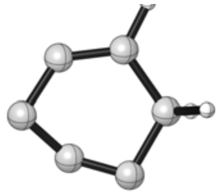

M08HX/6-31+G(d,p) = -115.33042

M08HX/6-31+G(d,p)+ZPVE = -230.004912

CCSD(T)-F12/cc-pVTZ-F12//M08HX/6-31+G(d,p) = -115.178046

Cartesian coordinates

C 1.327378 -0.058194 0.033016

C 0.573487 -1.364810 0.046194

C -0.663381 -1.019139 -0.113669

C -0.878808 1.036330 -0.038908

C -1.634712 -0.141957 0.099012

C 0.454162 1.198230 0.011685

H 2.144447 -0.022468 0.770737

H 1.841506 -0.050683 -0.951446

H 0.945292 2.170388 -0.043272

Frequencies

2987.5468

3072.5801

3205.4706

1341.4545

1540.4164

1647.5803

1184.4807

1267.8299

1282.8441

973.6243

1011.5531

1075.8834

731.0909

833.2227

893.7841

458.7382

587.3485

625.9987

172.7094

276.4118

385.7268

C-TS5

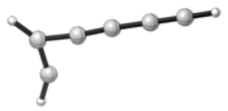

M08HX/6-31+G(d,p) = -115.33042

M08HX/6-31+G(d,p)+ZPVE = -230.048834

CCSD(T)-F12/cc-pVTZ-F12//M08HX/6-31+G(d,p) = -115.178046

Cartesian coordinates

C -1.258765 -1.818480 0.000000

C 1.226965 2.796010 -0.000000

C 0.646085 1.725119 -0.000000

C 0.000000 0.529349 -0.000000

C -0.530756 -0.595407 0.000000

C -0.074010 -2.370066 0.000000

H -2.320391 -2.069014 0.000000

H 1.730856 3.740189 -0.000000

H 0.532420 -3.270322 0.000000

Frequencies

3174.6753

3266.0187

3482.2906

1693.1951

2048.5212

2160.6082

859.3676

1106.3463

1292.5697

708.1860

728.7735

755.9031

579.3371

621.9497

671.1938

336.0585

365.3210

562.3722

-730.0818

122.9592

159.7003

F-PR72frag1

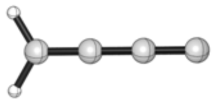

M08HX/6-31+G(d,p) = -153.3749

M08HX/6-31+G(d,p)+ZPVE = -153.338821

CCSD(T)-F12/cc-pVTZ-F12//M08HX/6-31+G(d,p) = -153.17851

Cartesian coordinates

C 0.000000 0.000000 0.844641

C 0.000000 0.000000 2.130610

C -0.000000 -0.000000 -0.451620

C -0.000000 -0.000000 -1.755072

H 0.000000 0.940243 -2.305679

H -0.000000 -0.940243 -2.305679

Frequencies

2191.4777

3162.2444

3253.9772

955.3300

1366.5311

1784.0530

550.3946

811.3152

941.0262

184.4333

205.3956

430.5742

F-PR72frag2

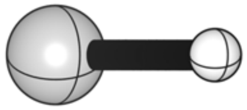

M08HX/6-31+G(d,p) = -153.3749

M08HX/6-31+G(d,p)+ZPVE = -38.465931

CCSD(T)-F12/cc-pVTZ-F12//M08HX/6-31+G(d,p) = -153.17851

Cartesian coordinates

C -0.000000 0.000000 0.160714

H 0.000000 -0.000000 -0.964287

Frequencies

2874.9404

F-MIN28

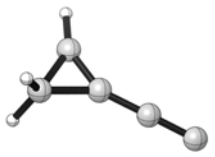

M08HX/6-31+G(d,p) = -153.3749

M08HX/6-31+G(d,p)+ZPVE = -191.960853

CCSD(T)-F12/cc-pVTZ-F12//M08HX/6-31+G(d,p) = -153.17851

Cartesian coordinates

C 0.520380 1.105757 0.000000

C 0.999061 2.294734 0.000000

C 0.179124 -1.477695 0.000000

C 0.000000 -0.148513 0.000000

C -1.233762 -1.018828 -0.000000

H 0.859295 -2.320680 0.000000

H -1.824060 -1.106023 -0.919239

H -1.824060 -1.106023 0.919239

Frequencies

3065.7563

3145.0498

3273.6154

1453.2037

1587.2967

2057.6431

1043.1670

1092.4945

1157.2584

913.9180

935.5571

992.2173

492.6216

508.2322

676.9118

174.5522

181.5766

344.8408

F-TS21

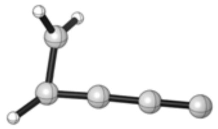

M08HX/6-31+G(d,p) = -153.3749

M08HX/6-31+G(d,p)+ZPVE = -191.954595

CCSD(T)-F12/cc-pVTZ-F12//M08HX/6-31+G(d,p) = -153.17851

Cartesian coordinates

C 1.250590 -0.075742 0.005730

C 2.500979 0.211090 0.047369

C -1.312019 -0.681114 -0.073038

C -0.032869 -0.354526 -0.047469

C -1.541673 0.729995 0.010534

H -1.873582 -1.548777 0.264307

H -1.294762 1.398519 -0.811557

H -2.021707 1.172038 0.888492

Frequencies

3116.8670

3228.4627

3245.0630

1477.1401

1734.1205

2106.2602

994.8704

1160.1531

1264.1426

743.1502

878.1413

908.1432

381.4690

496.4164

652.7829

-356.1264

157.1444

199.3877

F-MIN8

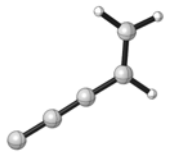

M08HX/6-31+G(d,p) = -153.3749

M08HX/6-31+G(d,p)+ZPVE = -191.987484

CCSD(T)-F12/cc-pVTZ-F12//M08HX/6-31+G(d,p) = -153.17851

Cartesian coordinates

C -1.067076 -0.977218 0.000000

C -2.187063 -1.667192 0.000000

C 1.282238 1.777900 -0.000000

C 0.000000 -0.295790 0.000000

C 1.181049 0.418089 -0.000000

H 2.260183 2.250374 -0.000000

H 2.083942 -0.199880 -0.000000

H 0.400980 2.414767 -0.000000

Frequencies

3154.7033

3179.6394

3290.1181

1503.4797

1552.1868

1889.1192

1045.4803

1248.9441

1353.1084

791.4560

879.4891

918.5495

384.3501

581.0175

603.0364

123.7503

180.9982

277.7964

F-TS113

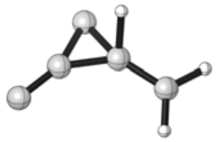

M08HX/6-31+G(d,p) = -153.3749

M08HX/6-31+G(d,p)+ZPVE = -191.858151

CCSD(T)-F12/cc-pVTZ-F12//M08HX/6-31+G(d,p) = -153.17851

Cartesian coordinates

C -1.909240 -0.833154 -0.010744

C -0.915045 -0.019274 0.077829

C 0.547678 0.188633 0.085720

C -0.283714 1.321481 -0.210036

C 1.729290 -0.558683 -0.056792

H 2.692746 -0.066774 0.034024

H 1.674289 -1.627559 -0.227351

H 0.619158 1.100317 0.877473

Frequencies

2296.7253

3193.5498

3317.3067

1350.2428

1450.8518

1888.3642

956.6716

1041.6213

1078.3134

575.3495

712.4307

790.0571

322.3623

393.5383

510.4377

-639.2456

123.1851

264.3730

F-MIN24

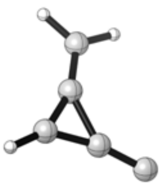

M08HX/6-31+G(d,p) = -153.3749

M08HX/6-31+G(d,p)+ZPVE = -191.963804

CCSD(T)-F12/cc-pVTZ-F12//M08HX/6-31+G(d,p) = -153.17851

Cartesian coordinates

C 0.475408 -2.005198 0.000000

C -0.227732 -0.902092 0.000000

C 0.000000 0.610160 -0.000000

C -1.249574 0.062331 -0.000000

C 0.947206 1.558302 -0.000000

H 2.001979 1.302801 -0.000000

H 0.652514 2.605435 -0.000000

H -2.326344 0.150748 -0.000000

Frequencies

3183.5459

3292.2553

3312.3955

1429.2824

1534.7510

1828.3405

1046.0903

1059.0511

1112.2529

668.3770

823.5057

848.8804

392.3089

447.9921

631.3561

85.8881

290.7199

369.9708

F-TS34

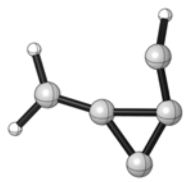

M08HX/6-31+G(d,p) = -153.3749

M08HX/6-31+G(d,p)+ZPVE = -191.936302

CCSD(T)-F12/cc-pVTZ-F12//M08HX/6-31+G(d,p) = -153.17851

Cartesian coordinates

C 0.486805 -1.380148 -0.026260

C 1.173807 -0.187286 0.011129

C -0.377695 -0.178466 -0.004678

C 0.928423 1.081062 0.078863

C -1.652537 0.209937 0.003809

H -1.957828 1.250384 0.047349

H -2.410713 -0.569393 -0.032130

H 1.015727 2.048414 -0.392399

Frequencies

3181.8814

3290.0175

3348.2900

1434.0849

1616.5469

1746.7306

868.0287

1023.7657

1197.1268

670.1327

798.4765

850.1352

378.3833

454.3583

654.5617

-488.8183

289.9356

318.2431

F-MIN1

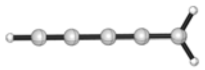

M08HX/6-31+G(d,p) = -153.3749

M08HX/6-31+G(d,p)+ZPVE = -192.054468

CCSD(T)-F12/cc-pVTZ-F12//M08HX/6-31+G(d,p) = -153.17851

Cartesian coordinates

C -0.000000 -0.000000 -1.423473

C -0.000000 -0.000000 -2.639960

C 0.000000 0.000000 2.536976

C -0.000000 -0.000000 -0.061539

C 0.000000 0.000000 1.175923

H -0.000000 -0.935099 3.091526

H -0.000000 0.935099 3.091526

H -0.000000 -0.000000 -3.710614

Frequencies

3168.9969

3270.3806

3476.2417

1462.5890

2025.4611

2168.3816

782.9503

1022.1607

1299.1722

732.3064

752.2117

780.1273

414.2457

624.7181

640.3722

159.0963

159.3869

357.5334

F-PR10frag1

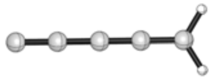

M08HX/6-31+G(d,p) = -191.431709

M08HX/6-31+G(d,p)+ZPVE = -191.390042

CCSD(T)-F12/cc-pVTZ-F12//M08HX/6-31+G(d,p) = -191.188387

Cartesian coordinates

C -0.000000 0.000000 -2.781066

C -0.000000 0.000000 -1.494318

C 0.000000 -0.000000 1.079769

C -0.000000 0.000000 -0.191046

C 0.000000 -0.000000 2.398743

H 0.000000 0.935104 2.963752

H 0.000000 -0.935104 2.963752

Frequencies

2241.2790

3121.4498

3213.3345

1373.3882

1524.6901

2011.4088

781.5701

978.4437

1026.2720

302.3957

504.5918

645.3498

140.3766

144.0062

281.4489

F-PR10frag2

M08HX/6-31+G(d,p) = -191.431709

M08HX/6-31+G(d,p)+ZPVE = -0.499294

CCSD(T)-F12/cc-pVTZ-F12//M08HX/6-31+G(d,p) = -191.188387

Cartesian coordinates

H 0.000000 0.000000 0.000000

F-MIN48

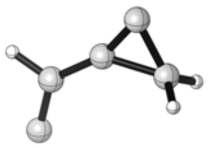

M08HX/6-31+G(d,p) = -191.431709

M08HX/6-31+G(d,p)+ZPVE = -191.914813

CCSD(T)-F12/cc-pVTZ-F12//M08HX/6-31+G(d,p) = -191.188387

Cartesian coordinates

C 0.000000 0.412565 -0.000000

C 1.569703 0.162790 0.000000

C -1.219925 -0.337206 -0.000000

C 0.792225 1.426970 -0.000000

C -1.453768 -1.628376 0.000000

H -2.189708 0.190338 -0.000000

H 2.030152 -0.205396 -0.922915

H 2.030152 -0.205396 0.922915

Frequencies

3069.5264

3070.3412

3158.3266

1507.8941

1736.8559

1943.8478

1039.1087

1082.1436

1106.9778

779.4838

924.2879

1019.9948

358.1860

643.9537

664.7040

87.9318

116.7325

273.8312

F-TS77

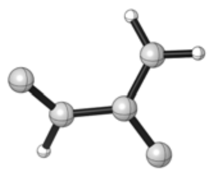

M08HX/6-31+G(d,p) = -191.431709

M08HX/6-31+G(d,p)+ZPVE = -191.892455

CCSD(T)-F12/cc-pVTZ-F12//M08HX/6-31+G(d,p) = -191.188387

Cartesian coordinates

C 0.446125 -0.174312 0.016883

C 1.048507 1.101070 -0.033675

C -1.007856 -0.402927 -0.130034

C 1.303504 -1.224650 0.086059

C -1.992195 0.397878 0.190170

H -1.395620 -1.348461 -0.538294

H 2.097592 1.222832 0.220687

H 0.509521 1.943274 -0.458815

Frequencies

3106.9554

3175.6331

3293.0787

1417.6517

1490.9792

1771.2710

963.8930

982.3152

1207.9590

670.7802

745.5184

848.0498

354.8297

414.4860

471.9696

-175.7656

89.9061

139.6369

F-MIN30

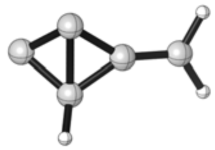

M08HX/6-31+G(d,p) = -191.431709

M08HX/6-31+G(d,p)+ZPVE = -191.96516

CCSD(T)-F12/cc-pVTZ-F12//M08HX/6-31+G(d,p) = -191.188387

Cartesian coordinates

C 0.544942 0.015301 0.040277

C 1.863427 0.027669 -0.082183

C -0.684281 -0.780539 0.029403

C -0.649327 0.809518 0.262782

C -1.732267 0.199625 -0.301915

H -0.879133 -1.697871 0.593885

H 2.415817 0.961211 -0.006674

H 2.408354 -0.892778 -0.277403

Frequencies

3133.7244

3180.0789

3279.8947

1398.7623

1437.9206

1839.7364

1007.8394

1102.5265

1123.3818

834.3414

889.3809

944.6225

672.2985

699.7734

790.5383

254.1537

331.6750

589.0073

F-TS22

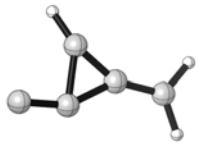

M08HX/6-31+G(d,p) = -191.431709

M08HX/6-31+G(d,p)+ZPVE = -191.954583

CCSD(T)-F12/cc-pVTZ-F12//M08HX/6-31+G(d,p) = -191.188387

Cartesian coordinates

C -2.003759 -0.464076 -0.020228

C -0.719534 -0.542047 0.008042

C 0.603503 0.069995 0.013531

C -0.453870 0.961355 0.071446

C 1.908557 -0.224626 -0.016842

H 2.645870 0.573292 -0.004310

H 2.238911 -1.258316 -0.049456

H -0.894170 1.881420 -0.281925

Frequencies

3192.3525

3299.5023

3331.3300

1440.4945

1695.7822

1923.4792

939.9079

1067.9613

1106.9916

714.9008

853.0015

858.4163

367.5982

464.5389

668.5426

-243.1597

203.6723

273.4133

F-PR32frag1

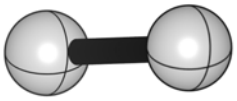

M08HX/6-31+G(d,p) = -191.431709

M08HX/6-31+G(d,p)+ZPVE = -75.885448

CCSD(T)-F12/cc-pVTZ-F12//M08HX/6-31+G(d,p) = -75.801507

Cartesian coordinates

C 0.000000 -0.000000 0.653923

C 0.000000 0.000000 -0.653923

Frequencies

1724.2850

F-PR32frag2

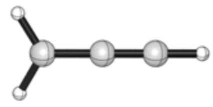

M08HX/6-31+G(d,p) = -191.431709

M08HX/6-31+G(d,p)+ZPVE = -115.928075

CCSD(T)-F12/cc-pVTZ-F12//M08HX/6-31+G(d,p) = -75.801507

Cartesian coordinates

C 0.000000 -0.000000 0.118715

C 0.000000 -0.000000 1.343613

C -0.000000 0.000000 -1.260382

H 0.000000 0.935082 -1.812603

H -0.000000 -0.935082 -1.812603

H 0.000000 -0.000000 2.413527

Frequencies

3181.8838

3285.2118

3475.1134

1077.5810

1452.7039

2033.9843

663.2955

694.8497

1030.8891

364.4945

421.9259

555.6221

F-MIN10

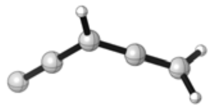

M08HX/6-31+G(d,p) = -191.431709

M08HX/6-31+G(d,p)+ZPVE = -191.986384

CCSD(T)-F12/cc-pVTZ-F12//M08HX/6-31+G(d,p) = -75.801507

Cartesian coordinates

C -1.323088 0.357572 -0.000000

C -2.542000 -0.040280 -0.000000

C 2.001868 -0.921322 0.000000

C -0.000000 0.798187 -0.000000

C 1.018810 -0.075678 0.000000

H 2.420421 -1.289834 -0.937975

H 2.420421 -1.289834 0.937975

H 0.225618 1.868797 -0.000000

Frequencies

3141.9234

3143.2678

3229.5208

1418.7073

1990.8245

2032.9735

986.8604

1101.2926

1334.4494

858.8405

886.9745

960.9549

333.0120

528.4355

600.1927

135.4704

239.2921

331.2163

F-TS41

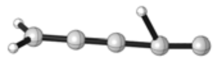

M08HX/6-31+G(d,p) = -191.431709

M08HX/6-31+G(d,p)+ZPVE = -191.925407

CCSD(T)-F12/cc-pVTZ-F12//M08HX/6-31+G(d,p) = -75.801507

Cartesian coordinates

C 1.502415 0.000009 0.084661

C 2.792503 0.000000 0.013709

C -1.125888 0.000002 0.028469

C 0.140303 0.000014 0.052538

C -2.454582 -0.000002 -0.001755

H -3.014194 -0.935128 -0.011215

H -3.014193 0.935127 -0.011373

H 0.899880 -0.000132 -1.043147

Frequencies

2245.5900

3144.6063

3241.3958

1488.0884

1906.0262

2032.9836

883.2222

1016.0458

1333.4039

490.3835

536.6980

741.3782

301.0744

348.7950

451.1441

-1204.1698

145.7038

168.7392

F-PR31frag1

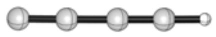

M08HX/6-31+G(d,p) = -191.431709

M08HX/6-31+G(d,p)+ZPVE = -152.697902

CCSD(T)-F12/cc-pVTZ-F12//M08HX/6-31+G(d,p) = -75.801507

Cartesian coordinates

C -0.000010 -0.004971 0.567377

C -0.000038 -0.000419 1.793504

C 0.000015 -0.004111 -0.775369

C 0.000044 0.008261 -2.063027

H -0.000062 0.007441 2.865093

Frequencies

1905.1874

2123.0076

3457.9406

633.0491

768.3150

925.5353

206.7030

512.5431

566.7881

F-PR31frag2

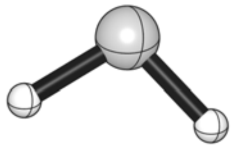

UM08HX/6-31+G(d,p) = -39.127031

UM08HX/6-31+G(d,p)+ZPVE = -39.107896

UCCSD(T)-F12/cc-pVTZ-F12//M08HX/6-31+G(d,p) = -39.075011

Cartesian coordinates

C 0.000000 0.000000 -0.176097

H 0.000000 0.866702 0.528290

H 0.000000 -0.866702 0.528290

Frequencies

1402.9010

2943.0663

3027.2733

F-MIN18

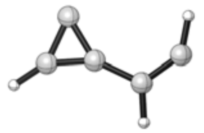

M08HX/6-31+G(d,p) = -39.124693

M08HX/6-31+G(d,p)+ZPVE = -191.976824

CCSD(T)-F12/cc-pVTZ-F12//M08HX/6-31+G(d,p) = -75.801507

Cartesian coordinates

C -1.357851 -0.797370 0.000000

C -0.246401 -1.671502 0.000000

C 0.917499 0.772215 -0.000000

C 0.000000 -0.359180 0.000000

C 0.428693 1.999933 -0.000000

H -0.563310 2.444142 -0.000000

H 1.994060 0.585648 -0.000000

H 0.117607 -2.694362 0.000000

Frequencies

3158.4226

3252.0149

3259.0762

1341.3621

1647.2358

1784.8080

941.7233

1199.0933

1252.4152

889.8808

916.6246

919.5609

476.1044

748.5587

756.5239

102.1441

195.6859

387.5680

F-TS56

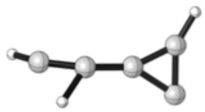

M08HX/6-31+G(d,p) = -39.124693

M08HX/6-31+G(d,p)+ZPVE = -191.913853

CCSD(T)-F12/cc-pVTZ-F12//M08HX/6-31+G(d,p) = -75.801507

Cartesian coordinates

C 1.611523 -0.807051 -0.138753

C 1.610897 0.572594 0.013072

C -0.970493 -0.013113 0.188520

C 0.393951 -0.021863 0.005698

C -2.245560 0.127003 -0.034364

H -1.653164 -0.949954 0.589298

H -2.903194 0.297090 -0.894112

H 2.154448 1.507441 0.099779

Frequencies

2394.5383

3113.9299

3263.1418

1300.6854

1652.8535

1934.5559

872.1189

969.0597

1096.8285

660.8206

727.4561

836.5876

358.1631

481.0696

610.0136

-1565.7602

178.3937

196.6327

F-MIN6

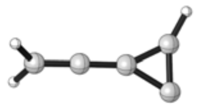

M08HX/6-31+G(d,p) = -39.124693

M08HX/6-31+G(d,p)+ZPVE = -191.992691

CCSD(T)-F12/cc-pVTZ-F12//M08HX/6-31+G(d,p) = -75.801507

Cartesian coordinates

C 1.664041 -0.817052 0.078199

C 1.645960 0.559029 -0.060727

C -0.925688 0.004514 -0.000020

C 0.416671 -0.034347 0.008033

C -2.229682 0.025609 -0.002979

H -2.803002 -0.457330 -0.799250

H -2.794530 0.523308 0.790158

H 2.169723 1.507500 -0.125942

Frequencies

3106.9683

3185.7887

3258.0540

1398.5938

1614.9488

1896.2796

959.8433

1031.5027

1181.8866

789.6322

874.0889

941.1495

397.4210

541.1950

708.7679

160.2242

198.1453

302.4708

F-TS13

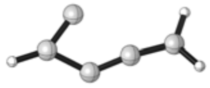

M08HX/6-31+G(d,p) = -39.124693

M08HX/6-31+G(d,p)+ZPVE = -191.970959

CCSD(T)-F12/cc-pVTZ-F12//M08HX/6-31+G(d,p) = -75.801507

Cartesian coordinates

C -0.458270 -0.723938 -0.123586

C -1.724036 -0.117879 0.072666

C 2.021067 0.101966 0.038224

C 0.728171 -0.232637 -0.036205

C -0.959282 0.970912 0.001792

H 2.341685 1.101960 -0.240718

H 2.754700 -0.621817 0.384893

H -2.742280 -0.470681 0.138476

Frequencies

3177.6929

3281.9796

3334.8251

1441.4490

1590.9974

1838.4113

985.9952

1062.7440

1278.6852

701.8541

749.5996

900.6696

408.5964

440.5317

630.0030

-234.5572

215.3878

307.2262

F-MIN16

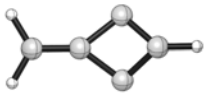

M08HX/6-31+G(d,p) = -39.124693

M08HX/6-31+G(d,p)+ZPVE = -191.990317

CCSD(T)-F12/cc-pVTZ-F12//M08HX/6-31+G(d,p) = -75.801507

Cartesian coordinates

C 0.373562 -1.563802 -0.000000

C -0.111787 -0.617079 0.889653

C -0.104258 0.550913 0.000000

C -0.111787 -0.617079 -0.889653

C -0.111787 1.875127 0.000000

H -0.104043 2.426070 -0.938227

H -0.104043 2.426070 0.938227

H 0.604426 -2.620621 -0.000000

Frequencies

3175.5346

3276.3854

3311.0844

1424.3314

1498.5104

1825.5636

993.6756

1055.4047

1167.8823

854.4684

919.6186

980.3622

677.9806

733.1397

791.4269

266.3026

338.4171

640.3039

F-TS20

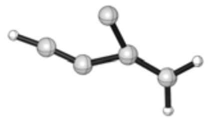

M08HX/6-31+G(d,p) = -39.124693

M08HX/6-31+G(d,p)+ZPVE = -191.957126

CCSD(T)-F12/cc-pVTZ-F12//M08HX/6-31+G(d,p) = -75.801507

Cartesian coordinates

C -1.112757 0.620466 -1.410270

C -1.417118 1.309937 -2.384003

C -0.147825 -0.139479 0.896929

C -0.311646 2.011673 -0.602727

C -0.436500 0.725480 -0.142358

H -0.459037 -1.176943 0.855781

H 0.395509 0.224507 1.762056

H -1.725912 1.824467 -3.272224

Frequencies

3202.3218

3316.7780

3463.7731

1415.8355

1490.3046

2066.0076

866.1228

1016.0953

1182.5660

556.1921

724.5380

795.6298

453.6201

521.2676

527.6478

-259.7141

264.3039

349.2303

F-PR3frag2

M08HX/6-31+G(d,p) = -1.173503

M08HX/6-31+G(d,p)+ZPVE = -1.16333

CCSD(T)-F12/cc-pVTZ-F12//M08HX/6-31+G(d,p) = -1.174044

Cartesian coordinates

H 0.000000 -0.000000 0.371363

H 0.000000 -0.000000 -0.371363

Frequencies

4465.6714

F-PR3-vdw

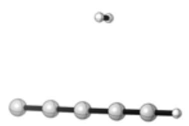

M08HX/6-31+G(d,p) = -1.173503

M08HX/6-31+G(d,p)+ZPVE = -1.16333

CCSD(T)-F12/cc-pVTZ-F12//M08HX/6-31+G(d,p) = -1.174044

Cartesian coordinates

C -1.428090 -0.143733 0.000517

C -0.164240 -0.192400 -0.000518

C 2.397950 -0.257423 -0.000743

C -2.746585 -0.098240 0.000918

C 1.175368 -0.228933 -0.000584

H 3.469514 -0.283818 -0.001125

H 0.610551 2.887427 0.370315

H 0.513519 2.920765 -0.366732

Frequencies

F-TS60

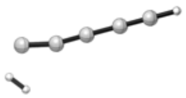

M08HX/6-31+G(d,p) = -1.173503

M08HX/6-31+G(d,p)+ZPVE = -191.901166

CCSD(T)-F12/cc-pVTZ-F12//M08HX/6-31+G(d,p) = -1.174044

Cartesian coordinates

C 0.441281 1.348342 0.000000

C 0.840242 2.500483 0.000000

C -1.106771 -2.260193 -0.000000

C -0.000000 0.070385 0.000000

C -0.390966 -1.117047 -0.000000

H -0.338221 -3.486059 -0.000000

H 0.446730 -3.279133 -0.000000

H 1.188773 3.513375 0.000000

Frequencies

2139.8142

3408.4319

3473.2150

1075.1198

1361.1722

1923.3926

725.3137

746.5468

793.1168

538.9472

585.5730

659.2090

317.2142

372.8885

393.5898

-701.7740

142.3978

151.7385

F-PR39frag2

M08HX/6-31+G(d,p) = -1.173503

M08HX/6-31+G(d,p)+ZPVE = -0.499294

CCSD(T)-F12/cc-pVTZ-F12//M08HX/6-31+G(d,p) = -1.174044

Cartesian coordinates

H 0.000000 0.000000 0.000000

F-MIN2

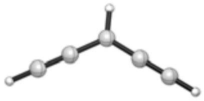

M08HX/6-31+G(d,p) = -1.173503

M08HX/6-31+G(d,p)+ZPVE = -192.049877

CCSD(T)-F12/cc-pVTZ-F12//M08HX/6-31+G(d,p) = -1.174044

Cartesian coordinates

C -0.000000 1.225874 -0.097286

C -0.000000 2.302681 0.472601

C 0.000000 -1.225874 -0.097286

C 0.000000 0.000000 -0.767327

C 0.000000 -2.302681 0.472601

H 0.000000 0.000000 -1.856645

H 0.000000 -3.246072 0.978408

H -0.000000 3.246072 0.978408

Frequencies

3192.0468

3476.5552

3480.6120

1377.8386

2074.5859

2156.2656

701.7296

967.8328

1126.5001

608.3387

688.4266

689.9499

404.6341

603.6792

607.6844

143.3041

364.2317

381.0660

F-TS44

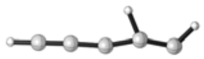

M08HX/6-31+G(d,p) = -1.173503

M08HX/6-31+G(d,p)+ZPVE = -191.921057

CCSD(T)-F12/cc-pVTZ-F12//M08HX/6-31+G(d,p) = -1.174044

Cartesian coordinates

C -1.319495 -0.299828 0.000000

C -2.538635 -0.483240 0.000000

C 1.251155 0.312735 -0.000000

C 0.000000 -0.140899 0.000000

C 2.566673 0.304839 0.000000

H 0.626785 1.305694 -0.000000

H 3.211467 1.180106 -0.000000

H -3.596435 -0.647436 0.000000

Frequencies

2524.3822

3239.1156

3470.3999

1437.7560

1663.0717

1964.3537

650.4079

685.1454

799.9801

538.4371

583.1741

593.7794

424.4108

453.3361

500.6766

-1559.0877

134.6667

163.2950

F-MIN27

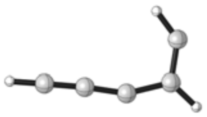

M08HX/6-31+G(d,p) = -1.173503

M08HX/6-31+G(d,p)+ZPVE = -191.953334

CCSD(T)-F12/cc-pVTZ-F12//M08HX/6-31+G(d,p) = -1.174044

Cartesian coordinates

C -1.162193 0.041552 -0.000000

C -2.312124 0.536591 -0.000000

C 1.403214 -0.674934 0.000000

C -0.000000 -0.553224 0.000000

C 2.021353 0.507897 0.000000

H 1.913073 -1.638798 0.000000

H 1.683935 1.541889 0.000000

H -3.298503 0.949616 -0.000000

Frequencies

3178.6273

3250.7386

3465.1635

1450.6541

1556.1596

1729.0805

884.7655

892.4803

1265.6289

512.0765

564.7379

802.3396

430.4292

448.6678

480.5219

105.0434

169.4510

298.8658

F-TS37

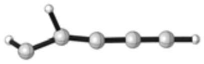

M08HX/6-31+G(d,p) = -1.173503

M08HX/6-31+G(d,p)+ZPVE = -191.928042

CCSD(T)-F12/cc-pVTZ-F12//M08HX/6-31+G(d,p) = -1.174044

Cartesian coordinates

C 1.368100 -0.016650 0.004346

C 2.599793 -0.047615 -0.030925

C -1.250603 0.166094 -0.004665

C 0.040818 0.001325 0.042785

C -2.561676 -0.293442 0.102809

H -1.682240 1.215044 0.148169

H -3.165006 0.010513 -0.774306

H 3.668658 -0.083837 -0.059970

Frequencies

2704.5645

3026.6780

3475.1835

1328.3550

1853.6849

1977.3735

755.7058

885.6875

993.4721

589.4031

599.4624

663.8272

384.7442

453.2406

542.3391

-690.7344

105.6448

166.5757

F-PR2frag1

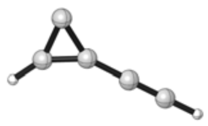

M08HX/6-31+G(d,p) = -191.459563

M08HX/6-31+G(d,p)+ZPVE = -191.41688

CCSD(T)-F12/cc-pVTZ-F12//M08HX/6-31+G(d,p) = -191.211643

Cartesian coordinates

C -0.755467 1.470976 0.000000

C 0.645093 1.651656 -0.000000

C 0.117878 -1.027875 -0.000000

C 0.000000 0.369545 -0.000000

C 0.229136 -2.231793 -0.000000

H 0.331518 -3.298490 -0.000000

H -1.751359 1.903438 0.000000

Frequencies

2250.7461

3254.9580

3474.6750

1147.0730

1314.0751

1760.6402

764.0900

892.3086

947.8821

557.0963

665.0052

713.9135

211.1684

236.3500

545.8583

F-PR2frag2

M08HX/6-31+G(d,p) = -191.459563

M08HX/6-31+G(d,p)+ZPVE = -0.499294

CCSD(T)-F12/cc-pVTZ-F12//M08HX/6-31+G(d,p) = -191.211643

Cartesian coordinates

H 0.000000 0.000000 0.000000

F-PR22frag1

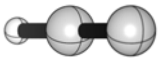

M08HX/6-31+G(d,p) = -191.459563

M08HX/6-31+G(d,p)+ZPVE = -76.569757

CCSD(T)-F12/cc-pVTZ-F12//M08HX/6-31+G(d,p) = -191.211643

Cartesian coordinates

C -0.000000 0.000000 0.732806

C 0.000000 -0.000000 -0.475059

H 0.000000 -0.000000 -1.546479

Frequencies

3476.4235

507.8749

507.8749

2130.8631

F-PR15frag2

M08HX/6-31+G(d,p) = -191.459563

M08HX/6-31+G(d,p)+ZPVE = -0.499294

CCSD(T)-F12/cc-pVTZ-F12//M08HX/6-31+G(d,p) = -191.211643

Cartesian coordinates

H 0.000000 0.000000 0.000000

F-MIN3

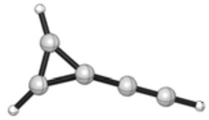

M08HX/6-31+G(d,p) = -191.459563

M08HX/6-31+G(d,p)+ZPVE = -192.011551

CCSD(T)-F12/cc-pVTZ-F12//M08HX/6-31+G(d,p) = -191.211643

Cartesian coordinates

C -0.018505 -1.100194 0.000000

C 0.046235 -2.325355 0.000000

C 0.046235 1.522859 0.659581

C -0.185955 0.254097 -0.000000

C 0.046235 1.522859 -0.659581

H 0.148955 2.073918 1.586616

H 0.148955 2.073918 -1.586616

H 0.096625 -3.393432 0.000000

Frequencies

3256.6091

3306.7734

3485.3188

1484.8835

1723.8950

2083.3012

875.2841

969.1752

1034.1769

690.1600

720.4135

812.2717

411.1099

531.9415

617.0591

80.0578

206.0383

396.2663

F-TS18

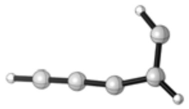

M08HX/6-31+G(d,p) = -191.992647

M08HX/6-31+G(d,p)+ZPVE = -192.4861

CCSD(T)-F12/cc-pVTZ-F12//M08HX/6-31+G(d,p) = -191.211643

Cartesian coordinates

C -1.091022 -0.014474 0.061309

C -2.303766 0.045912 -0.106683

C 1.465287 -0.598240 -0.054385

C 0.242407 -0.136202 0.335579

C 1.768145 0.721019 -0.149520

H 1.937506 -1.534865 -0.355980

H 0.935762 1.423752 0.151110

H -3.359570 0.003023 -0.312928

Frequencies

3191.5779

3271.2598

3472.3485

1357.7723

1624.8052

1810.1650

846.4122

888.5266

1238.0641

507.8277

578.9095

689.0780

441.1585

449.4440

478.7210

-547.1360

138.6246

164.4139

F-PR29frag1

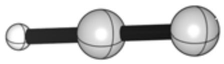

M08HX/6-31+G(d,p) = -191.459563

M08HX/6-31+G(d,p)+ZPVE = -76.569757

CCSD(T)-F12/cc-pVTZ-F12//M08HX/6-31+G(d,p) = -191.211643

Cartesian coordinates

C -0.000000 0.000000 0.732806

C 0.000000 -0.000000 -0.475059

H 0.000000 -0.000000 -1.546479

Frequencies

3476.4235

507.8749

507.8749

2130.8631

F-PR29frag2

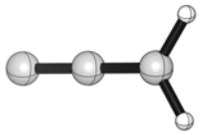

M08HX/6-31+G(d,p) = -115.300224

M08HX/6-31+G(d,p)+ZPVE = -115.269005

CCSD(T)-F12/cc-pVTZ-F12//M08HX/6-31+G(d,p) = -191.211643

Cartesian coordinates

C -0.000000 0.000000 1.125066

C 0.000000 -0.000000 -1.490224

C 0.000000 -0.000000 -0.199706

H -0.000000 -0.933809 1.694592

H -0.000000 0.933809 1.694592

Frequencies

2075.0598

3109.8578

3201.6372

1044.9850

1166.7798

1474.6035

283.6150

302.8413

1044.4031

F-PR54frag2

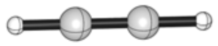

M08HX/6-31+G(d,p) = -77.310021

M08HX/6-31+G(d,p)+ZPVE = -77.282823

CCSD(T)-F12/cc-pVTZ-F12//M08HX/6-31+G(d,p) = -77.216169

Cartesian coordinates

C -0.000000-0.000000 0.602948

C 0.000000-0.000000 -0.602948

H -0.000000-0.000000 1.673949

H 0.000000-0.000000 -1.673949

Frequencies

3531.2598

769.8186

2098.0619

3426.5641

671.7125

671.7125

769.8186

I-PR52frag1

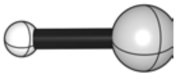

M08HX/6-31+G(d,p) = -77.310021

M08HX/6-31+G(d,p)+ZPVE = -38.465932

CCSD(T)-F12/cc-pVTZ-F12//M08HX/6-31+G(d,p) = -77.216169

Cartesian coordinates

C -0.0000000.000000 0.160716

H 0.000000-0.000000 -0.964294

Frequencies

2874.8518

I-PR52frag2

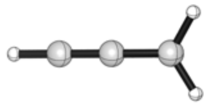

M08HX/6-31+G(d,p) = -77.310021

M08HX/6-31+G(d,p)+ZPVE = -115.928075

CCSD(T)-F12/cc-pVTZ-F12//M08HX/6-31+G(d,p) = -77.216169

Cartesian coordinates

C -0.000000 0.000000 -1.343614

C -0.000000 0.000000 -0.118717

C 0.000000 -0.000000 1.260382

H -0.000000 0.000000 -2.413529

H -0.000000 -0.935075 1.812612

H -0.000000 0.935075 1.812612

Frequencies

3181.9051

3285.2282

3475.0984

1077.5756

1452.7049

2033.9909

663.3084

694.8434

1030.8785

364.4899

421.9284

555.6382

I-MIN20

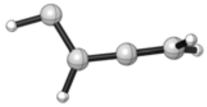

M08HX/6-31+G(d,p) = -154.59219

M08HX/6-31+G(d,p)+ZPVE = -154.534396

CCSD(T)-F12/cc-pVTZ-F12//M08HX/6-31+G(d,p) = -154.388856

Cartesian coordinates

C 0.0728661.799005 0.000000

C -0.7025910.581975 -0.000000

C -0.000000-0.556119 0.000000

C 0.702907-1.646445 0.000000

H -0.6623252.636176 -0.000000

H -1.7973910.504514 -0.000000

H 1.010310-2.105594 0.939224

H 1.010310-2.105594 -0.939224

Frequencies

3097.8316

3161.4436

3250.9632

1435.0755

2011.1930

2962.2997

1147.9427

1215.7713

1358.4730

926.8914

977.8347

992.5650

578.3129

597.3587

854.7131

135.0739

218.8800

445.8426

I-TS13

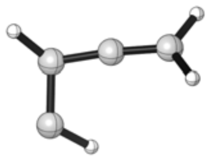

M08HX/6-31+G(d,p) = -154.590826

M08HX/6-31+G(d,p)+ZPVE = -154.532894

CCSD(T)-F12/cc-pVTZ-F12//M08HX/6-31+G(d,p) = -154.394922

Cartesian coordinates

C -1.471769 -0.661601 0.040386

C -0.907696 0.609342 -0.033467

C 0.399032 0.283189 -0.031788

C 1.635025 -0.140508 0.033169

H -0.904424 -1.446689 -0.490106

H -1.353480 1.534114 0.332414

H 2.403450 0.332528 -0.579529

H 1.926896 -0.962484 0.687422

Frequencies

3146.2064

3189.0732

3232.9429

1416.6588

1952.7476

3072.3400

1072.8521

1265.7425

1356.4264

920.0565

970.4152

993.9504

574.1138

758.5725

843.9271

-308.3748

273.8988

389.2998

I-MIN3

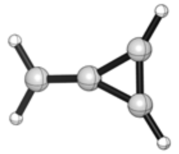

M08HX/6-31+G(d,p) = -154.670965

M08HX/6-31+G(d,p)+ZPVE = -154.609897

CCSD(T)-F12/cc-pVTZ-F12//M08HX/6-31+G(d,p) = -154.463954

Cartesian coordinates

C -0.000000 -0.000000 -1.594337

C -0.000000 -0.000000 -0.262208

C 0.000000 0.660462 1.020501

C 0.000000 -0.660462 1.020501

H -0.000000 0.934551 -2.149572

H 0.000000 -0.934551 -2.149572

H 0.000000 -1.578606 1.596205

H -0.000000 1.578606 1.596205

Frequencies

3264.4859

3272.0436

3306.9574

1635.9757

1861.8074

3178.1013

1057.7495

1139.6372

1445.3401

871.0136

909.7177

1027.1368

705.0268

794.6405

851.9832

356.2724

436.5832

691.4001

I-TS47

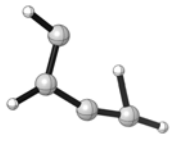

M08HX/6-31+G(d,p) = -154.530497

M08HX/6-31+G(d,p)+ZPVE = -154.477677

CCSD(T)-F12/cc-pVTZ-F12//M08HX/6-31+G(d,p) = -154.330993

Cartesian coordinates

C 1.116174-1.251673 0.000000

C -0.000000 -0.704780 0.000000

C -0.950908 0.340640 -0.000000

C -0.213371 1.502477 -0.000000

H 1.988972 -1.873327 0.000000

H -2.022225 0.131404 -0.000000

H 1.189245 0.026188 0.000000

H -0.867363 2.395747 -0.000000

Frequencies

3026.8981

3164.8879

3453.4796

1419.0311

1859.8219

2073.5600

971.4361

1086.2064

1281.0224

646.0211

828.7213

965.0855

447.1342

606.8951

625.7805

-763.1242

300.7808

428.3845

I-TS35

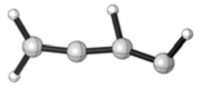

M08HX/6-31+G(d,p) = -154.552381

M08HX/6-31+G(d,p)+ZPVE = -154.498305

CCSD(T)-F12/cc-pVTZ-F12//M08HX/6-31+G(d,p) = -154.355684

Cartesian coordinates

C 2.027476 -0.253582 0.065643

C 0.745978 0.078554 -0.083577

C -0.57882 9-0.086169 -0.021498

C -1.88647 0-0.023903 0.043996

H 2.702899 0.606029 0.181533

H 0.335498 1.146660 -0.364375

H -2.464586 -0.923992 -0.168983

H -2.422738 0.881904 0.324438

Frequencies

3105.0375

3148.0324

3236.3614

1568.1398

1964.6836

2502.4694

944.8521

1002.1779

1377.8021

826.0507

861.6156

895.5646

460.3721

568.9269

790.1488

-1017.7375

224.2419

260.3428

I-MIN1

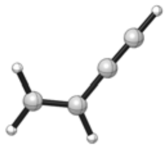

M08HX/6-31+G(d,p) = -154.697846

M08HX/6-31+G(d,p)+ZPVE = -154.636396

CCSD(T)-F12/cc-pVTZ-F12//M08HX/6-31+G(d,p) = -154.501632

Cartesian coordinates

C -0.474584 1.858700 0.000000

C 0.000000 0.745865 -0.000000

C 0.593652 -0.559125 -0.000000

C -0.113432 -1.695406 0.000000

H -0.904063 2.839398 0.000000

H 1.685358 -0.592395 -0.000000

H -1.203014 -1.685164 0.000000

H 0.387900 -2.662040 -0.000000

Frequencies

3174.1697

3264.6204

3482.5175

1699.7289

2250.8907

3162.5876

1107.3440

1313.1378

1433.5556

895.5250

968.5787

1008.9908

674.6058

694.3378

717.2275

228.7579

337.5937

559.6377

I-PR63frag1

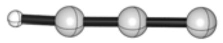

M08HX/6-31+G(d,p) = -154.697846

M08HX/6-31+G(d,p)+ZPVE = -114.62208

CCSD(T)-F12/cc-pVTZ-F12//M08HX/6-31+G(d,p) = -154.501632

Cartesian coordinates

C 0.022782 -0.056248 -1.152167

C -0.00113 10.003075 0.088171

C -0.029037 0.062917 1.434704

H 0.044318 -0.058464 -2.224244

Frequencies

1162.8658

1899.1805

3450.9669

238.5580

336.8779

388.3484

I-PR63frag2

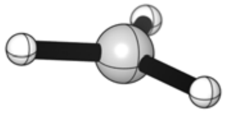

M08HX/6-31+G(d,p) = -154.697846

M08HX/6-31+G(d,p)+ZPVE = -39.798649

CCSD(T)-F12/cc-pVTZ-F12//M08HX/6-31+G(d,p) = -154.501632

Cartesian coordinates

C 0.000000 0.000000 0.000000

H 0.000000 1.083469 -0.000000

H 0.938312 -0.541734 -0.000000

H -0.938312 -0.541734 -0.000000

Frequencies

3147.3760

3331.7210

3331.7486

532.9560

1410.2310

1410.2356

I-MIN16

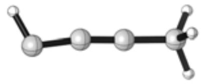

M08HX/6-31+G(d,p) = -154.601803

M08HX/6-31+G(d,p)+ZPVE = -154.544527

CCSD(T)-F12/cc-pVTZ-F12//M08HX/6-31+G(d,p) = -154.402166

Cartesian coordinates

C -2.168523 -0.161585 0.003273

C -0.811267 0.012089 -0.002670

C 0.422500 0.000657 -0.013324

C 1.873414 0.009666 -0.003964

H 2.291192 -0.858572 -0.530421

H 2.292897 0.934999 -0.419279

H 2.198970 -0.061009 1.044909

H -2.679803 0.819622 0.004904

Frequencies

3034.1279

3110.9807

3123.9784

1478.0762

2148.3729

3030.2096

1290.5820

1395.5039

1444.3167

967.2318

1019.7673

1053.5235

373.6468

443.4148

786.9188

80.5656

169.8135

190.1106

I-TS17

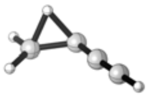

M08HX/6-31+G(d,p) = -154.582789

M08HX/6-31+G(d,p)+ZPVE = -154.526797

CCSD(T)-F12/cc-pVTZ-F12//M08HX/6-31+G(d,p) = -154.384359

Cartesian coordinates

C -1.901765 0.171696 0.015176

C -0.727475 -0.139927 -0.015863

C 0.604536 -0.617542 -0.115940

C 1.613224 0.341757 -0.033866

H -2.938836 0.435892 0.042514

H 1.311407 -0.422017 0.970203

H 1.440196 1.428662 0.002971

H 2.656113 0.021557 -0.112721

Frequencies

3041.9335

3170.8649

3480.8368

1533.0732

2184.9881

2219.0071

1122.2570

1319.8499

1419.5473

757.3475

900.1820

1054.4602

551.2092

567.6160

739.7027

-1122.8163

219.3479

295.2649

I-TS50

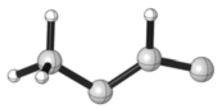

M08HX/6-31+G(d,p) = -154.526874

M08HX/6-31+G(d,p)+ZPVE = -154.471599

CCSD(T)-F12/cc-pVTZ-F12//M08HX/6-31+G(d,p) = -154.320295

Cartesian coordinates

C 2.076247 -0.072411 0.016498

C 0.794419 0.201620 -0.011418

C -0.380495 -0.649931 -0.033115

C -1.633683 0.141549 -0.007963

H 0.801078 1.323242 -0.029799

H -1.882929 0.148756 1.073163

H -1.589559 1.191803 -0.343707

H -2.467516 -0.388757 -0.483670

Frequencies

2969.8442

3044.4953

3126.6682

1494.0971

1760.7687

2890.2237

1184.8511

1348.0075

1407.4552

849.8117

979.6997

1123.8193

390.3880

641.7516

775.6512

-266.7425

108.0539

167.4221

I-PR68frag1

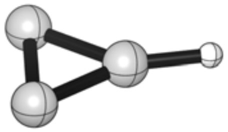

M08HX/6-31+G(d,p) = -154.526874

M08HX/6-31+G(d,p)+ZPVE = -114.629842

CCSD(T)-F12/cc-pVTZ-F12//M08HX/6-31+G(d,p) = -154.320295

Cartesian coordinates

C 0.674701 0.557068 0.000000

C 0.000000 -0.700736 0.000000

C -0.697148 0.440177 -0.000000

H 0.134680 -1.779052 0.000000

Frequencies

1227.9047

1661.9671

3251.9059

678.5999

853.4191

890.4773

I-PR68frag2

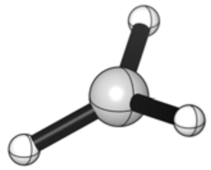

M08HX/6-31+G(d,p) = -154.526874

M08HX/6-31+G(d,p)+ZPVE = -39.798649

CCSD(T)-F12/cc-pVTZ-F12//M08HX/6-31+G(d,p) = -154.320295

Cartesian coordinates

C 0.000000 0.000000 0.000000

H 0.000000 1.083469 -0.000000

H 0.938312 -0.541734 -0.000000

H -0.938312 -0.541734 -0.000000

Frequencies

3147.3760

3331.7210

3331.7486

532.9560

1410.2310

1410.2356

I-MIN4

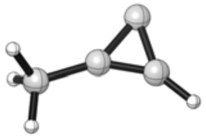

M08HX/6-31+G(d,p) = -154.651289

M08HX/6-31+G(d,p)+ZPVE = -154.590489

CCSD(T)-F12/cc-pVTZ-F12//M08HX/6-31+G(d,p) = -154.444703

Cartesian coordinates

C 1.135906 0.802340 -0.000000

C -0.203176 1.260871 0.000000

C -0.000000 -0.056098 -0.000000

C -0.490748 -1.449281 -0.000000

H -0.883705 2.107569 0.000000

H -0.092149 -1.969103 0.881107

H -1.583889 -1.516353 0.000000

H -0.092149 -1.969103 -0.881107

Frequencies

3132.4275

3154.4183

3244.9125

1470.5159

1807.9307

3048.7558

1340.7547

1390.7548

1469.5091

1010.9288

1032.4628

1225.6081

773.6092

882.4566

930.0455

57.4483

338.2350

377.7165

I-TS85

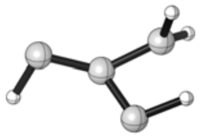

M08HX/6-31+G(d,p) = -154.493539

M08HX/6-31+G(d,p)+ZPVE = -154.438817

CCSD(T)-F12/cc-pVTZ-F12//M08HX/6-31+G(d,p) = -154.293403

Cartesian coordinates

C 0.444293 -1.102312 0.053122

C 1.067489 0.590156 -0.006305

C -0.276712 0.017007 -0.146867

C -1.623071 0.369033 0.111809

H 1.597972 -0.720212 0.186292

H 1.397104 1.048089 0.929982

H -2.247552 -0.467463 -0.267122

H 1.580478 0.896289 -0.919703

Frequencies

3016.5656

3105.2811

3217.9166

1467.2812

1629.6919

2408.0472

1098.3816

1134.9601

1291.2668

935.6084

1021.2951

1051.4844

498.0102

679.9611

825.3316

-889.1088

247.8015

391.4638

REFERENCES AND NOTES

1. J. Cernicharo, A. M. Heras, A. G. G. M. Tielens, J. R. Pardo, F. Herpin, M. Guélin, L. B. F. M. Waters, Infrared space observatory's discovery of C_4H_2, C_6H_2, and benzene in CRL 618, *Astrophys. J.* **546**, L123–L126 (2001).

2. J. Cernicharo, M. Agúndez, R. I. Kaiser, C. Cabezas, B. Tercero, N. Marcelino, J. R. Pardo, P. de Vicente, Discovery of benzyne, *o*-C_6H_4, in TMC-1 with the QUIJOTE line survey, *Astron. Astrophys.* **652**, L9 (2021).

3. J. Cernicharo, M. Agúndez, C. Cabezas, B. Tercero, N. Marcelino, J. R. Pardo, P. de Vicente, Pure hydrocarbon cycles in TMC-1: Discovery of ethynyl cyclopropenylidene, cyclopentadiene, and indene, *Astron. Astrophys.* **649**, L15 (2021).

4. A. M. Burkhardt, K. L. K. Lee, P. B. Changala, C. N. Shingledecker, I. R. Cooke, R. A. Loomis, H. Wei, S. B. Charnley, E. Herbst, M. C. McCarthy, B. A. McGuire, Discovery of the pure polycyclic aromatic hydrocarbon indene (c-C_9H_8) with GOTHAM observations of TMC-1, *Astrophys. J. Lett.* **913**, L18 (2021).

5. M. L. Sita, P. B. Changala, C. Xue, A. M. Burkhardt, C. N. Shingledecker, K. L. K. Lee, R. A. Loomis, E. Momjian, M. A. Siebert, D. Gupta, E. Herbst, A. J. Remijan, M. C. McCarthy, I. R. Cooke, B. A. McGuire, Discovery of interstellar 2-cyanoindene (2-C_9H_7CN) in GOTHAM observations of TMC-1, *Astrophys. J. Lett.* **938**, L12 (2022).

6. A. G. G. M. Tielens, Interstellar polycyclic aromatic hydrocarbon molecules, *Annu. Rev. Astron. Astrophys.* **46**, 289–337 (2008).

7. C. Joblin, G. Mulas, Interstellar polycylic aromatic hydrocarbons: From space to the laboratory, *Eur. Astron. Soc. Publ. Ser.* **35**, 133–152 (2009).

8. Z. D. Levey, B. A. Laws, S. P. Sundar, K. Nauta, S. H. Kable, G. da Silva, J. F. Stanton, T. W. Schmidt, PAH growth in flames and space: Formation of the phenalenyl radical, *J. Phys. Chem. A* **126**, 101–108 (2022).

9. B. A. McGuire, R. A. Loomis, A. M. Burkhardt, K. L. K. Lee, C. N. Shingledecker, S. B. Charnley, I. R. Cooke, M. A. Cordiner, E. Herbst, S. Kalenskii, M. A. Siebert, E. R. Willis, C. Xue, A. J. Remijan, M. C. McCarthy, Detection of two interstellar polycyclic aromatic hydrocarbons via spectral matched filtering, *Science* **371**, 1265–1269 (2021).

10. E. Reizer, B. Viskolcz, B. Fiser, Formation and growth mechanisms of polycyclic aromatic hydrocarbons: A mini-review, *Chemosphere* **291**, 132793 (2022).

11. X. Gu, R. I. Kaiser, Reaction dynamics of phenyl radicals in extreme environments: A crossed molecular beam study, *Acc. Chem. Res.* **42**, 290–302 (2009).

12. R. I. Kaiser, D. S. N. Parker, F. Zhang, A. Landera, V. V. Kislov, A. M. Mebel, Pah formation under single collision conditions: Reaction of phenyl radical and 1,3-butadiene to form 1,4-dihydronaphthalene, *J. Phys. Chem. A* **116**, 4248–4258 (2012).

13. R. I. Kaiser, M. Goswami, P. Maksyutenko, F. Zhang, Y. S. Kim, A. Landera, A. M. Mebel, A crossed molecular beams and ab initio study on the formation of C_6H_3 radicals. An interface between resonantly stabilized and aromatic radicals. *J. Phys. Chem. A* **115**, 10251–10258 (2011).

14. A. V. Kelleghan, A. S. Bulger, D. C. Witkowski, N. K. Garg, Strain-promoted reactions of 1,2,3-cyclohexatriene and its derivatives, *Nature* **618**, 748–754 (2023).

15. J. A. Miller, S. J. Klippenstein, The recombination of propargyl radicals and other reactions on a C_6H_6 potential. *J. Phys. Chem. A* **107**, 7783–7799 (2003).

16. M. Agúndez, C. Cabezas, B. Tercero, N. Marcelino, J. D. Gallego, P. de Vicente, J. Cernicharo, Discovery of the propargyl radical (CH_2CCH) in TMC-1: One of the most abundant radicals ever found and a key species for cyclization to benzene in cold dark clouds. *Astron. Astrophys.* **647**, L10 (2021).

17. J. Cernicharo, M. Agúndez, C. Cabezas, N. Marcelino, B. Tercero, J. R. Pardo, J. D. Gallego, F. Tercero, J. A. López-Pérez, P. de Vicente, Discovery of CH_2CHCCH and detection of HCCN, HC_4N, CH_3CH_2CN, and, tentatively, CH_3CH_2CCH in TMC-1. *Astron. Astrophys.* **647**, L2 (2021).

18. A. M. Mebel, V. V. Kislov, R. I. Kaiser, Ab initio/Rice-Ramsperger-Kassel-Marcus study of the singlet C_4H_4 potential energy surface and of the reactions of C_2 ($X^1\Sigma_g^+$) with C_4H_4 ($X^1A_{1g}^+$) and C (^{1}D) with C_3H_4 (allene and methylacetylene), *J. Chem. Phys.* **125**, 133113 (2006).

19. C. Puzzarini, Gas-phase chemistry in the interstellar medium: The role of laboratory astrochemistry, *Front. Astron. Space Sci.* **8**, 811342 (2022).

20. M. H. Stockett, J. N. Bull, H. Cederquist, S. Indrajith, M. Ji, J. E. Navarro Navarrete, H. T. Schmidt, H. Zettergren, B. Zhu, Efficient stabilization of cyanonaphthalene by fast radiative cooling and implications for the resilience of small pahs in interstellar clouds, *Nat. Commun.* **14**, 395 (2023).

21. M. Saito, H. Kubota, K. Yamasa, K. Suzuki, T. Majima, H. Tsuchida, Direct measurement of recurrent fluorescence emission from naphthalene ions, *Phys. Rev. A* **102**, 012820 (2020).

22. E. Martínez-Núñez, An automated method to find transition states using chemical dynamics simulations, *J. Comput. Chem.* **36**, 222–234 (2015).

23. C. W. Gao, J. W. Allen, W. H. Green, R. H. West, Reaction mechanism generator: Automatic construction of chemical kinetic mechanisms, *Comput. Phys. Commun.* **203**, 212–225 (2016).

24. P. L. Bhoorasigh, B. L. Slakman, F. S. Khanshan, J. Y. Cain, R. H. West, Automated transition state theory calculations for high-throughput kinetics, *J. Phys. Chem. A* **121**, 6896–6904 (2017).

25. C. Cavallotti, M. Pelucchi, Y. Georgievskii, S. J. Klippenstein, EStokTP: Electronic structure to temperature-and pressure-dependent rate constants - A code for automatically predicting the thermal kinetics of reactions, *J. Chem. Theory Comput.* **15**, 1122–1145 (2019).

26. I. Ismail, H. B. V. A. Stuttaford-Fowler, C. Ochan Ashok, C. Robertson, S. Habershon, Automatic proposal of multistep reaction mechanisms using a graph-driven search, *J. Phys. Chem. A* **123**, 3407–3417 (2019).

27. R. Van de Vijver, J. Zádor, Kinbot: Automated stationary point search on potential energy surfaces, *Comput. Phys. Commun.* **248**, 106947 (2020).

28. E. Martínez-Núñez, G. L. Barnes, D. R. Glowacki, S. Kopec, D. Peláez, A. Rodríguez, R. Rodríguez-Fernández, R. J. Shannon, J. J. P. Stewart, P. G. Tahoces, S. A. Vazquez, AutoMeKin2021: An open-source program for automated reaction discovery, *J. Comput. Chem.* **42**, 2036–2048 (2021).

29. C. Robertson, R. Hyland, A. J. D. Lacey, S. Havens, S. Habershon, Identifying barrierless mechanisms for benzene formation in the interstellar medium using permutationally invariant reaction discovery, *J. Chem. Theory Comput.* **17**, 2307–2322 (2021).

30. J. Zádor, C. Martí, R. Van de Vijver, S. L. Johansen, Y. Yang, H. A. Michelsen, H. N. Najm, Automated reaction kinetics of gas-phase organic species over multiwell potential energy surfaces. *J. Phys. Chem. A* **127**, 565–588 (2023).

31. E. Martínez-Núñez, An automated transition state search using classical trajectories initialized at multiple minima. *Phys. Chem. Chem. Phys.* **17**, 14912–14921 (2015).

32. D. Garay-Ruiz, M. Álvarez Moreno, C. Bo, E. Martínez-Núñez, New tools for taming complex reaction networks: The unimolecular decomposition of indole revisited. *ACS Phys. Chem. Au* **2**, 225–236 (2022).

33. G. Henkelman, H. Jónsson, Improved tangent estimate in the nudged elastic band method for finding minimum energy paths and saddle points. *J. Chem. Phys.* **113**, 9978–9985 (2000).

34. M. Castineira Reis, E. Martinez-Nunez, A. Fernandez-Ramos, Comprehensive computational automated search of barrierless reactions leading to the formation of benzene and other C6-membered rings (2024). https://zenodo.org/records/11175641.

35. C. H. Wu, R. D. Kern, Shock-tube study of allene pyrolysis. *J. Phys. Chem.* **91**, 6291–6296 (1987).

36. J. A. Miller, C. F. Melius, Kinetic and thermodynamic issues in the formation of aromatic compounds in flames of aliphatic fuels. *Combust. Flame* **91**, 21–39 (1992).

37. L. Zhao, W. Lu, M. Ahmed, M. V. Zagidullin, V. N. Azyazov, A. N. Morozov, A. M. Mebel, R. I. Kaiser, Gas-phase synthesis of benzene via the propargyl radical self-reaction. *Sci. Adv.* **7**, eabf0360 (2021).

38. W. Tang, R. S. Tranter, K. Brezinsky, Isomeric product distributions from the self-reaction of propargyl radicals. *J. Phys. Chem. A* **109**, 6056–6065 (2005).

39. M. E. Law, P. R. Westmoreland, T. A. Cool, J. Wang, N. Hansen, C. A. Taatjes, T. Kasper, Benzene precursors and formation routes in a stoichiometric cyclohexane flame. *Proc. Combust. Inst.* **31**, 565–573 (2007).

40. E. H. Wilson, S. K. Atreya, A. Coustenis, Mechanisms for the formation of benzene in the atmosphere of Titan. *J. Geophys. Res. Planets* **108**, 5014 (2003).

41. S. Green, E. Herbst, Metastable isomers: A new class of interstellar molecules. *Astrophys. J.* **229**, 121 (1979).

42. A. Yokoyama, X. Zhao, E. J. Hintsa, R. E. Continetti, Y. T. Lee, Molecular beam studies of the photodissociation of benzene at 193 and 248 nm. *J. Chem. Phys.* **92**, 4222–4233 (1990).

43. J. M. Hollis, P. R. Jewell, F. J. Lovas, Confirmation of interstellar methylene. *Astrophys. J.* **438**, 259 (1995).

44. H. Feuchtgruber, F. P. Helmich, E. F. van Dishoeck, C. M. Wright, Detection of interstellar CH_3. *Astrophys. J.* **535**, L111–L114 (2000).

45. K. D. Tucker, M. L. Kutner, P. Thaddeus, The ethynyl radical C_2H–A new interstellar molecule. *Astrophys. J.* **193**, L115–L119 (1974).

46. J. Cernicharo, C. A. Gottlieb, M. Guélin, T. C. Killian, G. Paubert, P. Thaddeus, J. M. Vrtilek, Astronomical detection of H_2CCC. *Astrophys. J.* **368**, L39 (1991).

47. J. Cernicharo, C. Cabezas, M. Agúndez, B. Tercero, N. Marcelino, J. R. Pardo, F. Tercero, J. D. Gallego, J. A. López-Pérez, P. de Vicente, Discovery of allenyl acetylene, $H_2CCCHCCH$, in TMC-1. A study of the isomers of C_5H_4. *Astron. Astrophys.* **647**, L3 (2021).

48. J. Cernicharo, M. Guelin, C. M. Walmsley, Detection of the hyperfine structure of the C_5H radical. *Astron. Astrophys.* **172**, L5 (1987).

49. W. D. Langer, T. Velusamy, T. B. H. Kuiper, R. Peng, M. C. McCarthy, M. J. Travers, A. Kovács, C. A. Gottlieb, P. Thaddeus, First astronomical detection of the cumulene carbon chain molecule H_2C_6 in TMC-1. *Astrophys. J.* **480**, L63 (1997).

50. J. Cernicharo, C. A. Gottlieb, M. Guélin, T. C. Killian, G. Paubert, P. Thaddeus, J. M. Vrtilek, Astronomical detection of H_2CCCC. *Astrophys. J.* **368**, L43 (1991).

51. P. Thaddeus, J. M. Vrtilek, C. A. Gottlieb, P. Thaddeus, J. M. Vrtilek, C. A. Gottlieb, Laboratory and astronomical identification of cyclopropenylidene, C_3H_2. *Astrophys. J.* **299**, L63 (1985).

52. Y. Guo, X. Gu, F. Zhang, A. M. Mebel, R. I. Kaiser, A crossed molecular beam study on the formation of hexenediynyl radicals ($H_2CCCCCCH$; C_6H_3 (X^2A')) via reactions of tricarbon molecules, C_3 ($X^1\Sigma_g^+$), with allene (H2CCCH2; X^1A_1) and methylacetylene (CH_3CCH; X^1A_1). *Phys. Chem. Chem. Phys.* **9**, 1972 (2007).

53. K. W. Hinkle, J. J. Keady, P. F. Bernath, Detection of C_3 in the circumstellar shell of IRC+10216. *Science* **241**, 1319–1322 (1988).

54. C. Cabezas, M. Agúndez, R. Fuentetaja, Y. Endo, N. Marcelino, B. Tercero, J. R. Pardo, P. de Vicente, J. Cernicharo, Discovery of the cyclic C_5H radical in TMC-1. *Astron. Astrophys.* **663**, L2 (2022).

55. P. Thaddeus, C. A. Gottlieb, A. Hjalmarson, L. E. B. Johansson, W. M. Irvine, P. Friberg, R. A. Linke, Astronomical identification of the C_3H radical. *Astrophys. J., Lett. Ed.* **294**, L49–L53 (1985).

56. S. Yamamoto, S. Saito, M. Ohishi, H. Suzuki, S.-I. Ishikawa, N. Kaifu, A. Murakami, Laboratory and astronomical detection of the cyclic C_3H radical. *Astrophys. J.* **322**, L55 (1987).

57. J. G. Mangum, A. Wootten, Observations of the cyclic C_3H radical in the interstellar medium. *Astron. Astrophys.* **239**, 319 (1990).

58. R. Fuentetaja, M. Agúndez, C. Cabezas, B. Tercero, N. Marcelino, J. R. Pardo, P. de Vicente, J. Cernicharo, Discovery of two new interstellar molecules with QUIJOTE: HCCCHCCC and HCCCCS. *Astron. Astrophys.* **667**, L4 (2022).

59. J. J. P. Stewart, Optimization of parameters for semiempirical methods VI: More modifications to the NDDO approximations and re-optimization of parameters. *J. Mol. Model.* **19**, 1–32 (2013).

60. J. J. P. Stewart, “MOPAC2016,” 2016; http://openmopac.net/MOPAC2016.html; 10th May 2024.

61. Y. Zhao, D. G. Truhlar, Exploring the limit of accuracy of the global hybrid meta density functional for main-group thermochemistry, kinetics, and noncovalent interactions. *J. Chem. Theory Comput.* **4**, 1849–1868 (2008).

62. A. D. Becke, Density-functional thermochemistry. iii. the role of exact exchange. *J. Chem. Phys.* **98**, 5648–5652 (1993).

63. M. J. Frisch, G. W. Trucks, H. B. Schlegel, G. E. Scuseria, M. A. Robb, J. R. Cheeseman, G. Scalmani, V. Barone, G. A. Petersson, H. Nakatsuji, X. Li, M. Caricato, A. V. Marenich, J. Bloino, B. G. Janesko, R. Gomperts, B. Mennucci, H. P. Hratchian, J. V. Ortiz, A. F. Izmaylov, J. L. Sonnenberg, D. Williams-Young, F. Ding, F. Lipparini, F. Egidi, J. Goings, B. Peng, A. Petrone, T. Henderson, D. Ranasinghe, V. G. Zakrzewski, J. Gao, N. Rega, G. Zheng, W. Liang, M. Hada, M. Ehara, K. Toyota, R. Fukuda, J. Hasegawa, M. Ishida, T. Nakajima, Y. Honda, O. Kitao, H. Nakai, T. Vreven, K. Throssell, J. A. Montgomery, Jr., J. E. Peralta, F. Ogliaro, M. J. Bearpark, J. J. Heyd, E. N. Brothers, K. N. Kudin, V. N. Staroverov, T. A. Keith, R. Kobayashi, J. Normand, K. Raghavachari, A. P. Rendell, J. C. Burant, S. S. Iyengar, J. Tomasi, M. Cossi, J. M. Millam, M. Klene, C. Adamo, R. Cammi, J. W. Ochterski, R. L. Martin, K. Morokuma, O. Farkas, J. B. Foresman, D. J. Fox, *Gaussian 16 Revision B.01* (Gaussian Inc., 2016)..

64. H.-J. Werner, P. J. Knowles, G. Knizia, F. R. Manby, M. Schütz, Molpro: A general-purpose quantum chemistry program package. *WIREs Comput. Mol. Sci.* **2**, 242–253 (2012).

65. I. M. Alecu, J. Zheng, Y. Zhao, D. G. Truhlar, Computational thermochemistry: Scale factor databases and scale factors for vibrational frequencies obtained from electronic model chemistries. *J. Chem. Theory Comput.* **6**, 2872–2887 (2010).

66. J. L. Bao, X. Zhang, D. G. Truhlar, Barrierless association of CF_2 and dissociation of C_2F_4 by variational transition-state theory and system-specific quantum Rice–Ramsperger–Kassel theory. *Proc. Natl. Acad. Sci. U.S.A.* **113**, 13606 (2016).

67. L. G. Gao, J. Zheng, A. Fernández-Ramos, D. G. Truhlar, X. Xu, Kinetics of the methanol reaction with OH at interstellar, atmospheric, and combustion temperatures. *J. Am. Chem. Soc.* **140**, 2906–2918 (2018).

68. D. Gonzalez, A. Lema-Saavedra, S. Espinosa, E. Martínez-Núñez, A. Fernández-Ramos, A. Canosa, B. Ballesteros, E. Jiménez, Reaction of OH radicals with CH_3NH_2 in the gas phase: Experimental (11.7–177.5 K) and computed rate coefficients (10–1000 K). *Phys. Chem. Chem. Phys.* **24**, 23593 (2022).

69. J. Lupi, S. Alessandrini, C. Puzzarini, V. Barone, junChS and junChS-F12 models: Parameter-free efficient yet accurate composite schemes for energies and structures of noncovalent complexes. *J. Chem. Theory Comput.* **17**, 6974–6992 (2021).

70. D. Garay-Ruiz, *Visualization Tools for AutoMeKin* (Institute of Chemical Research of Catalonia, 2021).

71. J. Cernicharo, C. Kahane, J. Gómez-González, M. Guélin, Tentative detection of the C_5H radical. *Astron. Astrophys.* **164**, L1 (1986).

72. J. Cernicharo, C. Kahane, J. Gómez-González, Detection of the $^2\Pi_{3/2}$ state of C_5H. *Astron. Astrophys.* **167**, L5 (1986).

73. R. C. Fortenberry, The formation of astromolecule ethynyl cyclopropenylidene (c-C_3HCCH) from C_2H and c-C_3H_2C. *Astrophys. J.* **921**, 132 (2021).

74. A. G. Watrous, B. R. Westbrook, R. C. Fortenberry, Theoretical spectra and energetics for *c*-C_3HC_2H, *l*-C_5H_2, and bipyramidal D_{3h} C_5H_2. *Front. Astron. Space Sci.* **9**, 1051535 (2022).

75. A. M. Mebel, S. H. Lin, X. M. Yang, Y. T. Lee, Theoretical study on the mechanism of the dissociation of benzene. the $C5H_3$ + CH_3 product channel. *J. Phys. Chem. A* **101**, 6781 (1997).

76. K. E. Wilzman, J. S. Ritscher, L. Kaplan, Benzvalene, the tricyclic valence isomer of benzene, *J. Am. Chem. Soc.* **89**, 1031–1032 (1967).
